# Capturing the generation and structural transformations of molecular ions

**DOI:** 10.1038/s41586-023-06909-5

**Published:** 2024-01-10

**Authors:** Jun Heo, Doyeong Kim, Alekos Segalina, Hosung Ki, Doo-Sik Ahn, Seonggon Lee, Jungmin Kim, Yongjun Cha, Kyung Won Lee, Jie Yang, J. Pedro F. Nunes, Xijie Wang, Hyotcherl Ihee

**Affiliations:** 1 Center for Advanced Reaction Dynamics, Institute for Basic Science (IBS), Daejeon, Republic of Korea; 2 Department of Chemistry and KI for the BioCentury, Korea Advanced Institute of Science and Technology (KAIST), Daejeon, Republic of Korea; 3 SLAC National Accelerator Laboratory, Menlo Park, CA USA; 4 Department of Physics and Astronomy, University of Nebraska–Lincoln, Lincoln, NE USA; 5 Present Address: Foundry Business, Samsung Electronics Inc., Hwasung, Gyeonggi Republic of Korea; 6 Present Address: Center of Basic Molecular Science, Department of Chemistry, Tsinghua University, Beijing, China; 7 Present Address: Diamond Light Source, Harwell Science and Innovation Campus, Didcot, United Kingdom

**Keywords:** Reaction kinetics and dynamics, Chemical physics

## Abstract

Molecular ions are ubiquitous and play pivotal roles^1–3^ in many reactions, particularly in the context of atmospheric and interstellar chemistry^4–6^. However, their structures and conformational transitions^7,8^, particularly in the gas phase, are less explored than those of neutral molecules owing to experimental difficulties. A case in point is the halonium ions^9–11^, whose highly reactive nature and ring strain make them short-lived intermediates that are readily attacked even by weak nucleophiles and thus challenging to isolate or capture before they undergo further reaction. Here we show that mega-electronvolt ultrafast electron diffraction (MeV-UED)^12–14^, used in conjunction with resonance-enhanced multiphoton ionization, can monitor the formation of 1,3-dibromopropane (DBP) cations and their subsequent structural dynamics forming a halonium ion. We find that the DBP^+^ cation remains for a substantial duration of 3.6 ps in aptly named ‘dark states’ that are structurally indistinguishable from the DBP electronic ground state. The structural data, supported by surface-hopping simulations^15^ and ab initio calculations^16^, reveal that the cation subsequently decays to *iso*-DBP^+^, an unusual intermediate with a four-membered ring containing a loosely bound^17,18^ bromine atom, and eventually loses the bromine atom and forms a bromonium ion with a three-membered-ring structure^19^. We anticipate that the approach used here can also be applied to examine the structural dynamics of other molecular ions and thereby deepen our understanding of ion chemistry.

## Abstract (web summary)

The use of mega-electronvolt ultrafast electron diffraction combined with resonance-enhanced multiphoton ionization yields data that can reveal the formation and subsequent structural relaxation of a molecular ion on an ultrafast timescale.

## Main

**Fig. 1:**
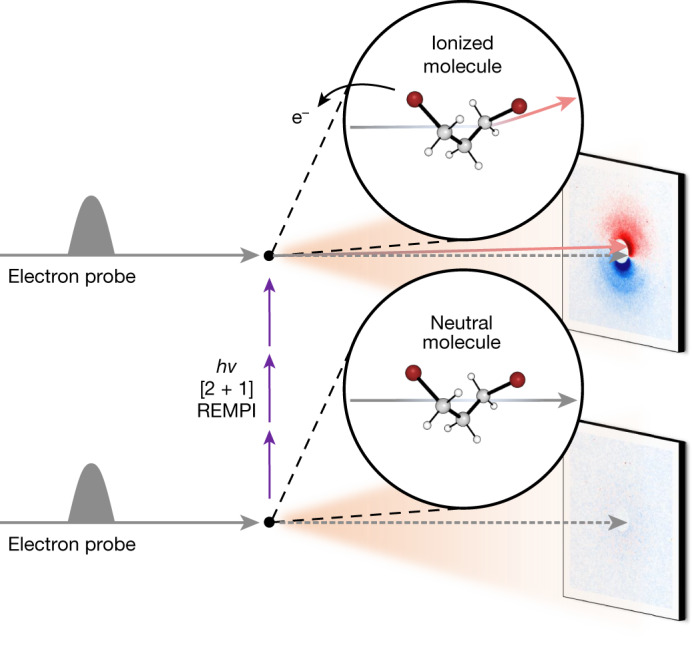
Schematic of the MeV-UED experiment on 1,3-DBP. Photoexcitation by an intense femtosecond ultraviolet laser pulse ionizes 1,3-DBP molecules by means of [2 + 1] REMPI. The molecular structures of 1,3-DBP before and after ionization are captured by using the near-relativistic electron pulse as the probe and measuring the time-resolved diffraction patterns. The sub-picosecond and sub-angstrom temporal and structural resolution of MeV-UED enables direct visualization of the ultrafast structural changes in 1,3-DBP on photoinduced ionization. At a negative time delay, the molecule remains un-ionized and neutral (lower panel) and most of the electrons in the electron beam (grey arrows) travel along a straight path, whereas the remaining electrons undergo diffraction through interaction with the molecules, generating the usual well-centred symmetric diffraction pattern. As the unpumped signal coincides with the signal at the reference time delay (another negative time delay), the difference scattering pattern shown in the lower panel lacks any distinctive features. By contrast, at a positive time delay following ionization and acquisition of a charge (upper panel), the electron beam experiences deflection (red arrow) owing to a slight mismatch between the paths of the electron and laser beams. This deflection causes the electron beam to deviate from its original trajectory (grey arrows), resulting in an off-centred asymmetric diffraction pattern. The difference scattering pattern shown in the upper panel provides information about the structure of the generated ions, as well as its shift owing to the charge of the generated ions. The grey and red arrows in the lower and upper panels depict the paths of the electron beams, highlighting only the trajectory of the direct beam, while omitting the paths of the diffracted beams.

We use in this study MeV-UED, which can directly map molecular structural changes by means of the information contained in time-resolved electron scattering patterns. UED was used to examine ionic species produced by non-resonant strong-field ionization of toluene molecules (see discussion in the Supplementary Information (ref. ^20^)), but extracting structural dynamics proved challenging owing to the intricate nature of simultaneously addressing the following two aspects: (1) achieving soft ionization of molecules to generate a specific, desired ionic species while minimizing undesired fragmentations and (2) producing a considerable quantity of ions. To tackle this challenge, we implemented resonance-enhanced multiphoton ionization (REMPI), a well-established technique known for its soft ionization capabilities and high ionization efficiencies, which can reach up to 10% (ref. ^21^). Specifically, we used [2 + 1] REMPI on 1,3-DBP to investigate the formation of cations and their subsequent structural changes. Quantitative analysis of the experimental scattering data indicated the successful generation of a notable quantity of ionic species, leading to noticeable signals. Detailed information about the structural changes of the ionized DBP molecule was uncovered through the analysis of the UED data collected from the experiment described in Fig. 1.

**Fig. 2:**
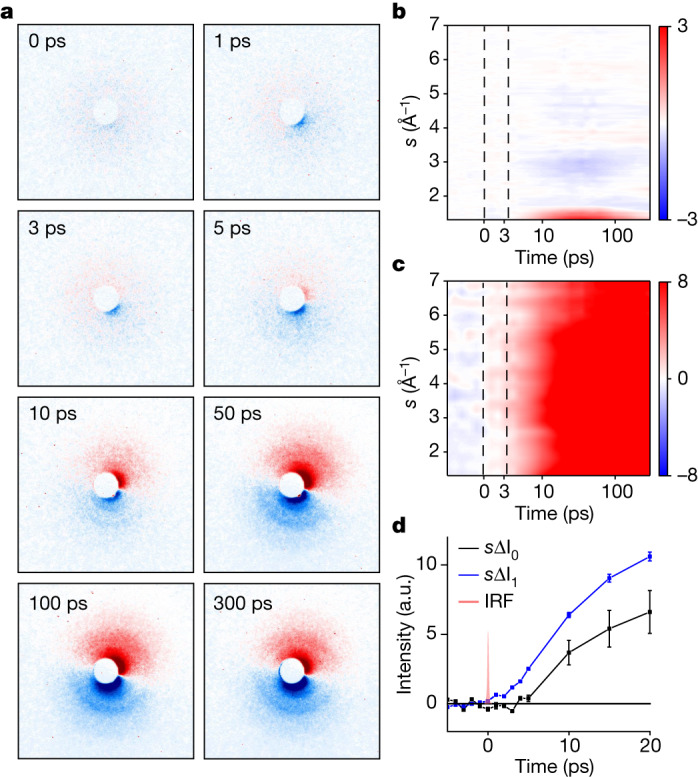
2D difference scattering images and decomposition into the isotropic and asymmetric components. **a**, 2D difference scattering images at selected time delays. The deflection of the electron beam by generated ions results in asymmetry in the scattering images over the azimuthal angle. Each asymmetric image was decomposed into two components: the isotropic component (ΔI_0_) and the asymmetric component (ΔI_1_). **b**,**c**, False-colour plots of *s*ΔI_0_ (**b**) and *s*ΔI_1_ (**c**) extracted from the experimental difference scattering images. To emphasize the signal at high momentum-transfer values, ΔI_0_ and ΔI_1_ were multiplied by the magnitude of the momentum-transfer vector, *s*, to give *s*ΔI_0_ and *s*ΔI_1_. **d**, Comparison of the temporal behaviours of *s*ΔI_0_ and *s*ΔI_1_. The low-*s* region (*s* = 1.3–2.3 Å^−1^) of *s*ΔI_0_ and *s*ΔI_1_ was integrated for each time delay, and the profiles of these integration values are shown. The vertical bar at each point indicates one standard deviation. The instrument response function (IRF) is also indicated. The plots show that the ionization, contributing to *s*ΔI_1_, occurs instantly on photoexcitation, whereas notable structural changes of the ionized species, contributing to *s*ΔI_0_, occur gradually after an induction period of approximately 4 ps. a.u., arbitrary units. Source Data

**Extended Data Fig. 1:**
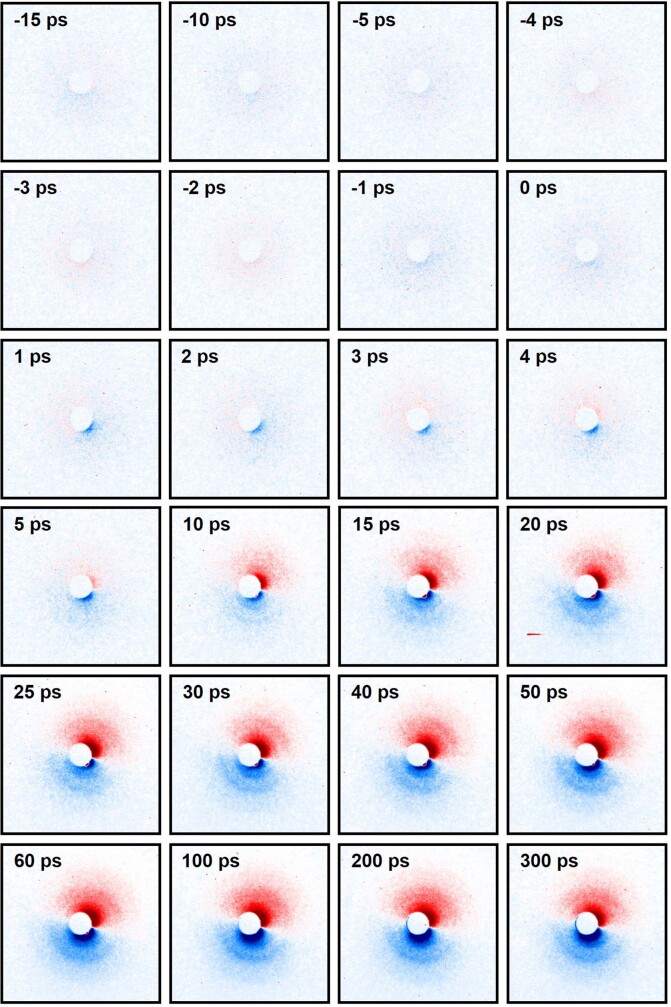
2D difference scattering patterns at all time delays. The 2D difference scattering patterns show the changes in the scattering intensities over time. The patterns, which contain structural information and beam shifts induced by the ion formation, were analysed using the Legendre decomposition to separate the isotropic structural information (ΔI_0_) and anisotropic ion formation signal (ΔI_1_).

In the MeV-UED experiment, the ionization of DBP was initiated using a pump pulse with a wavelength of 267 nm. Figure 2a shows selected difference scattering patterns at various time delays, whereas the patterns for all time delays are shown in Extended Data Fig. 1. The signals are proportional to the laser fluence with the order of 2.7 ± 0.1, confirming that the UED signal reflects the reaction intermediates formed by means of a three-photon process^22^, namely, [2 + 1] REMPI (see the ‘Data collection’ section in Methods for details).

**Extended Data Fig. 2:**
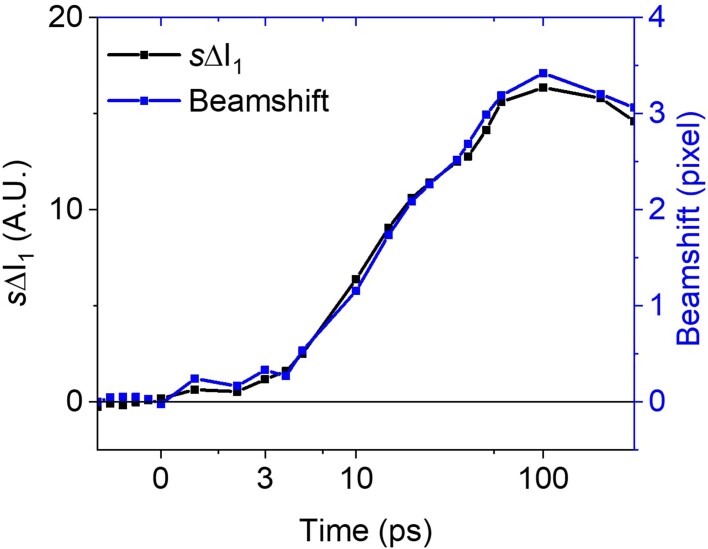
Comparison of the beam shift and the *s*ΔI_1_ curves extracted using Legendre decomposition. The electron beam shifts as it interacts with the ions generated by the pump pulse. The electron-beam shift is an artefact that needs to be addressed in extracting structural information from the scattering signal. The effect of the beam shift can be separated as the *s*ΔI_1_ curves through Legendre decomposition, which was used to decompose the original difference pattern into one for the scattering signal from the molecular structural changes (ΔI_0_(*s*,*t*)) and the other for the signal owing to the ionic-species-induced beam deflection (ΔI_1_(*s*,*t*)). The beam shifts as a function of time overlap well with the time-dependent intensities of *s*ΔI_1_, which are integrated values of *s*ΔI_1_ over the 1.3 < *s* < 2.0 Å^−1^ region. This confirms that the Legendre decomposition effectively removes the effects of the beam shift. Source Data

The difference scattering pattern was decomposed into two components using Legendre decomposition, one for the scattering signal from the molecular structural changes (ΔI_0_(*s*,*t*)) and the other for the signal owing to the ionic-species-induced beam deflection (ΔI_1_(*s*,*t*)) (see the Supplementary Information for details). Figure 2b depicts the resulting *s*ΔI_1_(*s*,*t*). The time trace of its amplitude, presented in Fig. 2d and Extended Data Fig. 2, demonstrates a rapid increase immediately after time zero, indicating the formation of ions, consistent with rapid photoinduced ionization (<40 fs)^23^. By contrast, *s*ΔI_0_(*s*,*t*), shown in Fig. 2c, starts showing noticeable signals only after an unusually long induction period of approximately 4 ps. Because the absence of a difference signal in *s*ΔI_0_(*s*,*t*) implies that there is no change in molecular structure, the data indicate that there is no immediate structural response to ionization and that structural changes occur only after 4 ps. The initially generated ions thus have a molecular structure identical to that of the reactant neutral molecule, within the current signal-to-noise ratio. The long induction period we see is uncommon and has not been observed in a previous time-resolved study of DBP that used mass spectrometry^24^ (see further discussion in the ‘Comparison of the observed induction period with previous studies’ section in the Supplementary Information).

**Fig. 3:**
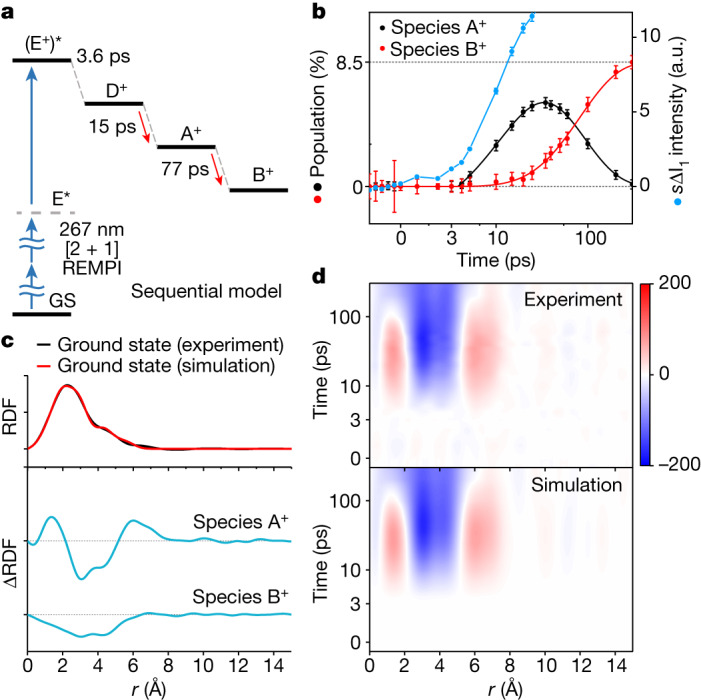
Extraction of the kinetics and RDFs from the experimental data. **a**, The kinetic model used for the analysis, based on the two components (species) and two decay constants obtained from SVD analysis on the UED data (Extended Data Fig. 3). The sequential model provides the best fit for our experimental results (details shown in Extended Data Figs. 3 and 4). **b**, Time-resolved populations of species A^+^ and B^+^ and the intensity of *s*ΔI_1_(*s*,*t*). The error bars for populations are multiplied by 10 for clear visualization. Solid lines represent the kinetics of *s*ΔI_0_(*s*,*t*) based on the kinetic model in **a** and dots with one-standard-deviation error bars are obtained by independently fitting the experimental data for each time delay as a linear combination of species-associated difference scattering curves. **c**, RDF for the ground state and the difference RDF (ΔRDF) for species A^+^ and B^+^. The experimentally obtained RDF(*r*) for the ground state (black) is compared with the theoretically calculated one (red). **d**, Comparison of experimentally obtained ΔRDF(*r*,*t*) with the simulated ΔRDF(*r*,*t*) based on the best-fit kinetic model determined from the kinetic analysis. a.u., arbitrary units. Source Data

To obtain detailed kinetic information, we conducted kinetic analysis on *s*ΔI_0_(*s*,*t*). First, we performed singular value decomposition (SVD), which decomposes the original data into time-invariant features (left singular vectors, LSVs), their relative contributions (singular values) and their time profiles (right singular vectors, RSVs)^25^ (see the ‘Singular value decomposition’ section in the Supplementary Information for details). The SVD of *s*ΔI_0_(*s*,*t*) shows that the experimental data comprise two primary components. For the quantitative analysis, we fit the two RSVs globally using a sum of an induction time and two exponential functions. The time constants for two RSVs were shared, resulting in a satisfactory fit with two exponential time constants of 15 ± 2 ps and 77 ± 15 ps and an induction period of 3.6 ± 0.3 ps. On this basis, we performed kinetics-constrained analysis (KCA)^25,26^ and the results are shown in Fig. 3. To explain an induction period, the kinetic model contains (E^+^)*, which represents the transient excited state of the ion generated on photoexcitation, and, after the induction period, (E^+^)* relaxes to the D^+^ ion. Both (E^+^)* and D^+^ are structurally dark states, whose molecular structure is indistinguishable from that of the ground state of the neutral DBP, within the signal-to-noise ratio of the current MeV-UED experiment (see the ‘Details of the kinetic analysis using SVD’ section in the Supplementary Information for details). Among two kinetic models that satisfy the conditions of having three kinetic components (two decay constants and one induction time constant) and two kinetic species, a sequential model in which the first species (A^+^) is formed from D^+^, followed by its conversion to the second species (B^+^), explains *s*ΔI_0_(*s*,*t*) better than the other, parallel model, in which two intermediates are generated from the D^+^ simultaneously (Extended Data Fig. 4). Figure 3a shows the diagram of the final determined sequential model and Fig. 3b shows the population dynamics of all species involved in the reaction. Using the optimized kinetic model, which is shown in Fig. 3a, obtained from the KCA, we fit ΔI_0_(*s*,*t*) to extract species-associated difference scattering curves (SADS(*s*)) for all species using linear combination fitting. Figure 3b illustrates the population changes of intermediates. A SADS(*s*) in *s*-space can be converted into a difference radial distribution function (ΔRDF(*r*)) in the real space through a sine Fourier transformation (see Fig. 3c). By using the time-dependent concentrations and two ΔRDF(*r*)s of A^+^ and B^+^ (Fig. 3b,c), we can reconstruct ΔRDF(*r*,*t*) for all time delays. These reconstructed ΔRDF(*r*,*t*)s satisfactorily reproduce the experimental ΔRDF(*r*,*t*) for all time delays, as shown in Fig. 3d and Supplementary Fig. 6, demonstrating that our kinetic model accurately describes the experimental data.

**Fig. 4:**
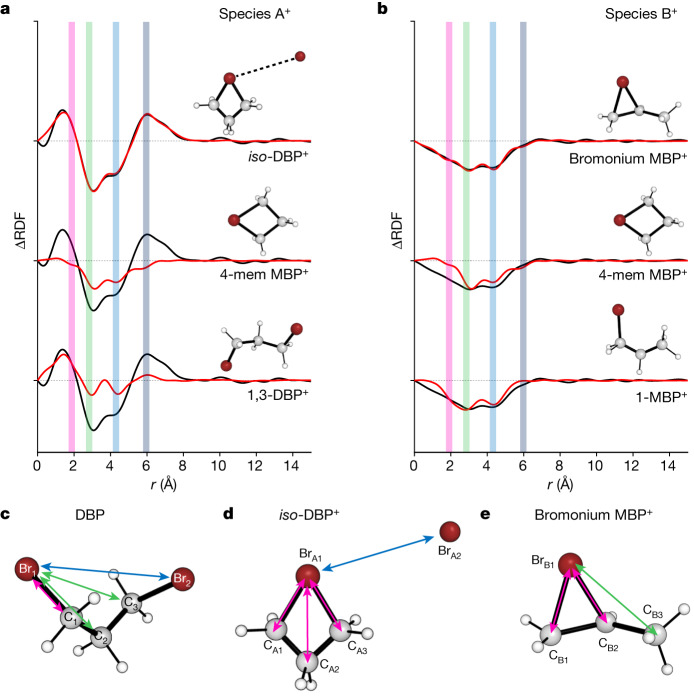
Structural analysis of species A^+^ and B^+^. **a**,**b**, Structural refinements for various candidate structures in the real space, ΔRDF(*r*). The experimental ΔRDF(*r*) (black) and theoretical ΔRDF(*r*) (red) calculated for three candidate structural models of species A^+^ (**a**) and species B^+^ (**b**) are shown. The DFT-optimized structures were further refined to minimize the discrepancy between the experimental and theoretical data during the refinement process (fitted results in *s*-space are in Extended Data Fig. 6). For species A^+^, the *iso*-DBP^+^ model (top), which considers a Br radical loosely bound to a four-membered ring MBP^+^ moiety, fits the UED data much better than the other models, the model considering a complete dissociation of a Br radical (middle, 4-mem MBP^+^) and the model considering a minimum structural deviation from that of the ground-state 1,3-DBP (bottom, 1,3-DBP^+^). For species B^**+**^, the bromonium MBP^+^ model (top) featuring Br with bridging character as a bromonium ion provides a much better fit to our UED data than the other two models, 4-mem MBP^+^ (middle) and MBP^+^ with Br in a non-bridging configuration (bottom, 1-MBP^+^). **c**–**e**, The refined structures of the ground-state DBP (**c**), species A^+^ (**d**) and species B^+^ (**e**). Key atomic pairs contributing markedly to the ΔRDF(*r*) are highlighted with coloured arrows and their distances are indicated by bans of the same colour in **a** and **b**. Source Data

Next we qualitatively analysed the structural features of the two species by examining their ΔRDF(*r*). As detailed in the Supplementary Information, this qualitative analysis already shows that (1) ΔRDF(*r*) of A^+^ with a peak at an unusually long distance of about 6 Å suggests that the most probable form of A^+^ is DBP^+^ with a loosely bound Br and (2) ΔRDF(*r*) of B^+^, lacking positive peaks, indicates that it corresponds to MBP^+^, for which the loosely bound Br is eventually dissociated. To quantitatively analyse the changes in ΔRDF(*r*,*t*) that represent structural changes before and after a reaction, the static RDF(*r*) of the ground state was analysed to first determine the fractions of conformers (GG, AG and AA, with their geometric parameters listed in Supplementary Table 3). According to the analysis (Supplementary Fig. 7), the ground-state DBP exists in the ratio of 66 ± 2%:20 ± 2%:14 ± 3% for GG:AG:AA, which is similar to the ratio from previous studies (67%:30%:3%)^27^. On the basis of the static analysis of the ground-state DBP, we extracted structural information on A^+^ and B^+^ by quantitatively analysing their SADS(*s*) (Extended Data Fig. 6). To do so, we investigated several candidate models for the structure of the transient ionic species using density functional theory (DFT) calculations (Fig. 4). For A^+^, we tested two cationic DBP structures, *iso*-DBP^+^ and 1,3-DBP^+^, and one cationic MBP structure, 4-mem MBP^+^, that is, MBP^+^ with a four-membered ring. For B^+^, we tested three cationic MBP structures, bromonium MBP^+^, 4-mem MBP^+^ and 1-MBP^+^. Starting from the optimized candidate structures obtained from the DFT calculations, we refined the structures through a global fitting approach to simultaneously fit the experimentally measured SADS(*s*) for species A^+^ and B^+^. Figure 4 shows the results of the structural refinement for various candidate structures in the real space, ΔRDF(*r*). For A^+^, *iso*-DBP^+^ gives the best agreement with the experimental ΔRDF(*r*) (Fig. 4a, top). Specifically, *iso*-DBP^+^ depicts a dissociated Br atom bound to the MBP^+^ molecule, which possesses a four-membered ring structure, with the dissociated Br atom maintained at a long distance (*r* ≈ 5.9 Å) from the Br atom of MBP^+^ (Fig. 4d). The other two candidate structural models (4-mem MBP^+^ and 1,3-DBP^+^ in Fig. 4a) lack the long atomic pair distance of approximately 5.9 Å and are therefore unable to accurately fit the ΔRDF(*r*) of A^+^. A discussion about the loosely bound nature of *iso*-DBP^+^ is provided in the ‘Loosely bound nature of *iso*-DBP^+^ supported by calculated vibrational frequencies’ section in the Supplementary Information. The C_A1_–Br_A1_ bond distance was optimized to be 1.76 ± 0.01 Å, which is contracted compared with the typical C–Br distance observed in neutral DBP. The contraction can be attributed to the strong interaction between the negatively charged carbon and positively charged Br (Supplementary Table 4). A notable feature is also observed in *r*C_A1_C_A2_, which has a substantially shorter value (1.28 ± 0.03 Å) than the known bond length of 1.5 to 1.6 Å for a C–C single bond. These indicate that *iso*-DBP^+^ has stronger C–Br and C–C bonds than a typical neutral molecule. For B^+^, bromonium MBP^+^ best describes the experimental ΔRDF(*r*) (Fig. 4b, top). It has a Br atom forming a triangle with two C atoms, corresponding to a well-known halonium ion structure. The other candidates (4-mem MBP^+^ and 1-MBP^+^) were unable to satisfactorily explain the features of ΔRDF(*r*) at the low *r* (*r* < 3.0 Å) region. Although bromonium MBP^+^ has a *r*C_B3_Br_B1_ of 1.96 ± 0.01 Å, which is similar to the C–Br distance (2.0 Å) of the ground-state DBP, *r*C_B1_C_B2_ (1.74 ± 0.05 Å) and *r*C_B2_C_B3_ (1.68 ± 0.05 Å) were found to be longer than the typical C–C distance (Fig. 4e). Such C–C bond elongation can occur in cationic molecules, as it leads to a decrease in bond order owing to the positive charge^28^. The fitted parameters for the optimized structures obtained from the simultaneous fitting of the SADSs of the two species (*iso*-DBP^+^ and bromonium MBP^+^) are listed in Supplementary Table 1. The conformer fractions of the neutral DBP were also used as fitting parameters and the optimized fractions (63 ± 3%:20 ± 4%:17 ± 5% for GG:AG:AA) are highly similar to those obtained from the fitting of the static curve of DBP (66 ± 2%:20 ± 2%:14 ± 3%).

A bromonium ion is a well-known intermediate formed during the addition reaction of bromine to an alkene species with a C–C double bond, but its structure has not been directly determined. Instead, the bromonium ions were stabilized in the salt form in crystals and their structures were determined through crystallography^19^. The gas-phase structure of bromonium MBP^+^ determined through UED provides the reference for comparison with those in crystals. The comparison reveals that the structure of the bromonium cation as an intermediate in the chemical reaction differs substantially from the structure of the bromonium salt in the crystal in its stable form: in the presence of the counterion in the crystal, *r*C_B1_C_B2_ is 1.50 Å and *r*C_B1_Br_B1_ is 2.1–2.2 Å, whereas their gas-phase counterparts are 1.74 ± 0.05 Å and 1.96 ± 0.01 Å, respectively.

The presence of an induction period implies that the ion species during this state maintains its molecular structure and conformer ratios (see the ‘Existence of the induction period’ section in the Supplementary Information for details). Furthermore, the induction period provides valuable insight into the molecular structure of the excited DBP population in the Rydberg state generated by two-photon absorption, as discussed in the ‘Structure of the Rydberg state’ section in the Supplementary Information.

**Fig. 5:**
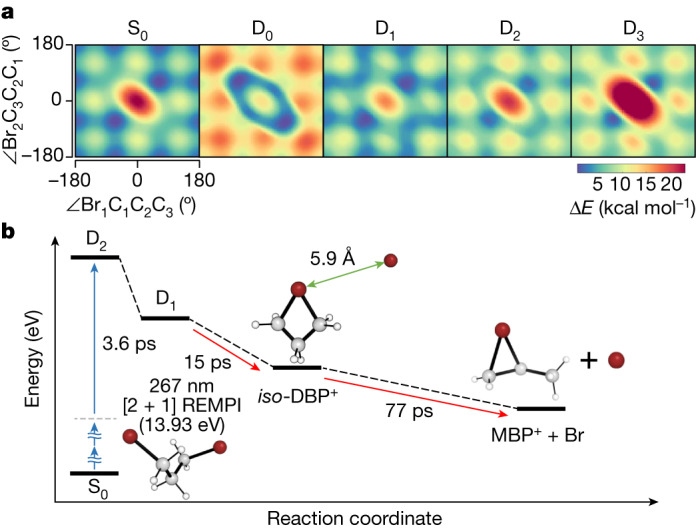
PESs to provide insights into the excited-state dynamics of DBP^+^. **a**, PESs of S_0_, D_0_, D_1_, D_2_ and D_3_ using XMS-CASPT2 corrections of the SA-CASSCF(8,8) energies. All the PESs in **a** are plotted on the basis of the energy difference (Δ*E*) in comparison with the global minima of each state. The functions of the Br_1_–C_1_–C_2_–C_3_ and Br_2_–C_3_–C_2_–C_1_ dihedral angles depicted in the PESs offer insights into identifying the cationic excited state that initiates the induction period. **b**, Overall structural dynamics of DBP followed by the [2 + 1] REMPI process. Initially, no substantial structural change is observed for a period of about 3.6 ps. Afterwards, a transient species, *iso*-DBP^+^, is formed with a time constant of 15 ps. Finally, the loosely bound Br in *iso*-DBP^+^ escapes, yielding the bromonium MBP^+^ with a time constant of 77 ps. Source Data

To corroborate the observed photoreaction pathways that involve a long induction period on photoexcitation^29,30^ and subsequent Br dissociation, we performed ab initio calculations at various levels of theory (details in the Supplementary Information). First, we explored the potential energy surfaces (PESs) of the electronic ground state (S_0_) of DBP and the first four doublet states (D_0_, D_1_, D_2_ and D_3_) of DBP^+^ by using the extended multistate complete active space second-order perturbation theory (XMS-CASPT2) method. The resulting PESs, drawn as functions of the Br_1_–C_1_–C_2_–C_3_ and Br_2_–C_3_–C_2_–C_1_ dihedral angles (Fig. 5a) and as functions of the C–Br distance (Supplementary Fig. 13), provide clues for assigning which cationic excited state is responsible for the initial induction period. PESs of DBP and DBP^+^ show remarkable similarities, indicating that DBP^+^ generated at the Franck–Condon region is at a local or global minimum in all doublet states and thus likely to retain the structure identical to that of S_0_ before transitioning to *iso*-DBP^+^. Furthermore, the norms of the Dyson orbitals (Extended Data Table 1), representing the ionization strength from the Rydberg state, highlight that, among D_0_, D_1_ and D_2_ states, which exhibit relatively large norms for at least one conformer, only D_2_ shows similar norms across all three conformers. On the basis of these considerations, we conclude that D_2_, characterized by PESs similar to those of S_0_ and substantial transition rates from the Rydberg state for all three conformers, is the most probable candidate for the initially populated state, (E^+^)*. To investigate the dynamics from D_2_ state to *iso*-DBP^+^, we calculated the conversion yields of radiative and nonradiative pathways from D_2_ state to D_0_. As a result, neither of the pathways were probable, as the lifetime of the former was too long (approximately 1 μs), as shown in Supplementary Table 6, and the thermal energy of the latter was too high, which is not compatible with the observed data. Detailed information can be found in the ‘Reaction pathways from D_2_ state to *iso*-DBP^+^’ section in the Supplementary Information. Therefore, we suggest that the most probable route for the formation of *iso*-DBP^+^ starts from D_2_ to D_1_, followed by a conical intersection connecting D_1_ to *iso*-DBP^+^ (Fig. 5b). The interpretation of the Dyson orbitals not only facilitates the estimation of transition probabilities but also offers a chemically insightful explanation for the absence of notable structural changes on ionization. As illustrated in Supplementary Fig. 11, these orbitals reveal that, during the cation-formation process, an electron is ejected from an orbital predominantly localized on the bromine atom. Notably, this specific orbital demonstrates nonbonding character, with minimal involvement in the bonding interactions between bromine and carbon atoms. Consequently, the removal of an electron from this orbital exerts only a negligible influence on the molecular structure.

To obtain further support for the observed induction period, surface-hopping simulations were carried out up to 1 ps, considering D_2_ as the initial active state. The trajectories show that DBP^+^ does not exhibit noticeable structural changes for several hundred femtoseconds after excitation, as evidenced by the computed averaged difference scattering curve (Extended Data Fig. 7). Comprehensive discussions are provided in the ‘Results of surface hopping simulations’ section of the Supplementary Information. Furthermore, we conducted intrinsic reaction coordinate (IRC) calculations to enhance our understanding of the reaction dynamics (Extended Data Fig. 8 and Supplementary Fig. 18). These calculations involved the determination of transition states and an intermediate species, and the results of vibrational frequency calculations for the transition states and intermediate species are provided in Supplementary Tables 7 and 8. On the basis of the results of IRC calculations, we conducted calculations to locate a conical intersection (CI1) that connects the D_1_ state to *iso*-DBP^+^. A detailed description of the computational methods is provided in the ‘Computational details’ and ‘Details of surface hopping simulation’ sections in the Supplementary Information. The plausible reaction coordinates and pathways for the formation of *iso*-DBP^+^ and MBP^+^ can be proposed as detailed in the ‘Reaction pathways to form *iso*-DBP^+^ and MBP^+^’ section in the Supplementary Information.

By introducing a protocol to generate ions suitable for time-resolved scattering and to analyse scattering patterns from ionized species, this study represents a notable step forward in understanding the ultrafast structural dynamics of ionic species in the gas phase. This research addresses a previously unexplored area of study owing to experimental limitations and lays the foundation for identifying the structural dynamics of ions.

## Methods

### Data collection

The data were collected using the MeV-UED facility at the SLAC National Accelerator Laboratory^12,31–33^. The kinetic energy of the electron pulses is 3.7 MeV, and each pulse contains approximately 10^4^ electrons, focused to a diameter of 200 µm full width at half maximum (FWHM). The sample, 1,3-DBP (99%), was purchased from Sigma-Aldrich and used without further purification. The gas was introduced into the vacuum chamber with a flow cell 100-µm nozzle and the nozzle was heated to 40 °C to prevent clogging of the molecules. The electron and laser beams were aligned to co-propagate with a 5° angle, intersecting the gas jet at roughly 250 µm underneath the nozzle exit. The overall instrumental response was estimated to be around 104 fs FWHM (Supplementary Fig. 2). Diffraction patterns at each time delay were accumulated for 20 min. For photoexcitation, a 565-µJ pump laser pulse with a centre wavelength of 267 nm and a 1.3 nm FWHM bandwidth was linearly polarized and elliptically focused to a spot of dimensions 280 × 200 µm^2^, giving a fluence of 1,200 mJ cm^−2^. The electron detector consisted of a P43 phosphor screen with a centre hole, a 45° mirror with a centre hole, an imaging lens and an electron-multiplying charge-coupled device (see the Supplementary Information for details).

For the MeV-UED experiment, the ionization of DBP was initiated using a pump pulse with a wavelength of 267 nm. Despite its low single-photon absorption cross-section (4.73 × 10^−20^ cm^2^) at 267 nm (Supplementary Fig. 1), strong pump laser intensity can cause substantial photoinduced ionization through [2 + 1] REMPI (ref. ^22^). After a time delay (Δ*t*), a 3.7-MeV electron pulse (probe) was sent to the sample and the structural changes of ionic species over time were observed by measuring the diffracted patterns of scattered electrons on a 2D area detector (details in the Supplementary Information). Figure 2a shows selected difference scattering patterns at various time delays, whereas the patterns for all time delays are shown in Extended Data Fig. 1. The time-resolved difference patterns show negligible signals at negative time delays, gradually showing a clear signal as time passes after time zero. At negative time delays, the electron-beam profile and difference patterns are isotropic, whereas at positive time delays, they exhibit asymmetric features with respect to the beam centre (Extended Data Fig. 1), indicating the generation of ionic species capable of affecting the beam path of electrons^34^. The signals are proportional to the laser fluence with the order of 2.7 ± 0.1 (Supplementary Fig. 3), confirming that the UED signal reflects the reaction intermediates formed by means of a three-photon process, namely, [2 + 1] REMPI (ref. ^22^).

### Data processing and analysis

The scattering patterns (Extended Data Fig. 1) are highly asymmetric and exhibit notable positional shifts of the undiffracted electron beam, unlike typical ones observed in UED data from neutral intermediates. The asymmetry arises from the e-beam deflection caused by the Coulomb interaction with the ion product. As this artefact generated by the beam deflection can distort the structural information of the molecule^34^, it is a hindering factor in extracting the structural information of transient species from the diffraction-pattern analysis. To remove the artefact corresponding to cos*θ* in the low-*s* region, we introduced a method that removes the existing anisotropic scattering signal based on symmetry^35,36^.

To remove the beam-shift effect caused by the formation of cations and eliminate the high-*s* side artefact, we used Legendre decomposition, a decomposition based on the Legendre series. The difference scattering image was decomposed using Legendre decomposition into a linear combination of two components with scattering intensity distributions of the zeroth-order and first-order Legendre polynomials along the *φ* angle, the zeroth-order term (ΔI_0_) and the first-order term (ΔI_1_), respectively. The relationship between the original difference scattering image and the three terms obtained from the decomposition is expressed by the following equation:

1 $$\Delta {\rm{I}}\left(s,\varphi ,t\right)\propto \Delta {{\rm{I}}}_{0}\left(s,t\right)+{{\rm{P}}}_{1}\left(\cos \,\varphi \right)\Delta {{\rm{I}}}_{1}(s,t)$$

In this equation, *s* is the momentum transfer vector, P_1_ is the first-order Legendre polynomial and *φ* is the azimuthal angle on the charge-coupled device plane. The momentum transfer vector *s* is defined as follows:

2 $$s=\frac{4\pi }{\lambda }\sin \left(\frac{\theta }{2}\right)$$

in which *λ* is the de Broglie wavelength of the incident electrons and *θ* is the angle between the incident and scattered electrons.

To ensure that the shift of the electron beam has a minimal influence on both the shape of the ΔI_0_ curves and the structural parameters derived from the analysis of ΔI_0_, we tested the effect of beam shifts on our correction method (Supplementary Figs. 4 and 5 and Supplementary Table 2). Detailed validation and discussion on the decomposition method are presented in the Supplementary Information.

### General scheme for the kinetic analysis using SVD

To extract kinetics information of intermediates and their structures from ΔI(*s*,*t*), we followed the well-established procedure, which had been applied to time-resolved X-ray liquidography studies on small molecules, consisting of kinetic analysis using SVD (refs. ^25,26^).

**Extended Data Fig. 3:**
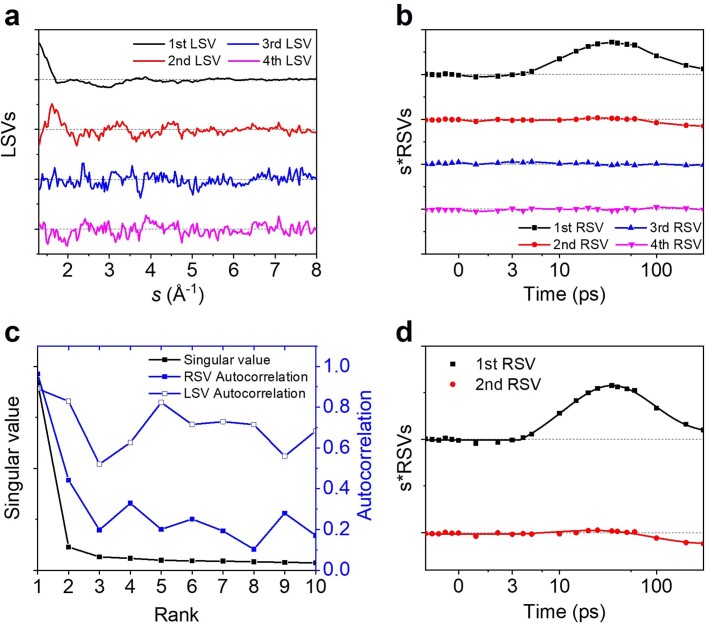
SVD results of MeV-UED data for DBP. **a**,**b**, The first four left (**a**) and right (**b**) singular vectors (LSVs and RSVs) of ΔI_0_(*s*,*t*). The RSVs are weighted by the corresponding singular values. **c**, The singular values and the autocorrelation values for RSVs and LSVs. The singular values and autocorrelation values indicate that there are two signal components contributing notably to ΔI_0_(*s*,*t*). **d**, Temporal profiles of two notable components fitted with two exponential functions (*t*_1_ = 15 ± 2 ps, *t*_2_ = 77 ± 15 ps) with a constant for the induction period (*t*_d_ = 3.6 ± 0.3 ps). Source Data

**Extended Data Fig. 4:**
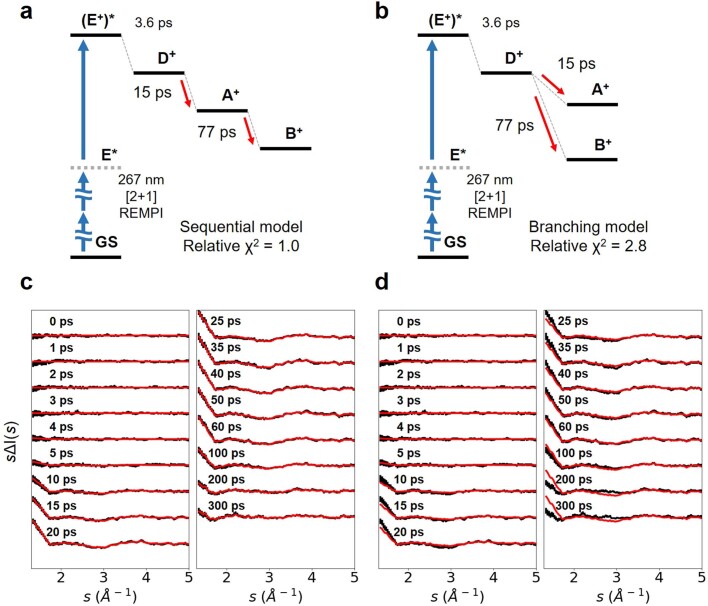
Results of kinetic analysis. **a**,**b**, Kinetic models for the two scenarios with the two species and two time constants, a sequential model (**a**) and a branching model (**b**). **c**,**d**, Time-resolved difference scattering curves, *s*ΔI_0_(*s*,*t*), (black curves) and theoretical curves (red curves) generated by linear combinations of LSVs based on the sequential model (**a**) and the branching model (**b**). The calculated curves from the sequential model show better agreement with the experimental data. Source Data

We obtained clues about the number of intermediates associated with temporal dynamics by performing the SVD analysis on the whole data. The LSVs from the SVD analysis are shown in Extended Data Fig. 3a,b. We fitted the two notable RSVs with exponential functions and one induction delay constant. The exponential fitting results are shown in Extended Data Fig. 3d. Two notable components are fitted with the one induction delay (*t*_d_ = 3.6 ± 0.3 ps) and two exponential functions (*t*_1_ = 15 ± 2 ps, *t*_2_ = 77 ± 15 ps). Then, we conducted KCA, which is a method for generating theoretical difference scattering curves using time-dependent population changes of the intermediates expressed with a set of variable kinetic parameters, with an assumed candidate kinetic model from the results of SVD on the data matrix^25,26^ (Extended Data Fig. 4). Further details on the kinetic analysis using SVD can be found in the ‘Details of the kinetic analysis using SVD’ section in the Supplementary Information.

### Generation of theoretical static diffraction curve

Theoretical scattering curves used for the structural analysis were calculated under the independent atom model. In this model, the static diffraction curve, I(*s*), can be expressed as the sum of the interference terms for all atomic pairs, the molecular scattering, I_mol_(*s*), and the sum of the atomic scattering, I_at_(*s*), for each atom in the molecule. A detailed explanation of the process for calculating the theoretical static difference curve, along with the relevant equations, can be found in the ‘Detailed procedure for generating theoretical static diffraction curve’ section in the Supplementary Information.

### Generation of difference signals, *s*ΔI(*s*,*t*), Δ*s*M(*s*,*t*) and ΔRDF(*r*,*t*)

We use the difference-diffraction method^37^ to calculate the difference signal, sometimes referred to as *s*ΔI(*s*), and difference *s*M(*s*,*t*) and RDF(*r*,*t*), Δ*s*M(*s*,*t*) and ΔRDF(*r*,*t*), respectively. A detailed method for calculating the difference signals, accompanied by the relevant equations, is described in the ‘Detailed procedure for generating difference signals’ section of the Supplementary Information.

### Structure refinements by structural fitting analysis

**Extended Data Fig. 5:**
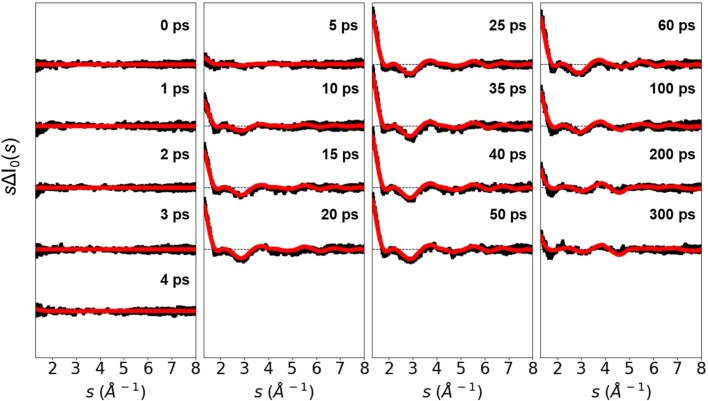
Fit results for the isotropic difference scattering curves (*s*ΔI_0_(*s*)). Experimental curves with the corresponding standard errors (vertical bars) and the theoretical curves are shown in black and red lines, respectively. Source Data

Through structural fitting analysis, we determined the structures of the intermediates (species A^+^ and B^+^) that best describe the experimentally measured difference scattering curves (see Extended Data Fig. 5). The detailed procedure is described in the ‘Details of structural refinement’ section of the Supplementary Information, along with related figures in Supplementary Figs. 8 and 9.

### Structural analysis

We extracted structural information on species A^+^ and B^+^ by quantitatively analysing their SADS(*s*). Further details on the structural analysis process can be found in the ‘Details of structural analysis’ section of the Supplementary Information.

### Potential energy profiles

A series of relaxed PES scans were calculated for the ground state as well as the first four ionic doublet states (D_0_, D_1_, D_2_ and D_3_) using XMS-CASPT2 corrections applied to the SA-CASSCF(8,8) (state-averaged complete active space self-consistent field) energies. The resulting PESs are shown in Fig. 5a and Supplementary Fig. 10a, whereas the corresponding complete active space orbitals can be found in Supplementary Fig. 12. The PESs obtained with CAM-B3LYP functionals are shown in Supplementary Fig. 10b for comparison. The PESs for 1,3-DBP are compared with those of other molecules with similar structures, including 1,3-dichloropropane and propane, in Extended Data Fig. 9. Further details on the PES scans can be found in the ‘Details of potential energy scans’ section of the Supplementary Information.

### Dyson orbitals

Dyson orbitals were used to determine which DBP^+^ doublet state is more readily populated after the ionization of the DBP molecule. We calculated the Dyson orbitals between the first Rydberg state and the first eight doublet states^38,39^. Further details on the calculation and interpretation of Dyson orbitals can be found in the ‘Details of Dyson orbitals’ section of the Supplementary Information.

### Surface-hopping simulation

To investigate the reasons behind the induction time, we performed surface-hopping simulations and examined the reaction pathway and transition rate of DBP^+^ (refs. ^40,41^). For the most stable conformer of DBP, the simulations were carried out up to 1 ps, considering D_2_ as the initial active state (Supplementary Figs. 14–17). The details are presented in the ‘Details of surface hopping simulation’ section in the Supplementary Information.

## Online content

Any methods, additional references, Nature Portfolio reporting summaries, source data, extended data, supplementary information, acknowledgements, peer review information; details of author contributions and competing interests; and statements of data and code availability are available at 10.1038/s41586-023-06909-5.

### Supplementary information

Supplementary Information This file contains Supplementary Methods, Supplementary Discussion and Supplementary References; it includes 18 Supplementary Figures and 8 Supplementary Tables.

### Source data

Source Data Fig. 2

Source Data Fig. 3

Source Data Fig. 4

Source Data Fig. 5

Source Data Extended Data Fig. 2

Source Data Extended Data Fig. 3

Source Data Extended Data Fig. 4

Source Data Extended Data Fig. 5

**Extended Data Fig. 6:**
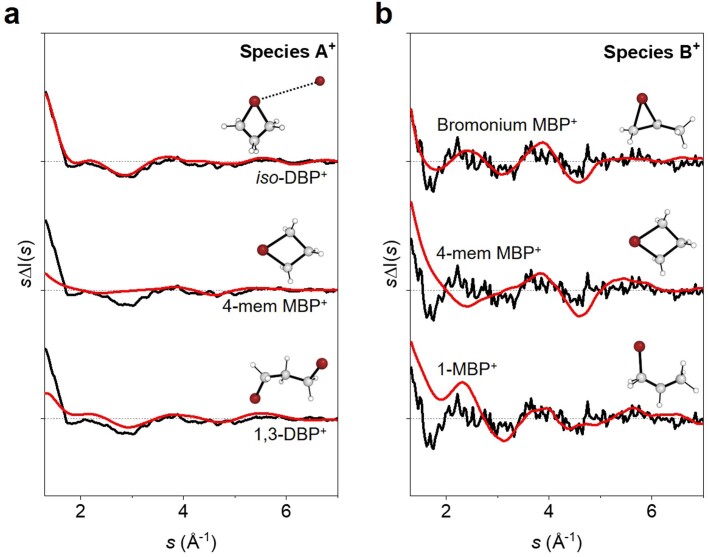
Structural analysis of species A^+^ and B^+^. **a**,**b**, Structural refinements for various candidate structures in *s*-space. Experimentally measured SADSs (*s*ΔI_0_(*s*)), which were extracted from the kinetic analysis, and theoretically calculated difference scattering curves are presented in black and red lines, respectively. The comparison between the experimental and theoretical curves calculated for three candidate structural models of species A^+^ (**a**) and species B^+^ (**b**) are shown. The DFT-optimized structures were further refined to minimize the discrepancy between the experimental and theoretical data during the refinement process. For species A^+^, the *iso*-DBP^+^ model (top), which considers a Br radical loosely bound to a four-membered ring MBP^+^ moiety, fits the UED data much better than the other models, the model considering a complete dissociation of a Br radical (middle, 4-mem MBP^+^) and the model considering a minimum structural deviation from that of the ground state 1,3-DBP (bottom, 1,3-DBP^+^). For species B^+^, the bromonium MBP^+^ model (top) featuring Br with bridging character as a bromonium ion provides a much better fit to our UED data than the other two models, 4-mem MBP^+^ (middle) and MBP^+^ with Br in a non-bridging configuration (bottom, 1-MBP^+^). Source Data

Source Data Extended Data Fig. 6

**Extended Data Fig. 7:**
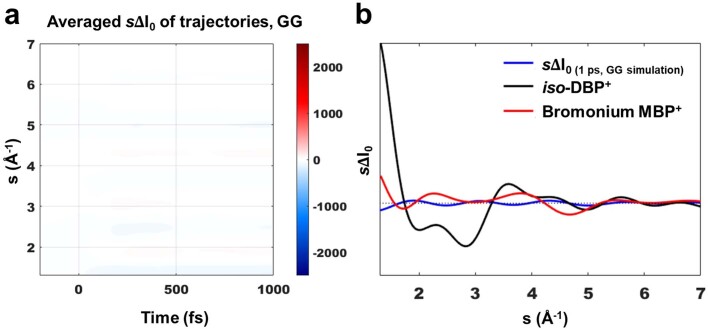
Simulated difference scattering curves (*s*ΔI_0_) using trajectories from surface-hopping simulation. **a**, Simulated *s*ΔI_0_(*s*,*t*) using the trajectories starting from the GG conformer of DBP. The simulated curves are based on surface-hopping simulation and represent the averaged dynamics of DBP^+^ within 1 ps after photoexcitation. The instrumental response function of the MeV-UED instrument, which is approximately 100 fs, was used to describe the signal around time zero for the simulated curves. Colour scales in the figures represent the amplitude of the signal in arbitrary units and the maximum intensity of the *iso*-DBP^+^ was set as the limit of the colour bar. **b**, Comparison between the simulated scattering curves from the trajectories and the two species observed in our experiment. The averaged difference scattering curve for the simulated trajectories is shown in blue, whereas the difference scattering curves for the two species identified in the UED experiment are presented in black and red. The absence of any discernible feature in **a** and in the blue curve, calculated from the ab initio molecular dynamics trajectories and averaged over time, indicates that the photoionized DBP undergoes no immediate structural change after ionization. Source Data

Source Data Extended Data Fig. 7

**Extended Data Fig. 8:**
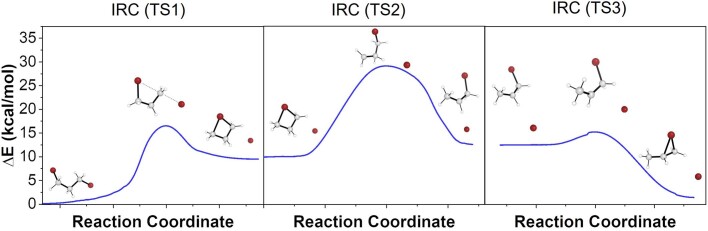
IRC calculation. The IRC calculation for the transition states TS1, TS2 and TS3 along the reaction pathway. The structures of the transition states used for the IRC calculation are shown in Supplementary Table 5. By analysing the results of the IRC calculation, it is possible to confirm that the transition state identified is indeed relevant to the reaction of interest and plays a critical role in the reaction mechanism. Source Data

Source Data Extended Data Fig. 8

**Extended Data Fig. 9:**
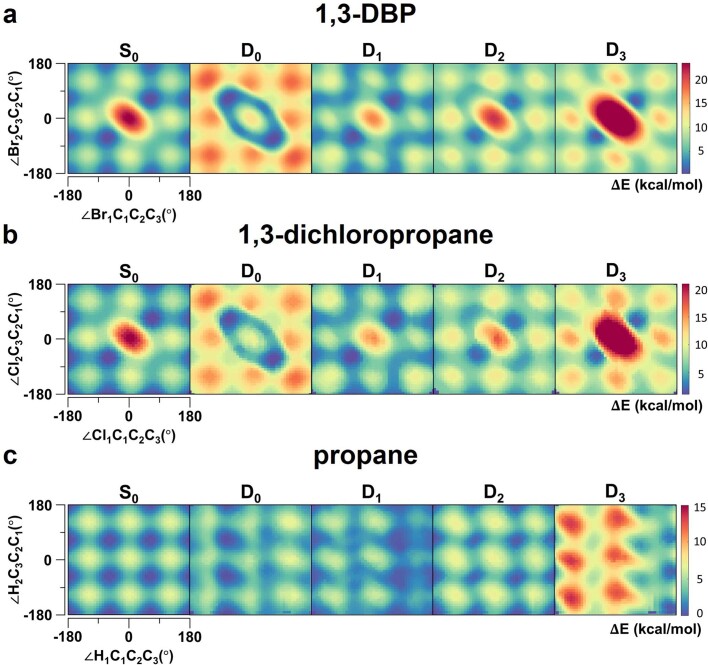
PESs of the S_0_ (neutral ground state), D_0_ (first doublet state), D_1_, D_2_ and D_3_ states of 1,3-DBP, 1,3-dichloropropane and propane. The energies here are calculated using (XMS-)CASPT2 corrections of the (SA-)CASSCF(8,8) energies. Source Data

Source Data Extended Data Fig. 9

## Figures and Tables

**Extended Data Table 1:**
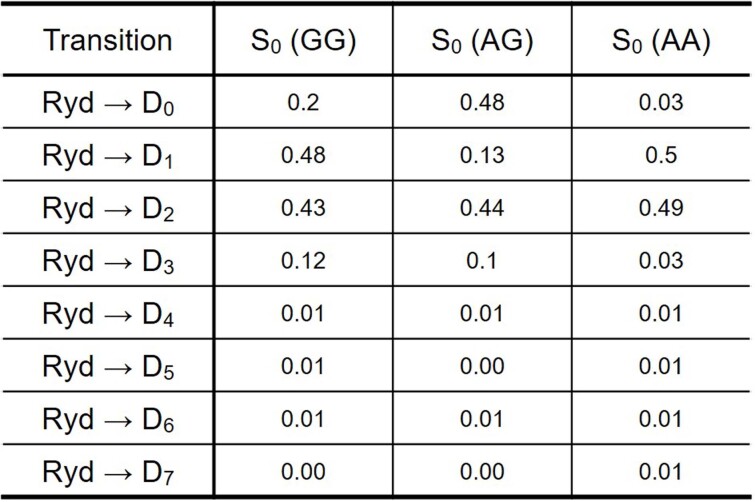
Norms of Dyson orbitals

The calculated norms of Dyson orbitals computed for the three most stable conformers of DBP by considering the first Rydberg state (Ryd) as a starting state and the first eight doublets (D_0_, D_1_, D_2_, D_3_, D_4_, D_5_, D_6_ and D_7_) as final states. The Dyson orbitals were calculated by using CAS(8,8)SCF and the def2-TZVPP basis set.

## Data Availability

All data supporting the study, excluding raw 2D diffraction data, are available as source data. Raw 2D diffraction data, the sizes of which are too large to be provided in the form of source data, can be obtained from the corresponding author on reasonable request. Source data are provided with this paper.

## Code availability

The necessary scripts to run IRC and DFT calculations using Gaussian 16, CASSCF, CASPT2, and XMS-CASPT2 calculations using Bagel code, and the ab initio molecular dynamics simulations conducted using the Newton-X package, interfaced with Gaussian 16, as well as other non-commercial analysis codes, are available from the corresponding author on reasonable request.
