# Supplementary material for: Capturing the generation and structural transformations of molecular ions
Source: Nature. 2024 Jan 10;625(7996):710–4. doi: 10.1038/s41586-023-06909-5 (PMC10808067; doi:10.1038/s41586-023-06909-5)
Supplement: Supplementary file 1 — This file contains Supplementary Methods, Supplementary Discussion and Supplementary References; it includes 18 Supplementary Figures and 8 Supplementary Tables. [file 41586_2023_6909_MOESM1_ESM.pdf]

nature portfolio

https://doi.org/10.1038/s41586-023-06909-5

Supplementary information

Capturing the generation and structural transformations of molecular ions

In the format provided by the authors and unedited

Supplementary Information

Capturing the generation and structural transformations of molecular ions

Jun Heo,[1,2,†] Doyeong Kim,[1,2,†] Alekos Segalina,[1,2] Hosung Ki,[1,2] Doo-Sik Ahn,[1,2,&] Seonggon Lee,[1,2] Jungmin Kim,[1,2] Yongjun Cha,[1,2] Kyung Won Lee,[1,2] Jie Yang,[3,‡] J. Pedro F. Nunes,[4,#] Xijie Wang,[3] Hyotcherl Ihee[1,2,*]

[1]Center for Advanced Reaction Dynamics (CARD), Institute for Basic Science (IBS), Daejeon 34141, Republic of Korea

[2]Department of Chemistry and KI for the BioCentury, Korea Advanced Institute of Science and Technology (KAIST), Daejeon 34141, Republic of Korea

[3]SLAC National Accelerator Laboratory, Menlo Park, California 94025, United States

[4]Department of Physics and Astronomy, University of Nebraska–Lincoln, Lincoln, NE, USA

[&]Present address: Foundry Business, Samsung Electronics, Republic of Korea

[‡]Present address: Center of Basic Molecular Science, Department of Chemistry, Tsinghua University, Beijing, China

[#]Present address: Diamond Light Source, Harwell Science and Innovation Campus, Didcot, OX11 0DE, United Kingdom

[†] These authors contributed equally to this work.

* Corresponding author. Email: hyotcherl.ihee@kaist.ac.kr

Table of Contents

Supplementary Methods — Pages

1. Details of data collection ······ S5 – S6
2. Details of data processing and analysis ······ S6 – S10
3. Validity of applying Legendre decomposition to account for the beam deflection artifact ······ S10 – S13
4. Power dependence of $\Delta I_0(s,t)$ signals ······ S13 – S15
5. Quantification of the induction time ······ S15
6. Singular value decomposition ······ S15 – S16
7. Details of the kinetic analysis using SVD ······ S16 – S17
8. Detailed procedure for generating theoretical static diffraction curve ······ S17 – S19
9. Static radial-distribution-function (RDF) from the experimental data ······ S19
10. Detailed procedure for generating difference signals ······ S19 – S20
11. Details of structural analysis ······ S20 – S21
12. Details of structural refinement ······ S21 – S23
13. Determination of the relative population of ions ······ S23 – S24
14. Computational details ······ S25 – S26
15. Details of potential energy scans ······ S27
16. Details of Dyson orbitals ······ S27 – S29
17. Details of surface hopping simulation ······ S29 – S30
18. Emission lifetime ······ S30

Supplementary Discussion — Pages

1. Comparison of the observed induction period with previous studies ······ S31
2. Comparison with a previous study on ions using UED ······ S31 – S32
3. Deflection of the electron beam and scattering profile due to the generation of ions ······ S32 – S33
4. Quantitative analysis of the static RDF of the ground state DBP ······ S33 – S34
5. Qualitative analysis of ΔRDF(r) of species A^+ and B^+ ······ S34

6. Comparison of the observed structure of bromonium MBP^+ with those in crystals ········ S35 – S36
7. Overall structural pathways determined from the kinetic and structural analysis S36 – S37
8. Existence of the induction period ········ S37 – S38
9. Structure of the Rydberg state ········ S38
10. Results of surface hopping simulations ········ S38 – S40
11. Reaction pathways from D_2 state to *iso*-DBP^+ ········ S40
12. Reaction pathways to form *iso*-DBP^+ and MBP^+ ········ S40 – S42
13. Loosely bound nature of *iso*-DBP^+ supported by calculated vibrational frequencies ········ S42
14. Potential generality of ionized state dynamics ········ S42

<u>References</u> Pages

1. References ········ S43 – S44

Supplementary Figures Pages

1. Supplementary Fig. 1 .... S45
2. Supplementary Fig. 2 .... S46
3. Supplementary Fig. 3 .... S47
4. Supplementary Fig. 4 .... S48
5. Supplementary Fig. 5 .... S49
6. Supplementary Fig. 6 .... S50
7. Supplementary Fig. 7 .... S51
8. Supplementary Fig. 8 .... S52
9. Supplementary Fig. 9 .... S53
10. Supplementary Fig. 10 .... S54
11. Supplementary Fig. 11 .... S55
12. Supplementary Fig. 12 .... S56
13. Supplementary Fig. 13 .... S57
14. Supplementary Fig. 14 .... S58
15. Supplementary Fig. 15 .... S59
16. Supplementary Fig. 16 .... S60
17. Supplementary Fig. 17 .... S61
18. Supplementary Fig. 18 .... S62

Supplementary Tables Pages

1. Supplementary Table 1 .... S63
2. Supplementary Table 2 .... S64
3. Supplementary Table 3 .... S65
4. Supplementary Table 4 .... S66
5. Supplementary Table 5 .... S67
6. Supplementary Table 6 .... S68
7. Supplementary Table 7 .... S69
8. Supplementary Table 8 .... S70

Supplementary Methods

Details of data collection

The data were collected using the MeV-UED facility at SLAC National Accelerator Laboratory[12,31]. The electron pulse was generated by an S-band klystron-powered radio-frequency (rf) gun, which provided an 80 MV/m accelerating field. The kinetic energy of the electron pulses is 3.7 MeV, and each pulse contains approximately 10^4 electrons, focused to a diameter of 200 μm FWHM. The sample, 1,3-dibromopropane (99%), was purchased from Sigma Aldrich and used without further purification. The gas was introduced into the vacuum chamber with a flow cell 100 μm nozzle, and the nozzle was heated to 40 °C to prevent clogging of the molecules. During operation, the chamber pressure is ~ 7 x 10^{-6} torr. The electron and laser beams were aligned to co-propagate with a 5-degree angle, intersecting the gas jet at roughly 250 μm underneath the nozzle exit. A bismuth (Bi) film was used to determine the instrument response function (IRF) of the experiment. Monitoring the time-dependent diffraction signal from a Bi film is a standard procedure at the SLAC MeV-UED facility to estimate the IRF of the experiment[31]. Specifically, the temporal behavior of the diffraction intensity of the (410) ring from a Bi(111) film with a thickness of 25 nm was monitored. The measured time-dependent diffraction intensity of the Bi film is plotted in Supplementary Fig. 2. The IRF value was determined by fitting a single-exponential response for the Bi(111) film convoluted with a Gaussian function for the IRF, using the following equation.

$$y = (f * g)(t) = \int_{-\infty}^{\infty} f(\tau) g(t-\tau) d\tau$$

$$where,\ f(t) = \begin{cases} A_1\left(1 - \exp\left(-\frac{t-t_0}{\tau_1}\right)\right),\ t \geq t_0 \\ 0,\ t < t_0 \end{cases},\ g(t) = \frac{1}{\sigma\sqrt{2\pi}} \exp\left(-\frac{1}{2}\frac{t^2}{\sigma^2}\right) \quad \text{(S1)}$$

Here, y is the fitting function for the intensity changes of the Bi(111) film, t_0 refers to time zero, τ_1 refers to the time constant of the exponential response of the Bi(111) film, A_1 refers to the amplitude of the exponential response of the Bi(111) film, and σ^2 refers to the variance of the gaussian function. The estimated FWHM of IRF, which corresponds to 22ln2σ, is 104 ± 35 fs. The IRF is depicted alongside the temporal profiles of $s\Delta I_0$ and $s\Delta I_1$ in Fig. 2d. The substantial difference in thickness between the Bi film (25 nm) and the DBP sample (100 μm) could

potentially result in a slight deviation of the determined IRF from the true IRF of the scattering experiment on the DBP sample. Nevertheless, considering previous experimental results conducted under similar conditions (where the IRF was also determined using Bi film experiments and reported to be around 150 fs), successfully capturing dynamics on the order of 200 fs, it is reasonable to deduce that the actual IRF of the gas-phase experiment does not significantly deviate from the IRF measured using the Bi film[12,42]. Hence, the IRF of our gas-phase UED experiment is expected to be approximately 104 fs, similar to the value determined with the Bi film. The gas jet had a size of around 300 μm FWHM with a number density on the order of 10^{16} cm^{-3} at the interaction region. The system was operated at a repetition rate of 120 Hz. The UED data were measured at various time delays in the range - 5 ps to 5 ps with a time step of 1 ps, 10, 15, 20, 25, 35, 40, 50, 60, 100, 200, and 300 ps yielding a total of 22 time delays. Diffraction patterns at each time delay were accumulated for 20 minutes. For photoexcitation, a 565 μJ pump laser pulse with a center wavelength of 267 nm and an 1.3 nm FWHM bandwidth, was linearly polarized and elliptically focused to a spot with the size of 280 × 200 $μm^2$, giving a fluence of 1200 mJ/cm^2. The duration of the pump UV pulse was estimated to be 80 fs FWHM. The electron detector comprised a P43 phosphor screen with dimensions of 40 × 40 mm^2, positioned perpendicular to the beam path and featuring a center hole, a 45° mirror with a center hole, an imaging lens, and an Andor iXon Ultra 888 electron-multiplying charge-coupled device camera with 1024 × 1024 pixels. The phosphor screen has a thickness of 100 μm and a grain size of 10–20 μm, chosen for maximum efficiency. A 40-mm f/0.85 lens was used to capture images of the phosphor screen. In this configuration, each pixel corresponds to an area of 39 × 39 $μm^2$ on the screen, and the detector exhibits a point-spread function with a root-mean-square (rms) value of 85 μm.

Details of data processing and analysis

The scattering patterns (Extended Data Fig. 1) are highly asymmetric and exhibit significant positional shifts of the undiffracted electron beam unlike typical ones observed in UED data from neutral intermediates. The asymmetry arises from the e-beam deflection caused by the Coulomb interaction with the ion product. The asymmetric scattering image provides evidence that the ion is generated by photoreaction, but at the same time, it acts as an artifact that mixes

unwanted patterns into the diffraction signal. As this artifact generated by the beam deflection can distort the structural information of the molecule[20], it is a hindering factor in extracting the structural information of transient species from the diffraction pattern analysis. To properly analyze the asymmetric scattering patterns, we had to employ a method capable of removing the asymmetric scattering signal. To achieve a detailed and quantitative analysis of the molecular structural changes upon photoionization, it is necessary to separate the difference scattering signal into two components: one for the scattering signal from the molecular structural changes and another for the artifact signal due to the ionic-species induced beam deflection[43]. The deflection artifact causes a change in the signal when the main electron beam moves in one direction after the interaction region. This results in a scattering pattern change resembling a cosθ shape in the low-s region. To remove the artifact corresponding to cosθ in the low-*s* region, we introduced a method that removes the existing anisotropic scattering signal based on symmetry[35,36,44].

Before the correction, contributions from the hole in the detector (which allows the undiffracted electrons to pass through) and other detector artifacts (such as phosphor screen afterglow or optical pump background) were removed using a mask. The center of each diffraction pattern was determined independently by fitting a circle to the concentric intensity isoline. We converted the obtained diffraction pattern to a 1-D diffraction curve through the following steps. First, we subtracted the 2D diffraction pattern at a negative time delay signal from that at a positive time delay signal to obtain a 2D difference pattern. The 2D difference patterns at each time delay were then averaged to produce a single image.

To remove the beam shift effect caused by the formation of cations and eliminate the high-*s* side artifact, we utilized Legendre decomposition, a decomposition based on the Legendre series. The difference scattering image was decomposed using Legendre decomposition into a linear combination of two components with scattering intensity distributions of 0th and 1st order Legendre polynomials along the φ angle, the 0th order term (ΔI_0) and 1st order term (ΔI_1), respectively. The relationship between the original difference scattering image and the three terms obtained from the decomposition is expressed by the following equation.

$$\Delta \mathrm{I}(s,\varphi,t) \propto \Delta \mathrm{I}_0(s,t) + \mathrm{P}_1(\cos\varphi)\Delta \mathrm{I}_1(\mathrm{s},\mathrm{t}) \quad \text{(S2)}$$

In this equation, s is the momentum transfer vector, P_1 is the 1st-order Legendre polynomial, and φ is the azimuthal angle on the CCD plane. The momentum transfer vector s is defined as follows.

$$s = \frac{4\pi}{\lambda}\sin(\frac{\theta}{2}) \quad \text{(S3)}$$

where λ is the de Broglie wavelength of the incident electrons and θ is the angle between the incident and scattered electrons. The conversion between detector pixels and momentum transfer was calibrated using the Bragg peak positions of a known single-crystal silica target.

Our assumption is that the resulting ΔI_0 term would represent an isotropic scattering signal from the molecular structural changes and ΔI_1 term would represent the artifact signal due to the ionic-species induced beam deflection. Based on this, we analyzed the obtained ΔI_0 term to retrieve the molecular structures of the reactants and the transient species. The resulting ΔI_0 and ΔI_1 terms extracted from the measured scattering images are shown in Figs. 2b and 2c, respectively. Here we note that as we used a linearly polarized laser to excite the reactant molecules (here, DBP molecules), an orientation-selective excitation of the reactant molecules may occur. In other words, there is a possibility that the photoexcitation occurs with different probabilities depending on the angular orientation of the DBP molecule with respect to the direction of the polarization of the excitation laser. Such a photoselective excitation induces orientational distributions of reactants, intermediates, and products that should be manifested in the anisotropy in the scattering patterns. More specifically, a photoselective excitation yields an additional, anisotropic component (ΔI_{2n}) in the scattering pattern with intensity distributions of 2nd or higher order Legendre polynomials along the φ angle[45]. Considering the potential contribution of the photoselective orientation, ΔI_{2n} terms, we also decomposed the experimental images into a sum of higher-order components. The ΔI_{2n} terms obtained from the decomposition not only did not show significant signals but also deteriorated the ΔI_0 and ΔI_1 terms due to the overfitting. This result reveals that the contribution of the ΔI_{2n} term is negligible and it suffices to decompose the experimental data into only two terms, ΔI_0 and ΔI_1.

Since the process of ionization itself arises from the interaction with the linearized pump pulse, it is expected that an anisotropic feature would persist after ionization. This consideration indicates that although the ionization process itself should be anisotropic due to

photo-selectivity, the absence of an anisotropic signal implies that it may lose anisotropy either during the ionization process or after being converted into another species. Besides, there may be reasons why anisotropic features are not observed in the experimental data, apart from the beam shift due to Coulomb interaction. Firstly, if structural changes are not accompanied immediately after ionization, the orientation of selectively excited molecules may not appear in the scattering pattern. This can be inferred from the absence of a difference signal in $\Delta I_0(s,t)$ during the induction period, as seen in Fig. 2b and 2d, which show that no structural changes are observed until 3.6 ps after ionization. Secondly, if the species generated during ionization loses anisotropy quickly, anisotropy signals may not be observed. The long induction period of 3.6 ps can be the main reason for the absence of the signal in $\Delta I_{2n}(s,t)$. During the induction period, ionized molecules lose anisotropy through rotational dephasing, and 3.6 ps is a sufficient time for such loss to occur[46,47]. Thus, the absence of an anisotropy signal can be attributed to the lack of structural changes during the induction period and fast dephasing of the ion in vacuum.

If (i) the laser beam is significantly larger than the electron beam and ideally has a flat-top profile, (ii) the laser and electron beam profiles are perfectly symmetric, and (iii) they are perfectly aligned, the deflection of the electron beam caused by its Coulomb interaction with the generated ions would be minimized. However, practical considerations to ensure a sufficient fluence to induce REMPI led us to use a laser beam with a diameter of approximately $280 \times 200\ \mu m^2$, comparable to the dimensions of the electron beam. In addition, the alignment between the laser and electron beams was not perfect. Despite these factors, the conditions of the laser and electron beams employed in our experiment—while not optimized for minimizing the electron beam deflection—turned out to be well-suited for observing ion generation and structural changes via UED, given the constraints of our experimental setup.

The extent of electron beam deflection due to the generated ions was substantial enough to enable us for precise tracking of the electron beam displacement caused by Coulomb interaction, thereby facilitating accurate timing of ion generation events. Simultaneously, the extent of deflection was limited enough to prevent significant distortion of the diffraction pattern—except that it is off-centered as the electron beam is deflected—ensuring that the structure information encrypted in the diffraction pattern remained largely unaffected. Despite the imperfect alignment and profiles of the laser and electron beams, these conditions enabled

the use of Eq. S2 to describe the observed asymmetric difference scattering patterns, facilitating the extraction of both asymmetric and isotropic components using Legendre decomposition.

Validity of applying Legendre decomposition to account for the beam deflection artifact

As explained in the "Data processing and analysis" section, we applied Legendre decomposition (Eq. S2) to separate the experimental difference scattering pattern into two components: one containing structural information and the other representing the deflection artifact. The fundamental assumption underlying this approach is that the structural information resides within the isotropic component (ΔI_0 term), while the deflection artifact induced by ions is encapsulated within the asymmetric term (ΔI_1 term). In this section, we discuss the robustness of this approach through simulations.

The application of Legendre polynomials for disentangling deflection artifact contributions is not a novel method exclusive to our work. Other research groups have previously utilized Legendre projection to eliminate asymmetric components, retaining only the isotropic component (ΔI_0 term)[20,36]. While Legendre projection has been used for eliminating the asymmetric components, our approach employed Legendre decomposition, a closely related method, to isolate one of the asymmetric components (ΔI_1) along with the isotropic component (ΔI_0).

In the simulation, we assumed equal deflection extents for both the direct and diffracted beams. Based on this assumption, we generated mock difference scattering patterns that incorporated deflection artifacts by calculating the difference between two static patterns: one representing the scattering pattern at the reference time delay (before laser excitation) and the other depicting the deflected scattering pattern at a positive time delay (after laser excitation). Notably, the experimentally observed difference scattering patterns display distinct bipolar characteristics at positive time delays, with one side of the pattern exhibiting pronounced positive intensities and the opposite side showing significant negative intensities (Fig. 2 and Extended Data Fig. 1). To simulate the scattering pattern at the reference time delay, we calculated the scattering pattern corresponding to the molecular structure at negative time delays, ensuring that the center of the scattering pattern was aligned with the center of the detector (ground-state scattering signal). For the pattern at the positive time delay, we

calculated the scattering pattern corresponding to the ion structure at positive time delays, while intentionally shifting the center of the scattering pattern to a different position (center-shifted excited-state scattering signal). Specifically, we shifted the center of the scattering pattern in a specific direction (in this case, upwards). By calculating the difference between the center-shifted excited-state scattering signal and ground-state scattering signal, we were able to effectively mimic the bipolar features observed in the experimentally obtained difference scattering patterns (Supplementary Fig. 4). With varying the extent of beam deflection, as indicated by the number of pixel shifts, we generated mock difference scattering patterns. Subsequently, we applied Legendre decomposition to the generated mock difference scattering images to assess the extent to which the resulting ΔI_0 term matches the azimuthally integrated curve of the original difference scattering pattern. The results are illustrated in Supplementary Fig. 4.

The degree of electron beam deflection under our experimental conditions is clearly depicted in Extended Data Fig. 2, showing a range of three to four pixels at maximum. According to the simulation results, Legendre decomposition remains valid for deflection extents of 4 pixels or less. These findings validate the use of Legendre decomposition as a valid and effective approach for mitigating the deflection artifact present in our difference scattering patterns. The simulation took into account the effect of the beam deflection, but the effect of the beam broadening was not included. In the second simulation, we explored the effect of the beam broadening with the beam deflection at 3 pixels. To take into account the effect of beam broadening, we convoluted a 2D gaussian function to the center shifted excited-state scattering signal, varying the FWHM of the gaussian function. The remaining procedures are identical to those of the first simulation. According to the simulation results illustrated in Supplementary Fig. 5, Legendre decomposition remains valid for broadening extents of even up to 6 pixels.

In this work, we utilized Legendre decomposition to disentangle the isotropic signal caused by structural changes and asymmetric signal induced by beam deflection. The fundamental assumption underlying this approach is that the signal containing structural information resides within the isotropic component (ΔI_0), while the deflection artifact induced by ions is encapsulated within the anisotropic component (ΔI_1). In other words, the validity of applying Legendre decomposition can be guaranteed when the artifact signal induced by beam deflection exhibits a cosine-shaped distribution along the azimuthal angle. In this context, the

azimuthal angle refers to the angle between a directional vector on the detector plane from the beam center to the detector pixel and the direction of beam shift on the detector plane. Recognizing that this assumption may not hold entirely true, we demonstrated the effectiveness and acceptability of Legendre decomposition through the simulations, particularly when the degree of deflection is relatively moderate.

It is worth noting that under conditions of substantial beam deflection, the resulting artifact signal significantly deviates from a cosine-shaped distribution along the azimuthal angle. To offer a more accurate description of the beam deflection artifact in cases of pronounced deflection, the incorporation of additional terms related to higher orders of Legendre polynomials, such as ΔI_2, ΔI_3, and ΔI_4, presents a viable approach. In our data, the extent of beam deflection was not significant, allowing ΔI_1 alone to effectively describe the beam deflection artifact. We refrained from introducing higher-order terms due to the potential risk of overfitting the experimental noise present in our data.

To ensure that the shift of the electron beam has a minimal influence on both the shape of the ΔI_0 curves and the structural parameters derived from the analysis of ΔI_0, we conducted further analyses. Specifically, we applied shifts of 3 pixels and 6 pixels to the experimental 2D scattering patterns, resulting in shifted images. From these shifted images, we extracted the ΔI_0 curves and performed structural refinement. The resulting structural parameters were then compared with those obtained from the analysis of unshifted images. The comparisons are presented in Supplementary Table 2.

The comparison of results shows that the structural refinement results are minimally affected by the pixel shifts, even in the case of a 6-pixel shift. This observation can be attributed to the fact that, in the high-*s* region ($s > 3.0$ Å^{-1}), the oscillation frequency of the ΔI_0 curves remains largely unchanged, even when the electron beam shifts are introduced. Instead, it appears that the changes in the curves primarily involve an upward shift of the baseline. The oscillatory frequency of the curve, which contains crucial structural information, appears to be reasonably preserved despite significant pixel shifts, resulting in relatively minor alterations in the structural parameters. Notably, for the most crucial structural parameter, the Br–Br distance, we observe only a negligible error within 0.03 Å. Relative distances between lighter atoms,

such as C–C distances, display more noticeable but still modest errors, around 0.1 Å. The bond angles also maintain their general trends with minor errors. In contrast, the relative fraction of ground state DBP conformers shows significant variation. Specifically, in the case of a 6-pixel shift, the fraction of the AA conformer increases by more than twofold compared to the fraction derived from the unshifted images. This highlights the high sensitivity of the relative fraction of conformers to variations in the low-s region of the curve. The high sensitivity of the relative conformer fractions to the low-s region of the ΔI_0 curves can be attributed to the significant differences in the shape of the scattering curves for GG, AG, and AA conformers in this region. Nonetheless, a 3-pixel shift induces only modest changes in the relative fractions: GG remains the predominant component, while AG and AA fractions are similar.

Power dependence of $\Delta I_0(s,t)$ signals

To verify if the observed signals were indeed linked to the [2+1] REMPI process, we examined the relationship between the power of the pump pulse and the magnitude of the observed $\Delta I_0(s,t)$ signals. This analysis aimed to establish whether the signals required three photons, thus indirectly supporting the hypothesis that the observed signals can be attributable to the [2+1] REMPI process. This analysis involved measuring the sum of the signal intensities ($\sum_s s\Delta I_0(s,t)$) from the transient species while systematically varying the pump pulse power. Our study focused on two distinct s regions: 1) a narrower region with s values ranging from 3.4 to 4.0 Å^{-1}, and 2) a broader region spanning s values from 1.3 to 8.0 Å^{-1}. We selected these regions to address specific considerations in our analysis. The choice of the narrower s region was motivated by its relatively higher signal-to-noise ratio (SNR), which ensured the precision of our results. Notably, the low-s region (1–2 Å^{-1}) is susceptible to shot-to-shot fluctuations in the electron beam, leading to a lower SNR. Conversely, in the high-s region (> 4 Å^{-1}), we encountered weaker signals. In addition to our investigation of the narrower s region, we extended our analysis to encompass a broader s range, specifically s = 1.3–8.0 Å^{-1}, to address any potential concerns associated with the choice of a narrower range. The results, as illustrated in Supplementary Fig. 3, show a clear correlation between the intensity of the difference scattering pattern at 45 ps and the laser fluence for both s ranges. Specifically, the sum of the signal intensities demonstrated a proportionality to the power of approximately 2.7 ± 0.1 and

2.8 ± 0.9 for the narrower and broader *s* regions, respectively. Importantly, this observation lends strong support to the conclusion that the UED signal indeed originates from reaction intermediates formed via a 3-photon process[22].

Concerning the 3-photon process, there exist two viable pathways for achieving photoionization: [2+1] REMPI and non-resonant three-photon ionization. REMPI, as an ionization technique, is renowned for its exceptional selectivity and high ionization efficiency, as noted in various studies[48-50]. For example, an ionization efficiency of 10% was reported[21]. An important feature of REMPI is its ability to form ions with minimal fragmentation, making it valuable for ion sources[51,52]. In REMPI, resonant absorption of one or multiple photons leads to an intermediate electronic state, followed by an additional absorption of another photon that ionizes the atom or molecule[53]. These resonant interactions effectively serve as a distinctive excitation pathway, imparting REMPI with its distinctive selectivity. Specifically, the [2+1] REMPI process investigated in this study involves a two-photon absorption event that leads to a Rydberg state as an intermediate state, followed by an additional single photon absorption to exceed the ionization threshold[21,54]. Overall, this process involves a three-photon interaction leading to ionization. While we cannot entirely rule out the possibility of a non-resonant three-photon ionization scenario, we suggest that the observed reactions are based on the [2+1] REMPI process for the following reason. In our work, we employed a substantially lower fluence in comparison to a previous study where significant contributions from both REMPI and non-resonant ionization were identified[24]. Given that the preference for REMPI over non-resonant ionization increases as fluence decreases, we propose that in our experimental conditions, with a fluence approximately 5–7.5 times smaller than that used in the previous experiment[24], REMPI would dominate. Furthermore, the primary focus of our study does not revolve around the relative ratio between non-resonant and resonant ionization products. Whether the ionization occurred via resonant or non-resonant processes is inconsequential and lies beyond the scope of our investigation if the dominant portion of DBP^+ exists in an excited ionic state. Despite the use of a significantly more intense laser in the prior study[24], their experimental data reveals that the majority of DBP^+ ions are populated in an excited ionic state rather than the ground state (D_0), which is consistent with our findings and assignment.

Nevertheless, we note that our results do not completely rule out the potential contribution of non-resonant three-photon ionization, rather than REMPI. While we provided

theoretical support that the induction period we observed could be expected if DBP molecules were ionized via the REMPI process, we did not explore the expectations for different phenomena that could arise from non-resonant three-photon ionization.

Quantification of the induction time

To quantify the induction time, we analyzed the temporal behavior of $\Delta I_0(s,t)$ and $\Delta I_1(s,t)$ and calculated the area under the curve (AUC) for each data set. This enabled us to show the rise of signal over time and compare the resulting time trends. Photoinduced ionization is known to occur rapidly (< 40 fs)[23], so the artifact signal corresponding to ion generation, $\Delta I_1(s,t)$, should also show an instantaneous rise near time zero. To confirm the hypothesis, we examined whether $\Delta I_1(s,t)$ accurately described the beamshift effect caused by ion generation, and found that the trend in the intensity change in $\Delta I_1(s,t)$ matched that of the beamshift (Extended Data Fig. 2). Therefore, by accurately determining the point at which the signal intensity of $\Delta I_1(s,t)$ begins to rise, we can confirm the actual time zero in the experiment. The results showed that the deflection of the beam actually occurs near the time zero we assigned, and the signal intensity of $\Delta I_0(s,t)$, which corresponds to the structural changes, rises ~4 ps later than the beamshift (Fig. 2d).

Singular value decomposition

To investigate the time-dependent features in the experimental data of DBP, we applied the singular value decomposition (SVD) analysis to the measured isotropic difference scattering curves (ΔI_0). For the SVD analysis, we built an $n_s \times n_t$ data matrix, $\mathbf{A}$, whose column vectors are experimental time-resolved difference scattering curves, where n_s is the number of s points in the difference scattering curves and n_t is the number of time-delay points. As a result of SVD, the matrix $\mathbf{A}$ is decomposed into three matrices satisfying the relationship of $\mathbf{A} = \mathbf{USV}^T$. $\mathbf{U}$ is an $n_q \times n_t$ matrix whose column vectors are called left singular vectors (LSVs) of $\mathbf{A}$, $\mathbf{V}$ is an $n_t \times n_t$ matrix whose column vectors are called right singular vectors (RSVs) of $\mathbf{A}$, and $\mathbf{S}$ is a diagonal $n_t \times n_t$ matrix whose diagonal elements are called singular values of $\mathbf{A}$. The matrices

$\mathbf{U}$ and $\mathbf{V}$ follow the relationships of $\mathbf{U}^T\mathbf{U} = \mathbf{I}_{nt}$ and $\mathbf{V}^T\mathbf{V} = \mathbf{I}_{nt}$, respectively, where $\mathbf{I}_{nt}$ is an identity matrix. LSVs represent time-independent s-spectra, the RSVs represent time-dependent amplitude changes of the corresponding LSVs, and the singular values represent the weights of the corresponding LSVs and RSVs. Since the singular values are ordered so that $a_1 \geq a_2 \geq \cdots \geq a_n \geq 0$, (both left and right) singular vectors on more left are supposed to have larger contributions to the experimental data matrix $\mathbf{A}$. The results of SVD of the isotropic scattering signals, $s\Delta I_0(s,t)$, are summarized in Extended Data Fig. 3. We used the SVD results to construct the proper kinetic model for the scattering data. Because SVD extracts the feature and time-dependent character of the scattering components, we can quantify the main contributors to the dynamics and the corresponding time constants.

Details of the kinetic analysis using SVD

To extract kinetics information of intermediates and their structures from $\Delta I(s,t)$, we followed the well-established procedure, which had been applied to time-resolved X-ray liquidography (TRXL) studies on small molecules, consisting of kinetic analysis using singular value decomposition (SVD). First, SVD, which is a factorization method to separate the time-dependent information from the time-independent information, was performed on the $\Delta I(s,t)$ matrix for the entire time points of -5 ps - 300 ps and the s range of 1.3–8.0 Å^{-1}, yielding left singular vectors (LSVs), right singular vectors (RSVs) and singular values. The LSVs, RSVs and singular values contain time-independent information, their time dependence, and their relative significance, respectively.

We obtained clues about the number of intermediates associated with temporal dynamics by performing the SVD analysis on the whole data. The LSVs from the SVD analysis are shown in Extended Data Fig. 3a and 3b. Based on the singular value, which indicates the dominance of each component among the data, and autocorrelation values in Extended Data Fig. 3c, the first two components are dominant among the data showing the high autocorrelation values of RSVs and LSVs. In the first two curves in Extended Data Fig. 3a and 3b, the SVD results of two main components are shown. To analyze the temporal behaviors of the two components, we fitted the RSV features of these two components with exponential functions and one induction delay constant. The exponential fitting results are shown in

Extended Data Fig. 3d. Two major components are fitted with the one induction delay (t_d = 3.6 ± 0.3 ps) and two exponential functions (t_1 = 15 ± 2 ps, t_2 = 77 ± 15 ps).

Then, we conducted kinetics-constrained analysis (KCA), which is a method for generating theoretical difference scattering curves using time-dependent population changes of the intermediates expressed with a set of variable kinetic parameters, with an assumed candidate kinetic model from the results of SVD on the data matrix[25,26]. In the KCA, we fixed the kinetic parameters as the time constants extracted from the SVD results and minimized the difference between the experimental difference scattering curves and the theoretical difference scattering curves. This optimization process contains the refining of the species-associated difference scattering curves (SADSs) of the corresponding species. For constructing possible models, we assumed the "structural dark state", D, to describe the invisible dynamics in the first 3.6 ps. Thus, the possible kinetics models are constructed with two components (species A^+ and B^+) with two time constants (15 ps and 77 ps), after the induction period of 3.6 ps.

Through KCA, we determined the optimal kinetic model among all possible models, which are shown in Extended Data Figs. 4a and 4b. There are a total of two kinetic models that satisfy the conditions of having three kinetic components (two decay constants and one induction time constant) and two kinetic species. The first one is a sequential model in which the first species (species A^+) is formed from the dark state, followed by its conversion to the second species (species B^+), while the other is a parallel model in which two species are generated from the dark state simultaneously (Extended Data Fig. 4). The difference scattering curves generated by applying the kinetic models were compared to the experimental $\Delta I_0(s,t)$, and their differences (χ^2) were minimized through fitting. The KCA results of both models are shown in Extended Data Fig. 4c and 4d. The sequential model showed a 64.2% lower χ^2 value than the parallel model, meaning that the sequential model better explains the kinetics observed in the experimental data. Fig. 3a shows the diagram of the final determined sequential model, and Fig. 3b shows the population dynamics of all species involved in the reaction.

Detailed procedure for generating theoretical static diffraction curve

Theoretical scattering curves used for the structural analysis were calculated under the independent atom model (IAM). In this model, the static diffraction curve, I(s), can be expressed as the sum of the interference terms for all atomic pairs, the molecular scattering ($I_{mol}(s)$) and the sum of the atomic scattering, $I_{at}(s)$, for each atom in the molecule.

$$\mathrm{I}(s) = \mathrm{I}_{\mathrm{mol}}(s) + \mathrm{I}_{\mathrm{at}}(s) \quad \text{(S4)}$$

The atomic scattering term solely contains the atomic information without any structural information and can be calculated by using the empirical formula of the target molecule, regardless of its shape.

$$\mathrm{I}_{\mathrm{at}}(s) = \sum_{i=1}^{N} f_i^*(s) f_i(s) \quad \text{(S5)}$$

In Eq. S5, $f_i(s)$ is the scattering amplitude of the ith atom as calculated using the Mott-Bethe formula[55]. The molecular scattering term contains information on the interatomic distance of all atomic pairs in the molecule. In the case of an isotropic system with N atoms, $I_{mol}(s)$ can be expressed as the following Debye equation.

$$\mathrm{I}_{\mathrm{mol}}(s) = \sum_{i=1}^{N} \sum_{j=1,\ j\neq i}^{N} f_i^*(s) f_j(s) \frac{\sin(s r_{ij})}{s r_{ij}} \quad \text{(S6)}$$

In this equation, r_{ij} is the distance between the i_{th} and the j_{th} atom. There is a rapid drop in scattering intensity with increasing s due to the s^{-2} scaling in the elastic scattering amplitude f for each atom and the s^{-1} term in Eq. S6. To compensate for such a rapid drop, diffraction data are typically presented as modified scattering intensities, sM(s).

$$\mathrm{sM}(s) = \frac{\mathrm{I}_{\mathrm{mol}}(s)}{\mathrm{I}_{\mathrm{at}}(s)} s \quad \text{(S7)}$$

Here, sM(s) curves enhance oscillations from the $\sin(s r_{ij})/r_{ij}$ term in Eq. S6, making the comparison between experimental and simulated signals easier. Moreover, sM(s) curves can be decomposed into a radial distribution function (RDF) of the constituent interatomic distances using the Fourier sine transformation:

$$RDF(r) = \int_0^{s_{max}} s\mathrm{M}(s)\sin(sr_{ij})e^{-\alpha s^2}ds \quad \text{(S8)}$$

Here, r is the interatomic distance, $s_{\max}$ is the maximum detectable momentum transfer in the diffraction pattern, and α is the damping factor used to minimize artifacts caused by the finite nature of the data and edge effects. For the steady-state RDFs, we used $\alpha = 0.1$.

Static radial-distribution-function (RDF) from the experimental data

The experimental RDF can be obtained from the experimental $s\mathrm{M}(s)$, $s\mathrm{M}_{\mathrm{exp}}(s)$, using Eq. S8. The $s\mathrm{M}_{\mathrm{exp}}(s)$ cannot be calculated directly using Eq. S7 because $\mathrm{I}_{\mathrm{mol}}(s)$ and $\mathrm{I}_{\mathrm{at}}(s)$ cannot be separately measured via an experiment. Instead, a previously reported method[56] can be adapted, where $s\mathrm{M}_{\mathrm{exp}}(s)$ is expressed as the following equation.

$$s\mathrm{M}_{exp}(s) = \frac{\mathrm{I}_{\mathrm{exp}}(s) - \mathrm{I}_{\mathrm{bkg}}(s)}{\mathrm{I}_{\mathrm{at}}(s)}s \quad \text{(S9)}$$

$\mathrm{I}_{\mathrm{exp}}(s)$ is the experimental scattering intensity and $\mathrm{I}_{\mathrm{bkg}}(s)$ is a smooth experimental background response that includes atomic scattering contributions and instrument-specific background. $\mathrm{I}_{\mathrm{bkg}}(s)$ is approximated by fitting a sum of exponents through s values corresponding to the zero-crossing of the simulated steady-state $\mathrm{I}_{\mathrm{mol}}(s)$[57].

Detailed procedure for generating difference signals

We employ the difference-diffraction method[37] to calculate the difference signal, sometimes referred to as $s\Delta\mathrm{I}(s)$, and difference $s\mathrm{M}(s,t)$ and $\mathrm{RDF}(r,t)$, $\Delta s\mathrm{M}(s,t)$ and $\Delta\mathrm{RDF}(r,t)$, respectively. This method eliminates the background and artifacts of the diffraction signal by subtracting a reference signal recorded at a delay time $t < 0$ from the diffraction signal at a delay time, t. The $s\Delta\mathrm{I}(s)$, which is used in the structural fitting process, is defined as follows.

$$s\Delta\mathrm{I}(s,t) = s\,(\mathrm{I}(s,t) - \mathrm{I}(s,t<0)) \quad \text{(S10)}$$

where I(s,t) is the diffraction intensity recorded at pump-probe delay time, t, and I(s,t<0) is the reference signal taken at the delay time $t < 0$. A low-order polynomial background is fitted to and subtracted from the ΔI signal in order to remove any background unaccounted for by the difference-diffraction method. The time-dependent difference radial distribution functions, ΔRDF(r,t), shown in the main text were calculated as follows.

$$\Delta\mathrm{RDF}(r,t) = \int_0^{s_{max}} \Delta s\mathrm{M}(s,t)\sin(sr)e^{-\alpha s^2}ds \qquad \text{(S11)}$$

where ΔsM(s,t) is the time-dependent difference-modified scattering curve, defined as follows.

$$\Delta s\mathrm{M}(s,t) = \frac{\Delta\mathrm{I}(s,t)}{\mathrm{I_{at}}}s = \frac{\mathrm{I}(s,t)-\mathrm{I}(s,t<0)}{\mathrm{I_{at}}}s \qquad \text{(S12)}$$

For the ΔRDFs, we used $\alpha = 0.1$ to remove the high-s noise. The experimental ΔRDF was obtained by replacing the missing low-angle data, sΔI (s<1.3,t), with the theoretically fitted results using optimized structures Omitting this s range induces unphysical artifacts in the RDF, e.g. considerable negative RDF amplitudes outside the bond distances of DBP. We, therefore, fill the experimental signal in the range 0 $Å^{-1} < s <$ 1.3 $Å^{-1}$ with scaled values from the simulation. The procedure removes the artifacts but otherwise does not considerably change the RDF shape[12].

Details of structural analysis

Based on the static analysis of the ground-state DBP, we extracted structural information on species A^+ and B^+ by quantitatively analyzing their SADS(s). Before the structural refinement, we first simply compared the SADS curves with the scattering curves of candidate species calculated using the structures optimized via DFT calculations, without altering the structures. For species A^+, we tested two cationic DBP structures, *iso*-DBP^+ and 1,3-DBP^+, and one cationic MBP structure, 4-mem MBP^+, i.e., MBP^+ with a 4-membered ring, to consider various forms of structures. For the species B^+, as MBP^+ was expected to be generated (based on the ΔRDF(r) analysis), we tested three cationic MBP structures, bromonium MBP^+, 4-mem MBP^+, and 1-MBP^+ (see the section "Computational details" in SI for the details on the DFT

calculations for investigating the potential candidate species). The comparison shows that *iso*-DBP$^+$ and bromonium MBP$^+$ give the best agreements with the SADS(s) of A$^+$ and B$^+$, respectively (not shown).

Starting from the DFT structures for the candidates, we refined the structures via structural refinement. For the structural refinement, we used a global fitting approach to simultaneously fit the experimentally measured SADS(s) for species A$^+$ and B$^+$. This approach involved sharing (i) the molecular structures of the three conformations in the ground state, as well as (ii) the relative population fractions between these conformations. In the structural refinement process, we optimized (i) the relative population fractions of three conformers in the ground state, (ii) the molecular structure of the transient ionic species, so that the theoretically simulated curves agree well with the experimental SADS(s) through an iterative fitting analysis to minimize χ^2. The fit results are shown in Extended Data Fig. 6.

Photoreaction intermediates often possess significant thermal energy, which leads to broadened interatomic distance distributions due to vibrational motion. Such broadened distributions manifest in scattering signals. To explore the possibility of the involvement of "hot intermediates" with excessive thermal energy along the reaction pathway, we introduced the Debye-Waller factor into Eq. S6. The modified equation is as follows:

$$\mathrm{I}_{\mathrm{mol,DW}}(s) = \mathrm{I}_{\mathrm{mol}}(s) \times \mathrm{e}^{(s^2\sigma^2/2)} \quad \text{(S13)}$$

where $\mathrm{I}_{mol,\mathrm{DW}}(s)$ represents the molecular scattering calculated by considering the broadened interatomic distance distributions caused by vibrational motion. The exponential term in the right-hand side of the equation refers to the Debye-Waller factor, and σ^2 denotes the mean-squared displacement of interatomic distances. When Eq. S13 was applied for structural analysis with σ as an additional fitting parameter, σ converged to 0. This observation suggests that our data does not provide any evidence supporting the broadening of interatomic distance distributions in the intermediates. In other words, the intermediates do not possess excessive thermal energy.

Details of structural refinement

The difference scattering curves can be described by the function of atomic pair distances. For the structural fitting analysis, we used several structural parameters (Supplementary Table 1). To quantify the agreement between the calculated and experimental difference scattering curves, we calculated the χ^2 value using the following equation.

$$\chi^2 = \sum_{t_{min}}^{t_{max}} \sum_{s_{min}}^{s_{max}} \left(\frac{\Delta \mathrm{I}_{\mathrm{exp}}(s,t) - \Delta \mathrm{I}_{\mathrm{theory}}(s,t)}{\sigma_{exp}(s,t)} \right)^2 \quad \text{(S14)}$$

In the equation, ΔI_{exp} and ΔI_{theory} stand for the experimentally measured (isotropic signal, ΔI_0) and theoretically calculated difference scattering signals, and σ_{exp} is a standard error of ΔI_{exp}. σ_{exp} is one standard-deviation of the mean of the measured signals. The minimization of the χ^2 was performed to refine the molecular structure using the MINUIT package written at CERN, and the error analysis was performed by MINOS, a built-in algorithm in the MINUIT software[58]. During structural fitting analysis, the structural parameters were optimized by minimizing the χ^2 value. Fitted results are shown in Extended Data Fig. 5. After fitting the structural refinement process, we convert the experimentally and theoretically acquired curves to ΔRDF(r,t).

To ensure the validity of the obtained structures as global minima within the entire conformational space, we performed grid scans and molecular dynamics (MD) simulations. In this grid scan approach, we fully explored the conformational space by systematically varying all structural parameters (seven for the first intermediate and four for the late intermediate) considered in the structure refinement process, covering a broad range with regular intervals. This systematic exploration ensured a comprehensive and evenly distributed sampling of the conformational space. For each sampled structure in the grid scan, we computed the corresponding theoretical difference scattering curve and compared it to the experimental data. These comparisons allowed us to investigate the distribution of χ^2 values across the conformational space.

For each pair of structural parameters, the χ^2 values can be plotted as a function of the respective two parameters. The resulting distributions of χ^2 values for species A^+ and B^+ are shown in Supplementary Figs. 8 and 9, respectively. With seven structural parameters used for the refinement of species A^+ and four for species B^+, there are 21 and 6 pairs of parameters,

respectively, for each species. For species A^+, we present results for 6 significant parameter pairs out of the 21 pairs, omitting the rest to maintain clarity. In Supplementary Figs. 8 and 9, we denoted structural parameters displaying χ^2 deviations of one and four from the minimum χ^2 value of the optimized structure using a black solid line ($\Delta\chi^2 = 1$) and a black dashed line ($\Delta\chi^2 = 4$), respectively. In our minimization method, the maximum likelihood estimation method, the likelihood (L) is related to χ^2 through the equation: L $\propto$ exp($-\chi^2/2$). Considering this, the 1σ and 2σ regions, where σ represents the standard-deviation of the likelihood distribution, correspond to likelihood distributions of 68.3% and 95.4%, respectively. The regions corresponding to $\Delta\chi^2 = 1$ and 4 indicate 1σ and 2σ error ranges, respectively. Notably, the structural parameters optimized through the refinement process (indicated by vertical and horizontal red lines) demonstrate a remarkable alignment with the optimal structural parameters determined from the grid scan. This perfect agreement provides robust support for the validity of our structural refinement process.

Molecular dynamics (MD) simulation is a frequently employed technique to explore an extensive range of structural possibilities with high degrees of freedom. We conducted MD simulations and visualized the distribution of resulting structural snapshots within the conformational space. We performed *ab initio* molecular dynamics (AIMD) simulations at the DFT level, running for approximately 300 fs, commencing from the optimized structures of *iso*-DBP^+ and MBP^+. In both cases, we employed the CAM-B3LYP/def2-TZVPP DFT setup, which was also used for surface hopping dynamics, transition state and reaction pathways calculations. The distribution of the structural snapshots obtained from the AIMD simulations is shown in Supplementary Figs. 8 and 9 with black dots. Actually, some of the structural snapshots lie outside the parameter range we focused on in Supplementary Figs. 8 and 9, making them not visible in that figure. Importantly, Supplementary Figs. 8 and 9 substantiate that the structural snapshots encompassed by the AIMD simulations do not closely approach the global minimum identified through the grid scan.

Determination of the relative population of ions

The relative population of ions, which represents the molar ratio of photo-generated ions with respect to the DBP molecules at negative time delays as depicted in Fig. 3b, was determined

using the following procedure. First, we generated the experimental static I(s) curve by azimuthally averaging the static scattering pattern at negative time delay. The modified scattering curve, $s\mathrm{M}_{\mathrm{exp}}(s)$, was then calculated following the procedure described in section "Static radial-distribution-function (RDF) from the experimental data." Subsequently, a comparison was made between the modified scattering curve derived from the Debye equation for a DBP molecule in its ground state, denoted as $s\mathrm{M}_{\mathrm{DBP}}(s)$, and the static $s\mathrm{M}_{\mathrm{exp}}(s)$ curve. This comparison enabled the determination of the scaling factor between the experimental and theoretical curves. This scaling factor can be mathematically represented using the following equation:

$$s\mathrm{M}_{\mathrm{exp}}(s, t_{\mathrm{neg}}) = \alpha \cdot c_{\mathrm{DBP}} \cdot s\mathrm{M}_{\mathrm{DBP}}(s) \quad \text{(S15)}$$

where t_{neg} signifies to a negative time delay, denotes the scaling factor between the experimental and theoretical curves, and c_{DBP} represents the molar ratio of DBP molecules to the total gas molecules at a negative time delay, which is equal to 1. The term $\mathrm{sM}_{\mathrm{DBP}}(s)$ refers to the theoretical static scattering curve ($s\mathrm{M}(s)$ shown in Eq. S7), calculated for a DBP molecule by using Eqs. S5 and S6.

In the case of the difference modified scattering curve, $\Delta s\mathrm{M}(s,t)$, the experimental difference modified scattering curve can be expressed in relation to the relative population through the following equation:

$$\Delta s\mathrm{M}(s,t)_{\mathrm{exp}} = \beta(t) \cdot \left(s\mathrm{M}_{A^+}(s) - s\mathrm{M}_{DBP}(s)\right) + \gamma(t) \cdot \left(s\mathrm{M}_{B^+}(s) - s\mathrm{M}_{DBP}(s)\right)$$

$$= c_{A^+}(t) \cdot \alpha \cdot \left(s\mathrm{M}_{A^+}(s) - s\mathrm{M}_{DBP}(s)\right) + c_{B^+}(t) \cdot \alpha \cdot \left(s\mathrm{M}_{B^+}(s) - s\mathrm{M}_{DBP}(s)\right) \quad \text{(S16)}$$

where $\beta(t)$ and $\gamma(t)$ are the proportionality constants used to fit the experimental difference modified scattering curve, $\Delta s\mathrm{M}$, as a sum of two components: the difference modified scattering curves corresponding to species A^+ $\left(s\mathrm{M}_{A^+}(s) - s\mathrm{M}_{DBP}(s)\right)$ and species B^+ $\left(s\mathrm{M}_{B^+}(s) - s\mathrm{M}_{DBP}(s)\right)$. $c_{A^+}(t)$ and $c_{B^+}(t)$ denote the molar ratios of A^+ and B^+ ions to the total gas molecules at time t, respectively. Here, $\beta(t)$ and $\gamma(t)$ equal to the $c_{A^+}(t)$ and $c_{B^+}(t)$ multiplied by α, respectively. These molar ratios, $c_{A^+}(t)$ and $c_{B^+}(t)$, determined through the fitting of Eq. S17, accurately represent the relative populations of the ionic species depicted in Fig. 3b.

Computational details

The optimized molecular structures and relaxed potential energy surfaces (PESs) of DBP and DBP^+ were obtained by geometry optimization using density functional theory (DFT). The optimized stationary and transition state structures were confirmed by the vibrational frequency calculations. The resulting vibrational frequency values for the optimized structures are collected in Supplementary Table 7. Subsequently, the energies were corrected using complete active space self-consistent field (CASSCF) methods with CASPT2 corrections[16,59,60]. The CAM-B3LYP functional[61] combined with the def2-TZVPP basis set[62] was used for all DFT calculations, while the CASSCF calculations employed an active space composed of 8 active electrons and 8 active orbitals (Supplementary Fig. 12) with the aug-cc-pvtz basis set[63,64]. The CASPT2 energies were then computed applying an imaginary level shift of 0.2 a.u. to minimize the presence of weakly-interacting intruder states.

The ionization-induced dynamics of DBP^+ were simulated via on-the-fly non-adiabatic simulations using spin-unrestricted time-dependent density functional theory (UTDDFT), with the same functional and basis set employed in the DFT calculations. Our simulations incorporated non-adiabatic events up to the D_2 state and utilized the fewest switches surface hopping (FSSH) approach with decoherence corrections[15,65]. Additionally, non-adiabatic couplings between doublet states were computed using the finite difference method proposed by Hammes–Schiffer and Tully, and the Casida ansatz was employed to represent the TDDFT wavefunctions[15,66]. Due to the intrinsic limitations of DFT in describing the strong multireference character of the ground state near the D_1/D_0 intersection, no couplings to the doublet ground state were computed. In this context, it is commonly understood that the attainment of D_0 can be determined when the SCF calculation ceases to converge, indicating a very small energy gap (approximately 0.05 eV) between D_1 and D_0.

The initial conditions for the two most stable DBP conformers were generated by sampling the coordinates and momenta so as to reproduce the ground vibrational quantum harmonic distribution of the electronically neutral ground state by means of a Wigner distribution[67,68]. Our decision to sample the initial conditions using the frequencies associated with the electronic state of the neutral DBP was based on the assumption that the molecule

would not have sufficient time to reorient itself during the [2+1] REMPI process. The dynamics were conducted for a maximum of 1 ps and the integration of the nuclear motion was performed by means of the Velocity-Verlet algorithm using a time step of 0.5 fs[69,70].

In order to gain a comprehensive understanding of the detailed reaction dynamics of the observed species from experimental data, we performed computations to determine the transition states and intermediates associated with the chemical reaction. To ensure the presence of the calculated structures along the desired pathway, we validated that the respective transition state connected the observed reactant and product by applying the Intrinsic reaction coordinate (IRC) calculations[71]. The results are shown in Extended Data Fig. 8. We conducted additional vibrational frequency calculations to confirm whether the transition state structures assigned based on IRC calculations correspond to actual transition states. As a result, we verified that all obtained transition state structures possess only one imaginary frequency (Supplementary Table 8). For the geometry optimization and IRC calculations of transition states, the identical basis set and functional mentioned above were employed during the DFT calculation.

We conducted calculations to both confirm the existence of and to identify a conical intersection connecting the D_1 state and *iso*-DBP^+ in the D_0 state. For that purpose, we first postulated that this conical intersection might be located near the transition state determined from the IRC calculations. Specifically, we proposed that the conical intersection could be in close proximity to TS1, which links the DBP^+ in D_0 state with *iso*-DBP^+. Using the geometry of TS1 as a starting point, we aimed to find the DBP^+ geometry where the energy difference between the D_0 and D_1 states is minimized. We found a CI that closely resembles the TS1 geometry (Supplementary Table 5). The relative energy of the determined conical intersection (CI1) is depicted in Supplementary Fig. 18. CI1 has approximately 4.3 kcal/mol higher energy than the D_2 state. The calculations for finding CI1 were performed at the XMS-CASPT2//CASSCF level.

The Gaussian16 software was utilized for all DFT calculations[72], whereas the Bagel code was employed for CASSCF, CASPT2 and XMS-CASPT2 calculations[73]. We employed the NewtonX package, interfaced with Gaussian16, for conducting surface hopping simulations[15].

Details of potential energy scans

A series of relaxed PES scans were calculated for the ground state (GS) and the energies at these structures were calculated for the GS and the first four ionic doublet states (D_0, D_1, D_2, D_3) using the CASPT2 and the XMS-CASPT2 method, respectively. The results of this scan is shown in Fig. 5. The same approach was also used to investigate the PES scans for propane and 1,3-dichloropropane (Extended Data Fig. 9). In order to fully describe the potential energy surfaces, a 2D scan was conducted involving the dihedral angles Br_1-C_1-C_2-C_3 and Br_2-C_3-C_2-C_1 (atom numbers are shown in Fig. 4c). After locating the minima, the corresponding geometries of DBP were extracted and utilized to perform a full relaxation of the DBP geometry for all electronic states. Supplementary Table 3 displays the geometric parameters of DBP for all the electronic states that were taken into consideration. For the ground state, it is worth mentioning that the structure of DBP has been optimized for the three most stable conformers, namely AA, AG, and GG. From the geometrical parameters listed in Supplementary Table 3, it can be observed that the global minima of D_1 and D_2 have a geometry similar to the GG and AG conformers of S_0, respectively; while the global minima for D_0 and D_3 are at conformations completely different from the three conformers (AA, AG, and GG) of the neutral ground state. The 2D scan was performed with a dihedral angle step of 5 degrees. Some points along the scan did not achieve convergence at the XMS-CASPT2 level. The built-in MATLAB function 'fillmissing' with the piecewise cubic spline interpolation was used to fill the missing data points.

This finding is significant as the extended induction time observed after DBP ionization implies that the ionic state attained by DBP must have a minimum with a nuclear configuration comparable to that of the GS. The PESs obtained at the XMS-CASPT2 level in this study serve as a reference for evaluating various DFT functionals and identifying the optimal functional that can reproduce similar accuracy. The combination of CAM-B3LYP functional with def2-TZVPP basis set provides an accurate description of the PESs for both the ground state and doublet states, as shown in Supplementary Fig. 10. Based on this benchmark, we use this functional for the NAMD simulations.

Details of Dyson orbitals

The Dyson orbitals are utilized to effectively describe the ionization transition from a wavefunction having N electrons to one with N-1 electrons. These orbitals are calculated by determining the overlap between the initial (N electrons) and final wavefunctions (N-1 electrons)[38,39]. Since the wavefunctions differ by only one electron, the overlap is a one-particle wavefunction rather than a scalar. The intensity of the ionization channel can be determined by the norm of Dyson orbitals. In the case of Koopmans-allowed transitions, the norm is approximately equal to 1, while it is close to 0 for forbidden transitions[38,39]. Apart from their quantitative applications, Dyson orbitals provide qualitative insight by illustrating the distinctions between molecular and ionized states. In ionization processes, these orbitals can be considered as the wave function of the ejected electron[38,39].

In this context, Dyson orbitals were employed to determine which DBP^+ doublet state is more readily populated after the ionization of the DBP molecule. We calculated the Dyson orbitals between the first Rydberg state and the first eight doublet states since in the [2+1] REMPI process ionization is achieved only after reaching the Rydberg state.

As an example, the Dyson orbitals between the D_2 state of DBP^+ and the first Rydberg state of DBP for the three DBP conformers (GG, AG, AA) are reported in Supplementary Fig. 11 and the norm of each Dyson orbital for all eight doublet states is reported in Extended Data Table 1. The calculation of these norms displays that the D_0, D_1, and D_2 states are the major products of the [2+1] REMPI, although, as stated, only D_2 is almost equally accessible for all conformers of DBP, whereas the probability to access the first two doublet states highly depends on the considered conformers. If a certain conformer is selectively formed, it would give a discernable signal in the UED data. The induction period means that the relative ratios of the conformers in the neutral DBP in the S_0 state should be maintained in the generated DBP^+. This consideration adds support to D_2 as the major state initially populated upon [2+1] REMPI, given its analogous potential energy landscape to that of the S_0 state (Fig. 5a). In summary, based on the comparison between PESs and the norms of Dyson orbitals and the consideration of the relative norms of all conformers, we conclude that the D_2 state, which has a PES similar to that of S_0 and a high transition rate from the Rydberg state, is the state that is populated with the highest probability and most likely responsible for the initial induction

period. In addition to assisting in the assigning of the initial state populated upon REMPI, the Dyson orbitals illustrated in Supplementary Fig. 11 indicate that the ionization process of DBP occurs by removing an electron from a state mainly associated with the bromine atoms.

Details of surface hopping simulation

We created one set of 100 initial conditions (ICs), consisting in structure and velocity, for the most stable conformers of DBP, namely the GG. These ICs were generated by sampling the ground state minimum via a harmonic-oscillator Wigner distribution. Although we simulated the excited state dynamics starting from excited doublet states, the initial conditions have been obtained by using the normal modes at S_0 since we assumed that the structural changes begin only after the ionization process has occurred.

By combining the analysis of the Dyson orbitals with a comparison of the PESs, we can determine the initial state from which to commence non-adiabatic dynamics. As the D_2 state has a comparable population among all conformers, we have selected it as the starting point for propagating the excited state dynamics. Additionally, since the other doublet states that can be occupied, according to the norms of Dyson orbitals, are at lower energies than the D_2 state, this approach enables us to track the dynamics in those states as well.

To investigate the reasons behind the induction time, we performed surface hopping simulations and examined the reaction pathway and transition rate of DBP^+[40,41]. For the two most stable conformers of DBP, the simulations were carried out up to 1 ps, considering D_2 as the initial active state. The analysis of these trajectories shows that DBP^+ does not exhibit noticeable structural changes for several hundred fs after excitation, as evidenced by the computed averaged difference scattering curve (Extended Data Fig. 7). Since we can consider that non-radiative relaxation is achieved within the considered simulation time (1 ps) for all trajectories, we can conclude that the DBP^+ in D_2 can reach D_1 and D_0 through a decay pathway that does not involve large structural changes, and this consistently explains the long induction time. Namely, the doublet states involved in the photodynamics, i.e. D_2, D_1, and D_0, show global or relative minima at very similar DBP nuclear coordinates, and therefore, the majority of the trajectories describe DBP^+ reaching D_0 with none or negligible conformational changes.

Additional information regarding the NAMD simulations is provided below. Although this simulation alone supports the possibility of reaching D_0, our UED data support that the transformation into *iso*-DBP^+ should occur before DBP^+ reaches D_0 due to the following consideration. The UED data show that the ion species formed upon REMPI and present during the induction period are not only structurally indistinguishable from the neutral species but also relatively cold. If the ion species in D_2 reaches D_0, the energy difference between the two states should be converted into thermal energy. This energy difference is higher than the barriers between conformers, and therefore hot species with large DW factors should be formed. On the contrary, the UED data does not exhibit such large DW factors during the induction period. Since the energy of D_1 is very close to D_2, conversion to D_1 would not induce significant thermal energy. Therefore, the induction period can be largely accounted for by the conversion time from D_2 to D_1. Then, the transformation to *iso*-DBP^+ is likely to occur from D_1 via a conical intersection.

Emission lifetime

We have assessed the emission lifetimes of DBP^+ by applying Fermi's golden rule, utilizing the electronic structure properties calculated at both the CASSCF and XMS-CASPT2 levels and using the computational setup discussed above. In this approach, each excited state (ES) with a non-zero transition dipole moment possesses a radiative recombination rate, known as the Einstein coefficient (A_{10}), where 1 and 0 correspond to D_1 and D_0 in our context. Therefore, the emission lifetime is determined as the reciprocal of this coefficient using the following formula

$$\tau_{(1\to 0)} = \frac{1}{A_{10}} = \frac{2\pi\varepsilon_0 m_e c^3}{n\omega^2 e^2 f_{(1\to 0)}} \quad \text{(S17)}$$

where ε_0, m_e, c^3, and e are the familiar fundamental constants, n is the refractive index of the medium (set to 1), ω is the excitation energy, and $f_{(1\to 0)}$ the oscillator strength.

Supplementary Discussion

Comparison of the observed induction period with previous studies

The feature of no significant structural change in the initial few ps, despite the highest internal energy of the molecule, followed by a sudden large change, is an unusual case in reaction kinetics. While there have been reports on the initial induction period of photoreaction pathways, the reported induction periods were less than 1 ps[74-76]. This extraordinarily long induction period differs from the results of time-resolved studies using mass spectrometry for DBP^+. In that study, a weak oscillatory signal with a period of 700 fs was observed in the ion yield[24]. By comparing the observed period of 700 fs with the theoretically calculated periods of normal modes for Rydberg states, they attributed this feature to a torsional vibration of the bromomethylene groups. In contrast, no significant difference scattering signal was observed in our UED experimental data during the induction time. This difference is likely due to the fact that the UED technique, which uses an electron scattering probe, is sensitive only to the "structure of the molecule," unlike mass spectrometry. The vibrational motion of DBP observed by time-resolved ion photofragmentation spectroscopy and time-resolved photoelectron spectroscopy does not seem to significantly contribute to the signal in UED, which is sensitive only to the structural aspect of the molecule[24]. Two possible interpretations can be considered for this discrepancy: 1) The amplitude of the observed bromotorsional motion might be extremely small for detection by UED with the available SNR, 2) The motion might involve only light atoms that do not significantly contribute to UED signals, apart from Br.

Comparison with a previous study on ions using UED

To study short-lived ion intermediates, a range of spectroscopic techniques, including time-resolved photofragmentation (TRPF)[24,77] and X-ray transient absorption (XTA) spectroscopies[23,78] have been employed. While TRPF is highly sensitive to ionic species and their kinetics, it does not provide direct information on the transient structure of these intermediates.

For this reason, in a previous study, UED was employed to observe various ion species generated from toluene molecules[20]. To generate a significant quantity of ions, a high-intensity 800 nm pulse with 1.2 mJ was focused to dimensions of 100 × 170 μm^2 to induce non-resonant strong-field ionization of toluene molecules, thereby facilitating the production of a substantial number of ions. This approach effectively generated a notable ion population, yielding discernible ion signals within the diffraction pattern. However, strong-field ionization using non-resonant excitation made the selective formation of desired ions problematic. The fluence utilized in this research, exceeding 7-fold at 8,987 mJ/cm^2 (= 90 mJ/mm^2) compared to our experimental conditions, resulted in the generation of a mixture of diverse ion species, including fragmented species and doubly-ionized molecules as well as singly-ionized species. Hence, while successful in generating a large ion quantity, experiments struggled when aiming to establish dominance of a specific ion species under observation. Given the presence of diverse, mixed ions under the experimental conditions, it was challenging to acquire information about structural changes or kinetics of the ions generated beyond the formation of ions. Consequently, aspects concerning dynamics and kinetics lay outside the scope of their investigation. This specific aspect becomes evident due to the limited number of collected experimental data points, encompassing only four time points (-5 ps, 5 ps, 10 ps, and 15 ps), wherein no substantial change in the diffraction pattern was discernible across different positive time delays.

For the direct exploration of ion dynamics via UED that captures structural changes, generating a substantial quantity of ions is critical. Furthermore, it is crucial to maintain conditions that gently ionize the neutral molecules so that solely the desired ions of interest can be generated. We sought to address both these prerequisites concurrently by employing the REMPI process. REMPI is noted for its high molecular ion formation rates in diverse research domains such as mass spectrometry, rendering it a gentle ionization method with relatively elevated ionization yields. This strategy enabled us to realize the generation of a substantial quantity of singly ionized molecular ions of interest.

Deflection of the electron beam and scattering profile due to the generation of ions

In the presence of ions, the path of probe electrons can be altered, leading to deflection towards a specific direction. Therefore, we expected to observe an asymmetric intensity distribution over the azimuthal angle (φ) in scattering patterns at positive time delays, where φ represents the angle between a directional vector on the detector plane from the beam center to the detector pixel and the direction of the horizontal axis. Our observations confirmed this hypothesis: at negative time delays, the electron beam profiles were isotropic over the φ angle, whereas at positive time delays, they exhibited an asymmetric feature with respect to the beam center (Extended Data Fig. 1). An important aspect of this asymmetry is that not only does it affect the difference scattering pattern, but it also alters the position of the direct beam that did not scatter, deviating from the original beam center position at negative time delays (Extended Data Fig. 2). This finding indicates that the observed asymmetric feature is caused by deflection due to ionic species generated through [2+1] REMPI. Coulomb interaction between the electron beam and the ions results in deflection, which manifests as an asymmetric feature.

Quantitative analysis of the static RDF of the ground state DBP

To quantitatively analyze the changes in ΔRDF(r,t) that represent structural changes, it was necessary to first establish the structural parameters of the ground state by analyzing the static RDF(r) before the reaction. This served as the basis to extract information about the structural parameters of the transient species from the ΔRDF(r). The analysis of the ground state was conducted by first analyzing the static curve, and the resulting static RDF(r) (ground-state DBP's RDF) was shown at the top of Fig. 3c. To analyze the ground-state DBP's structure, it is important to take into account previous findings that identified three conformers in the ground state, namely GG, AG, and AA, which correspond to gauche-gauche, anti-gauche, and anti-anti geometries, respectively. To describe the experimental data of the ground state, all three conformers were considered, and their structures were optimized through DFT calculations to calculate their theoretical sM(s)'s. The linear combination of these theoretical sM(s)'s was compared with the experimental static sM(s), and the ratios of the conformers that minimize the difference (χ^2) between the two were determined. According to the analysis, the ground-state DBP exists in the ratio of 66 (± 2.3) %: 20 (± 1.5) %: 14 (± 2.7) % for GG, AG, and AA,

respectively, which is similar to the results of previous studies that reported ratios of GG: AG: AA = 67%: 30%: 3%[27].

Qualitative analysis of ΔRDF(*r*) of species A^+ and B^+

Before conducting quantitative structural analysis, we first qualitatively analyzed the structural features of the two species by examining their ΔRDF(*r*). For ground-state DBP, there are three representative atomic pairs, bonded C–Br, Br–Br, and non-bonded C–Br, that contribute to the RDF(*r*) (Fig. 4c, highlighted by colored arrows). These pairs are at approximate distances of 2.0, 3.0, and 4.5 Å, respectively. When a structural change occurs due to photoionization, the bond lengths in the DBP also change. Therefore, the bond lengths identified as positive peaks in the RDF(*r*) of ground-state DBP appear as negative peaks in the ΔRDF(*r*) (Fig. 3c). It is confirmed that the positions of the broad negative peaks observed in species A^+ and B^+ are consistent with the bond lengths of ground-state DBP mentioned earlier (Fig. 4a and 4b). In particular, the broad negative peaks around 3 - 4 Å in the ΔRDF(*r*) in Figs. 4a and 4b indicate that some atomic pair distances, such as non-bonded C–Br and Br–Br pairs, disappeared upon photoinduced ionization in both species A^+ and B^+. For species A^+, the two positive peaks around 1.8 and 6 Å indicate that some short and long atomic distance pairs have newly appeared. The presence of a positive peak at approximately 1.8 Å implies the formation of an additional short-distance pair, possibly indicative of a short-distance C–Br pair. The presence of a peak at a longer distance of approximately 6 Å, a distinctive feature of the loosely bound atom, should be noted. The ΔRDF(*r*) of species A^+ suggests that the most probable form of species A^+ is the Br-bounded MBP^+. For species B^+, such positive peaks are hardly found, and only broad negative peaks are observable. Negative peaks without any corresponding positive peaks are typical features of molecular fragmentation. For example, the negative peak around 4 - 5 Å with no additional positive peak means that the Br–Br pair disappeared from the ion without forming any pairs. Thus, one of the Br atoms should have completely disappeared from the ion, forming MBP^+. The recovery of the non-bonded C–Br pair (~3 Å) and concomitant reduction of the shorter C–Br pair (~2 Å) in species B^+ is indicative of the dissociation of the C–Br bond (~2 Å) in species A^+, forming a longer distance pair (~3 Å) in species B^+.

Comparison of the observed structure of bromonium MBP^+ with those in crystals

The structure of B^+, as indicated by its name, "bromonium MBP^{+}", resembles that of a bromonium ion. A bromonium ion is an intermediate formed during the addition reaction of bromine to an alkene species with a C–C double bond. Numerous studies have examined halonium ions, establishing that these ions are a crucial intermediate in diverse organic reactions, including alkene addition reactions[9,10] and are most stable among observed cations due to the π-donation of the halogen atom's lone pair to the carbocation, as opposed to the carbocation where the positive charge is confined to carbon. Furthermore, these halonium ions were observed as the final long-lived intermediate in the relaxation cascade of highly excited cations, indicating that they have high stability compared to other cationic species. Nevertheless, the high reactivity of halonium ions in addition reactions has made direct observation of their structures elusive. Notably, we were able to elucidate the structural and energetic aspects of the intermediate in the absence of solvent effects. By capturing the same ion under solution conditions and comparing the findings, we can investigate the influence of solvents on the relative stability of the observed cationic species. The process by which this intermediate is formed and its structure have not been directly determined. Previous studies have created crystals containing the stable salt form of the bromonium ion and used crystallography techniques to study the structure[19]. However, the structure of the salt form in the crystal can be influenced by the counterion and may differ from the structure of the bromonium ion formed during the reaction. The difference in structure between the crystal and the reaction intermediate has been observed in previous studies, such as with the anion I_3^-[7,79]. Furthermore, the structure of the bromonium ion in the stable form can also vary depending on the type of counter ion[19]. Therefore, in this study, we compared the structure reported in crystallography with the structure obtained from our UED experiment and found a significant difference. Specifically, in the presence of the counterion in the crystal, the C–C bond corresponding to $r\mathrm{C_{B1}C_{B2}}$ is 1.50 Å and $r\mathrm{C_{B1}Br_{B1}}$ is 2.1 - 2.2 Å, but our UED data shows values of 1.74 ± 0.05 Å and 1.96 ± 0.01 Å, respectively, with an elongation of approximately 0.2 Å from the crystal structure. This comparison confirms that the structure of the bromonium cation as an intermediate in the chemical reaction differs significantly from the structure of the bromonium salt in the crystal in its stable form. It should be noted that the bromonium cation observed in our study lies in its electronically ground state. This means that it shares the same

identity as the bromonium ion formed during conventional organic reactions, except that there is no solvent in our experimental condition, offering an opportunity to identify the effect of solvent molecules on the molecular structure of the bromonium ion.

Overall structural pathways determined from the kinetic and structural analysis

By quantitatively analyzing the high-quality UED data, we discovered: (1) the detailed kinetic information of the structural pathways of DBP upon ionization, and (2) the atomistic details of the structure of each intermediate involved in the structural pathways. The overall excited-state pathways determined from the kinetic and structural analysis can be summarized as follows. Our UED data unveils both the identity and the detailed molecular structure of the two cationic intermediates, *iso*-DBP^+ and bromonium MBP^+, involved in the photoinduced ionization pathways of DBP. First, following the instantaneous [2+1] REMPI process, there exists an initial induction period of 3.6 ps where the ionized DBP^+ maintains its structure that is almost the same as those of neutral DBP in the ground state. Such a long induction period without a structural change is exceptional, considering that DBP^+ should have a high internal energy immediately after ionization. After that, DBP^+ shows a structural rearrangement with a time constant of 15 ps, yielding an intermediate with a long Br–Br distance, the *iso*-DBP^+. The broad positive peak observed in the experimental ΔRDF(r) (Fig. 4a, at r around 6 Å, indicated by a dotted purple line) indicates the formation of a long Br–Br pair distance and thereby supports our assignment. Finally, complete dissociation of the Br atom occurs with a time constant of 77 ps, resulting in the MBP^+ with a three-membered ring, bromonium MBP^+, as the second intermediate. As Figs. 4d and 4e show, the structures of the two cationic intermediates, *iso*-DBP^+ and bromonium MBP^+, were determined with a structural resolution of better than 0.01 Å. In Supplementary Fig. 10, we present the determined structural parameters and structure for the optimized structure of each intermediate. Our theoretical investigation through *ab initio* calculations and surface-hopping simulations supports our observations. The result of *ab initio* calculations justifies the exceptionally long induction period upon photoionization as the shape of PES of one of the cationic excited states, D_2, is similar to that of S_0, which provides a clue for understanding a long period without a significant structural change.

It should be noted that our experimental approach does not involve any form of molecular blasting, which refers to a violent and uncontrolled fragmentation or atomization resulting from excessive energy, and none of the intermediates, including the observed bromonium cation, possess excessive excess energy. The induction period signifies that the generated DBP^+ does not carry excess energy capable of inducing instant, significant structural changes or broadening the structural distribution in the form of increased mean-squared displacement of the Debye-Waller factor. Notably, the absence of significant oscillatory features in the high-*s* region of the experimental data during the induction period, as illustrated in Extended Data Figs. 4 and 5, corroborates this interpretation. Furthermore, as depicted in Fig. 5 and Supplementary Fig. 18, the reactions we observed occur distinctly through a well-defined pathway. This strongly signifies that the reactions we observed are not linked to chaotic processes occurring at excessively high energies—instead, they are well-organized reactions governed by the shape of the potential energy surface. Our structural analysis underscores that the identified reaction intermediates (*iso*-DBP^+ and bromonium MBP^+) exhibit minimal structural fluctuations attributable to thermal energy. The reason molecules could undergo soft ionization without being blasted is attributed to our utilization of the REMPI technique. REMPI not only enabled the formation of a significant quantity of desired target ions but also facilitated soft ionization, ensuring that the generated ions proceed through a well-defined pathway.

To the best of our knowledge, this work is the first demonstration of the observation of the real-time, ultrafast structural dynamics of ionic species in the gas phase, which, to date, have not been realized due to experimental difficulties. In this work, we overcame the experimental obstacles by combining ultrafast electron diffraction, which enables direct detection of structural changes of the dilute molecules in the gas phase, and the generation of molecular ions via multiphoton ionization. This methodology should pave the way to identifying the structural dynamics of gas-phase ions, which are critical for understanding atmospheric chemistry and astrochemistry.

Existence of the induction period

The presence of an induction period indicates that the ion species in this period should possess a molecular structure that is indistinguishable from that of the ground state. In addition, the

ratios of conformers should be maintained. Otherwise, there should be clear changes in the overall UED signal, even if the molecular structure of each individual conformer does not change from the neutral molecule to the ion species. Moreover, the induction period suggests that the ion species in the induction period should exhibit Debye-Waller (DW) factors (Eq. S13) similar to those of ground-state species. A significant change in DW factor, such as significantly larger DW factors in the ion species compared to the ground state, would also lead to observable changes in the overall UED signal. The observation that the dark states possess typical DW factors indicates that these states are relatively cold, lacking excessive thermal energy. This further implies that the excess energy, resulting from the difference between the energy of three photons and the energy needed to generate DBP^+ ions, is primarily converted into the kinetic energy of the ejected photoelectron, rather than contributing to the thermal energy of the cation.

Structure of the Rydberg state

The induction period presents at least two possibilities. One possibility is that the Rydberg state has a distinct structure from that of S_0, but is not significantly populated. Another possibility is that the Rydberg state generated by two-photon absorption remains structurally dark, similar to the cations formed by [2+1] REMPI. It is possible that the conversion yield of the Rydberg state to DBP^+ cannot reach 100%. If so, a portion of the DBP within the Rydberg state will persist in the state, while the rest will ionize into DBP^+. If the Rydberg state were not structurally dark, the UED data would have exhibited a noticeable difference scattering signal around time zero, indicating structural changes within the Rydberg state. The absence of such signals suggests that the Rydberg state is structurally dark. In other words, the DBP in the Rydberg state remains in a cold state and does not undergo detectable structural changes. The combination of both scenarios is also a plausible explanation.

Results of surface hopping simulations

The objective of the non-adiabatic surface hopping investigation is to unravel the reaction pathways of DBP^+ and verify the experimentally observed long induction period. Firstly, as evidenced in Supplementary Fig. 14, we observe that for all the trajectories analyzed,

photorelaxation occurs within 1 ps, with 50% of the trajectories reaching D_0 within the first 300 fs. Despite initiating all simulations from D_2, the narrow energy difference among doublet levels, including D_1 and D_2, leads to a consistent population of electronically excited doublet states throughout the simulations, as the absence of constraints allows the re-population of D_2 once D_1 is reached. Supplementary Figs. 15–17 highlight that most of the trajectories analyzed have little or no changes in the geometric structure of DBP^+. Only a very small number of trajectories, specifically 2, show the detachment of a bromine atom from the carbon skeleton of DBP^+. Moreover, in just one case, we observe the subsequent approach of the remaining bromine atom to the furthest carbon, indicating the potential formation of iso-DBP^+ (Supplementary Fig. 17a). Surface hopping dynamics were also employed to calculate the simulated $s\Delta I_0(s,t)$ depicted in Extended Data Fig. 7. The calculated $s\Delta I_0(s,t)$ shows minimal changes in structure throughout the non-adiabatic dynamics and does not indicate the formation of the experimentally observed products. Based on these analyses, it can be concluded that the likelihood of *iso*-DBP^+ formation during the photorelaxation process is extremely low. This is due to the fact that the photorelaxation process takes place within 1 ps, which is significantly shorter than the experimental kinetic constant. Moreover, no statistically significant structural alterations are observed, indicating the absence of the desired photoreaction.

For most trajectories, DBP^+ starting from D_2 reaches D_0 without involving any significant structural changes, and therefore, the majority of the trajectories describe DBP^+ reaching D_0 with none or negligible conformational changes, which explains the long induction time. However, the results of surface hopping simulations suggest that the formation of *iso*-DBP^+, after the long induction time, originates exclusively from D_0, not from D_1 or D_2. This contradicts the experimental findings. According to the interpretation of experimental data, the most plausible explanation is that the transition to *iso*-DBP^+ occurs from the D_1 state via a conical intersection. However, our surface hopping simulations did not show such a transition from D_1 to *iso*-DBP^+. This discrepancy is likely attributable to the inherent limitations of TDDFT in describing conical intersection, which involves wavefunctions having a complex multiconfigurational nature. To substantiate our hypothesis, we conducted additional calculations aimed at locating a conical intersection between D_1 and D_0 states. We discovered a conical intersection (referred to as CI1) having a structure similar to the one of the first transition state (referred to as TS1). Details regarding the computational methods used to locate

CI1 and the correspoding results are available in the "Computational details" section of SI. The determination of TS1 connecting D_0 and *iso*-DBP^+ was accomplished through IRC calculations.

Reaction pathways from D_2 state to *iso*-DBP^+

As mentioned in the main text, the D_2 state stands out as the primary initial energy state followed by [2+1] REMPI ionization. Prior to the structural transition leading to *iso*-DBP^+, the D_2 state, due to its high energy, generally undergoes a transition to lower doublet states. Among the transitions, the conversion from D_2 to D_1 is most likely responsible for the initial induction period (Fig. 5b). The conversion to D_0 is inconsistent with the observed induction period. This conversion could occur through two possible pathways: radiative and non-radiative. Regarding the radiative conversion, we estimated the lifetimes for the transitions from D_2 to D_0 and D_1 to D_0, respectively, and found that the values are quite long, significantly exceeding 1 microsecond (Supplementary Table 6). Therefore, radiative decay to D_0 is highly unlikely. In the case of non-radiative decay to D_0, if such a conversion were to take place, given the substantial energy difference between D_2 and D_0 (~8.3 kcal/mol), the resulting D_0 would acquire significant thermal energy. It is worth noting that the height of the barrier between local minima and global minima in the D_0 state is less than 5 kcal/mol, whereas in the D_1 or D_2 state, it is significantly higher, approximately 10 kcal/mol. Considering this, it becomes evident that the thermal energy of ~8.3 kcal/mol obtained by DBP^+ upon non-radiative decay to D_0 would enable DBP^+ to overcome energy barriers and explore other local and global minima, ultimately resulting in the formation of alternative structures. This scenario contradicts the observed induction period. In this regard, it should be noted that the energy difference between D_2 and D_1 is small (~4.6 kcal/mol). Then, the most probable route for the formation of *iso*-DBP^+ is via a conical intersection connecting D_1 to *iso*-DBP^+.

Reaction pathways to form *iso*-DBP^+ and MBP^+

Based on the results from IRC calculations, the Br_1–C_3 distance is identified as the reaction coordinate that governs the production of the 4-membered ring of *iso*-DBP^+. An SN_2 type of intramolecular nucleophilic substitution takes place in DBP^+, resulting in the formation of a

new C–Br bond when Br_1 attacks C_3, creating a 4-membered ring intermediate. This, in turn, initiates the dissociation of the other C–Br bond, leading to the isomerization of DBP^+. In the resulting *iso*-DBP^+, the two Br atoms are separated by a long distance (5.6 Å), which is remarkably similar to ~5.9 Å, the experimentally observed distance of the "loosely bound state"[17,18]. The dissociated Br radical is weakly bound to the other counterpart, the 4-membered ring MBP^+, with a binding energy of ~2 kcal/mol. Eventually, the loosely-bound Br in *iso*-DBP^+ escapes, yielding the bromonium MBP^+ with a time constant of 77 ps. The quantum calculations show that MBP^+ with a three-membered ring (Br_{B2}–C_{B1}–C_{B2}) is most stable among the three cationic MBP structures considered in the structure refinement. Its energy is significantly lower than that with a 4-membered ring (Br_1–C_1–C_2–C_3) resembling the structure of the MBP^+ moiety in *iso*-DBP^+ (Supplementary Fig. 18). This result is consistent with the structure of the MBP^+ intermediate with a 3-membered ring determined via fitting the SADS of B^+ (Fig. 4e). The formation of bromonium MBP^+ necessitates the concomitant proton (or hydrogen) transfer during the breakage and formation of a three-membered ring structure. In order to enhance our comprehension of the reaction dynamics, we performed calculations of transition states and an intermediate utilizing IRC[71], which enabled us to trace the trajectory of MBP^+ formation. Although it is unusual for proton (or hydrogen) transfer to occur over such a long time period of 77 ps, the calculated reaction pathways provide an explanation. The 4-membered ring of *iso*-DBP^+, which is an exotic but quite stable structure, initially breaks down via a transition state (TS2 in Supplementary Fig. 18) with a relatively high activation barrier (~15 kcal/mol), inducing the breakage of one of the C–Br bonds and electron deficiency in the terminal C atom becoming the γ-carbon. Consequently, one of the hydrogens in the central carbon is transferred to the γ-carbon to mitigate the electron deficiency. The resulting intermediate is unstable since it has a destabilized central carbon atom, and subsequently, the final product, bromonium MBP^+, is hence rapidly formed via another transition state (TS3) with a much lower activation barrier (~3 kcal/mol). Since TS2 has a much higher activation barrier than TS3, the rate for the conversion from *iso*-DBP^+ to bromonium MBP^+ is determined by the reaction involving TS2. The mechanism described above explains well how this reaction occurs; the long induction time (77 ps) and the high energy barrier seem to coincide in a slow and energetically expensive process which therefore struggles to start due to a non-negligible stability of the *iso*-DBP^+. However, it is possible that the reaction intermediate preceding MBP^+ is reached via intramolecular proton (or hydrogen) tunneling, which may be triggered by the

fluctuation of one of the two C–Br bonds and/or by the change in the position of the dissociated bromine atom. To confirm the predominant mechanism between these two, conducting extensive and highly accurate excited-state MD simulations are required. Nonetheless, this task exceeds the scope of the current study.

Loosely bound nature of *iso*-DBP$^+$ supported by calculated vibrational frequencies

Our geometric optimization of *iso*-DBP$^+$ revealed an unusually extended Br–Br distance in its structure. Subsequent frequency calculations provided crucial insights, confirming that this elongated Br–Br distance is maintained by a significantly shallow well on the potential energy surface. In particular, we observed three low-frequency vibrational modes associated with the displacement of the distant Br atom in the *iso*-DBP$^+$ species (Supplementary Table 7). These low-frequency modes offer compelling evidence supporting the existence of a shallow potential well characterized by a minimal gradient, where the distant Br atom resides. This evidence substantiates our characterization of the distant Br atom as "loosely bound" to its neighboring atom(s).

Potential generality of ionized state dynamics

To investigate the generality of ionized state dynamics and address the exceptionally long induction period, we extended our analysis to other ionized systems, specifically 1,3-dichloropropane and propane. As depicted in Extended Data Fig. 9, we present the PESs corresponding to the electronic ground state (S_0) and the first four doublet states (D_0, D_1, D_2, and D_3) for 1,3-dichloropropane and propane, as well as DBP. Notably, the PESs of 1,3-dichloropropane and propane exhibit similar trends to those of DBP, with the positions of the global or local minima on the neutral ground-state PES maintained even in the PESs of the ionic states. The fact that even propane displays consistent PES patterns suggests that the ionized species resulting from the removal of a single electron from a neutral species may have a highly similar molecular structure to the original neutral species, pointing toward a possible general phenomenon. Thus, it is probable that other hydrocarbons can show a prolonged induction period although further experiments are necessary to provide empirical evidence.

References

[42] Shen, X. *et al.* Femtosecond gas-phase mega-electron-volt ultrafast electron diffraction. *Struct. Dyn.* **6**, 054305 (2019).

[43] Bucksbaum, P. H., Ware, M. R., Natan, A., Cryan, J. P. & Glownia, J. M. Characterizing Multiphoton Excitation Using Time-Resolved X-ray Scattering. *Phys. Rev. X.* **10**, 011065 (2020).

[44] Wilkin, K. J. *et al.* Diffractive imaging of dissociation and ground-state dynamics in a complex molecule. *Phys. Rev. A* **100**, 023402 (2019).

[45] Natan, A. *et al.* Resolving multiphoton processes with high-order anisotropy ultrafast X-ray scattering. *Faraday Discuss.* **228**, 123-138 (2021).

[46] Ruddock, J. M. *et al.* A deep UV trigger for ground-state ring-opening dynamics of 1,3-cyclohexadiene. *Sci. Adv.* **5**, eaax6625

[47] Yang, J. *et al.* Diffractive imaging of a rotational wavepacket in nitrogen molecules with femtosecond megaelectronvolt electron pulses. *Nat. Commun.* **7**, 11232 (2016).

[48] Boesl, U., Weinkauf, R. & Schlag, E. W. Reflectron time-of-flight mass spectrometry and laser excitation for the analysis of neutrals, ionized molecules and secondary fragments. *Int. J. Mass Spectrom. Ion Processes* **112**, 121-166 (1992).

[49] Johnson, P. M. Molecular multiphoton ionization spectroscopy. *Appl. Opt.* **19**, 3920-3925 (1980).

[50] *Laser Ionization Mass Analysis*. (A Wiley Interscience Publication, 1993).

[51] Kim, J. & Kim, S. K. Multiphoton-excited dynamics of the trans or cis structural isomer of 1,2-dibromoethylene. *J. Chem. Phys.* **155**, 164304 (2021).

[52] Tang, Y., Ji, L., Zhu, R., Wei, Z. & Zhang, B. Photodissociation Study of 1,3-Dibromopropane at 234 nm via an Ion Velocity Imaging Technique. *J. Phys. Chem. A* **109**, 11123-11126 (2005).

[53] Streibel, T. & Zimmermann, R. Resonance-Enhanced Multiphoton Ionization Mass Spectrometry (REMPI-MS): Applications for Process Analysis. *Annu. Rev. Anal. Chem.* **7**, 361-381 (2014).

[54] Lubman, D. M. & Kronick, M. N. Mass spectrometry of aromatic molecules with resonance-enhanced multiphoton ionization. *Anal. Chem.* **54**, 660-665 (1982).

[55] Mott, N. F. & Bragg, W. L. The scattering of electrons by atoms. *Proc. R. soc. Lond. Ser. A-Contain. Pap. Math. Phys. Character* **127**, 658-665 (1997).

[56] Ihee, H., Goodson, B. M., Srinivasan, R., Lobastov, V. A. & Zewail, A. H. Ultrafast Electron Diffraction and Structural Dynamics: Transient Intermediates in the Elimination Reaction of $C_2F_4I_2$. *J. Phys. Chem. A* **106**, 4087-4103 (2002).

[57] Yang, J. *et al.* Femtosecond gas phase electron diffraction with MeV electrons. *Faraday Discuss.* **194**, 563-581 (2016).

[58] James, F. & Roos, M. Minuit - a system for function minimization and analysis of the parameter errors and correlations. *Comput. Phys. Commun.* **10**, 343-367 (1975).

[59] Park, J. W. & Shiozaki, T. On-the-Fly CASPT2 Surface-Hopping Dynamics. *J. Chem. Theory Comput.* **13**, 3676-3683 (2017).

[60] Andersson, K., Malmqvist, P. Å. & Roos, B. O. Second-order perturbation theory with a complete active space self-consistent field reference function. *J. Chem. Phys.* **96**, 1218-1226 (1992).

[61] Yanai, T., Tew, D. P. & Handy, N. C. A new hybrid exchange–correlation functional using the Coulomb-attenuating method (CAM-B3LYP). *Chem. Phys. Lett.* **393**, 51-57

(2004).
[62] Weigend, F. & Ahlrichs, R. Balanced basis sets of split valence, triple zeta valence and quadruple zeta valence quality for H to Rn: Design and assessment of accuracy. *Phys. Chem. Chem. Phys.* **7**, 3297-3305 (2005).
[63] Kendall, R. A., Dunning, T. H., Jr. & Harrison, R. J. Electron affinities of the first-row atoms revisited. Systematic basis sets and wave functions. *J. Chem. Phys.* **96**, 6796-6806 (1992).
[64] Kötting, C., Diau, E. W. G., Sølling, T. I. & Zewail, A. H. Coherent Dynamics in Complex Elimination Reactions: Experimental and Theoretical Femtochemistry of 1,3-Dibromopropane and Related Systems. *J. Phys. Chem. A* **106**, 7530-7546 (2002).
[65] Tully, J. C. Mol Simul. *J. Chem. Phys.* **93**, 1061-1071 (1990).
[66] Chong, D. P. *Recent Advances in Density Functional Methods*.
[67] Barbatti, M. & Sen, K. Effects of different initial condition samplings on photodynamics and spectrum of pyrrole. *Int. J. Quantum Chem.* **116**, 762-771 (2016).
[68] Wigner, E. On the Quantum Correction For Thermodynamic Equilibrium. *Phys. Rev.* **40**, 749-759 (1932).
[69] Verlet, L. Computer "Experiments" on Classical Fluids. I. Thermodynamical Properties of Lennard-Jones Molecules. *Phys. Rev.* **159**, 98-103 (1967).
[70] Swope, W. C., Andersen, H. C., Berens, P. H. & Wilson, K. R. A computer simulation method for the calculation of equilibrium constants for the formation of physical clusters of molecules: Application to small water clusters. *J. Chem. Phys.* **76**, 637-649 (1982).
[71] Fukui, K. The path of chemical reactions - the IRC approach. *Acc. Chem. Res.* **14**, 363-368 (1981).
[72] Gaussian 16 Rev. C.01 (Wallingford, CT, 2016).
[73] Shiozaki, T. BAGEL: Brilliantly Advanced General Electronic-structure Library. *Wiley Interdiscip. Rev. Comput. Mol. Sci.* **8**, e1331 (2018).
[74] Møller, K. B. & Zewail, A. H. Femtosecond dynamics of transition states: the classical saddle-point barrier reactions. *Chem. Phys. Lett.* **295**, 1-10 (1998).
[75] Kjær, K. S. *et al.* Finding intersections between electronic excited state potential energy surfaces with simultaneous ultrafast X-ray scattering and spectroscopy. *Chem. Sci.* **10**, 5749-5760 (2019).
[76] Wolf, T. J. A. *et al.* Hexamethylcyclopentadiene: time-resolved photoelectron spectroscopy and ab initio multiple spawning simulations. *Phys. Chem. Chem. Phys.* **16**, 11770-11779 (2014).
[77] Ho, J. W., Chen, W. K. & Cheng, P. Y. Femtosecond pump-probe photoionization-photofragmentation spectroscopy: photoionization-induced twisting and coherent vibrational motion of azobenzene cation. *J. Chem. Phys.* **131**, 134308 (2009).
[78] Ridente, E. *et al.* Femtosecond symmetry breaking and coherent relaxation of methane cations via x-ray spectroscopy. *Science* **380**, 713-717 (2023).
[79] Reiss, G. J., Wyshusek, M. & Rheinländer, J. C. An I_6^{2-} anion in the crystal structure of theophyllinium triiodide monohydrate, $C_7H_{11}I_3N_4O_3$. **237**, 1017-1020 (2022).

Supplementary Figures

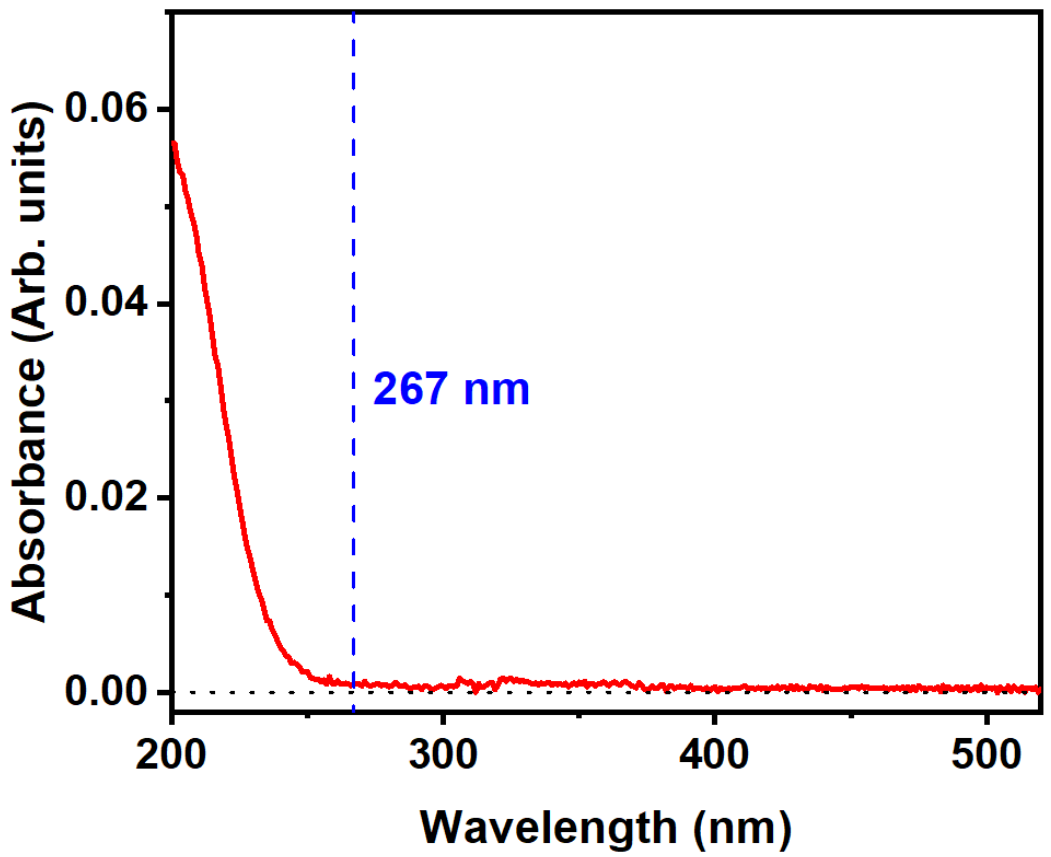

Supplementary Fig. 1. UV-visible spectrum of gas-phase DBP. The wavelength of 267 nm used in the experiment is indicated with a vertical blue dashed line. No absorbance was detected at 267 nm, indicating that any photoreaction detectable via UED originated from the absorption of two or more photons.

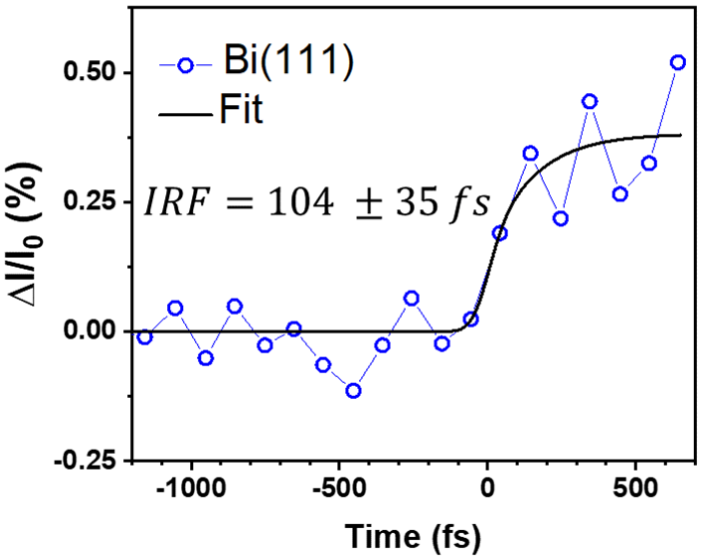

Supplementary Fig. 2. Time-resolved intensities of the 25-nm-thick Bi(111) sample collected to estimate the instrument response function (IRF) of our MeV-UED experiment. The trace was modeled using a convolution between an exponential decay and a Gaussian distribution, representing the intrinsic response of the sample and the temporal resolution of the instrument, respectively. Considering the established 150 fs response of the Bi(111) sample, the resulting IRF was estimated to be 104 ± 35 fs FWHM.

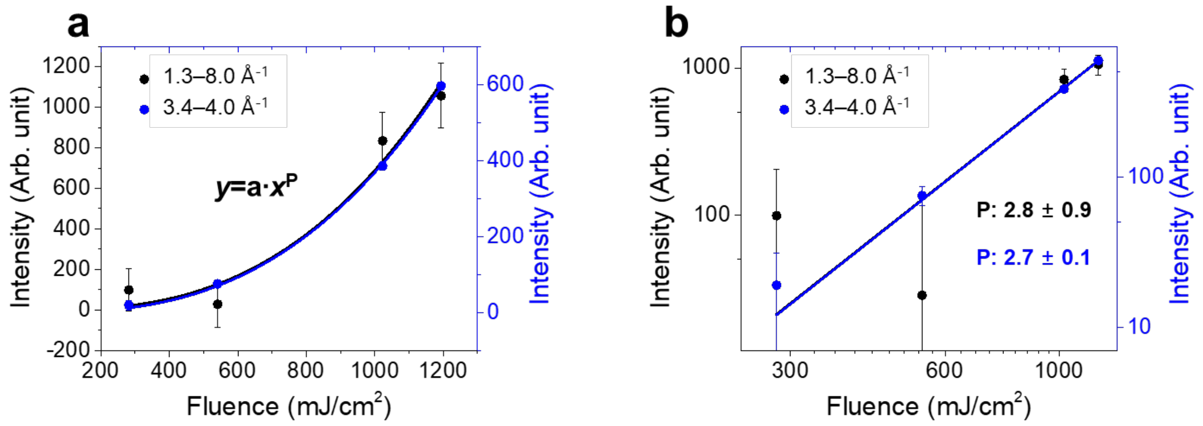

Supplementary Fig. 3. The dependence of the difference scattering intensity on the pump laser fluence. (a, b) The summation of intensity of the difference scattering curve ($s\Delta I_0$) at 45 ps plotted as a function of the pump laser fluence in the linear scale (a) and logarithmic scale (b). To check the effect of signal-to-noise ratio on the dependency, the integrated values are provided for two different *s* regions: from 1.3 to 8.0 $Å^{-1}$ and from 3.4 to 4.0 $Å^{-1}$, allowing for direct comparison. To investigate the laser fluence dependency, each integrated data was fitted with a power function ($y = a \cdot x^P$). The fitted exponent, P in the equation in (a), is 2.8 ± 0.9 and 2.7 ± 0.1, for the two *s* regions, respectively, indicating that the observed difference scattering signals originated from a process involving three photons.

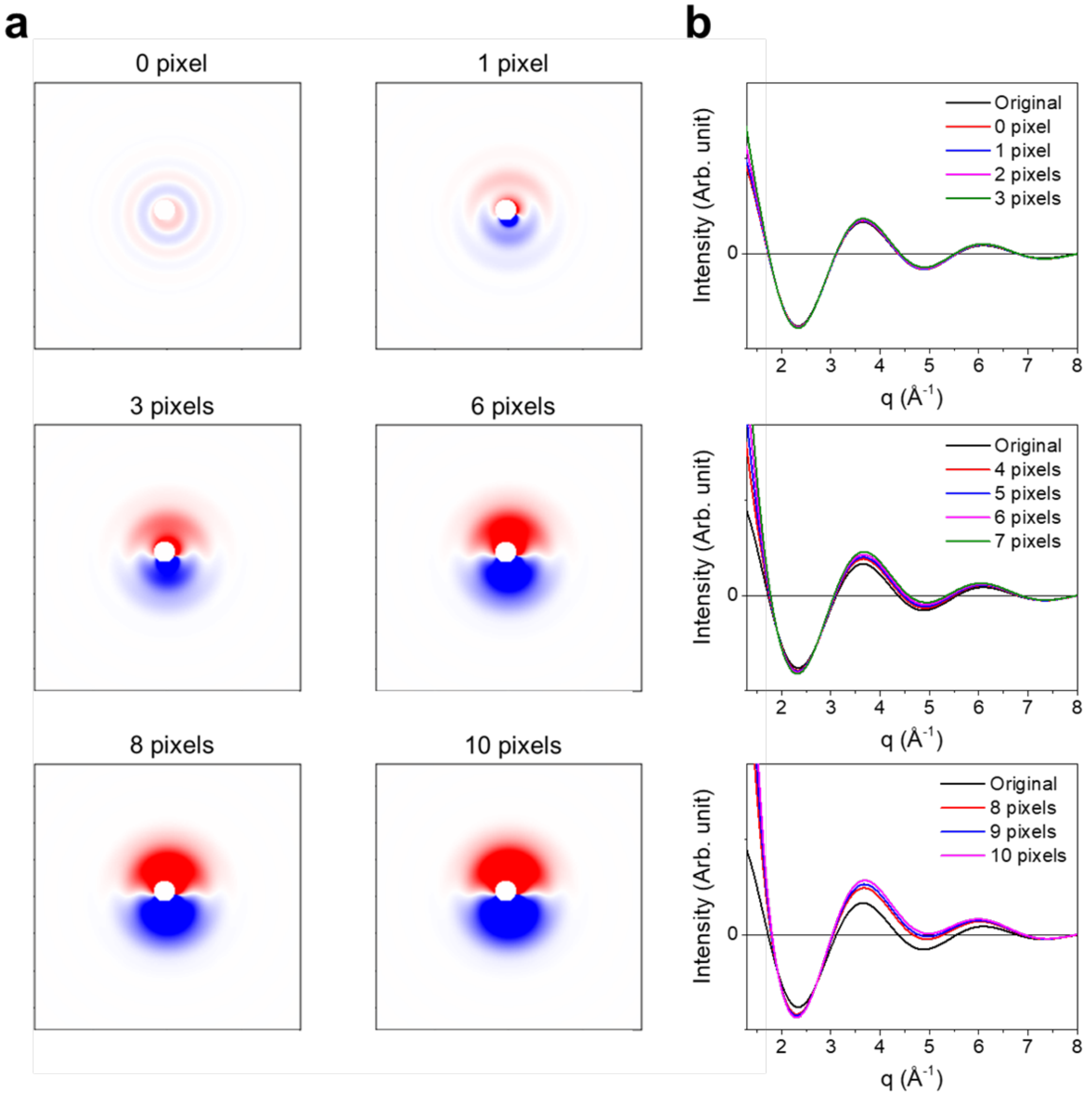

Supplementary Fig. 4. Mock data analysis to assess the validity of Legendre decomposition in the case of beam shift. (a) Mock difference scattering patterns corresponding to various pixel shifts (0, 1, 3, 6, 8, and 10) are presented. These patterns are generated by subtracting center-shifted excited-state signals from the ground-state scattering signal. In the mock data, we replicated the presence of a blocked signal region caused by the beam block. To mimic the experimental conditions, we intentionally shifted the blocked region by about 20 pixels away from the center of the scattering pattern. **(b)** The ΔI_0 curves obtained by applying Legendre decomposition to the mock data shown in (a). The ΔI_0 curves are shown for different extent of beam deflection, and compared with the theoretical curve corresponding to the pure structural change.

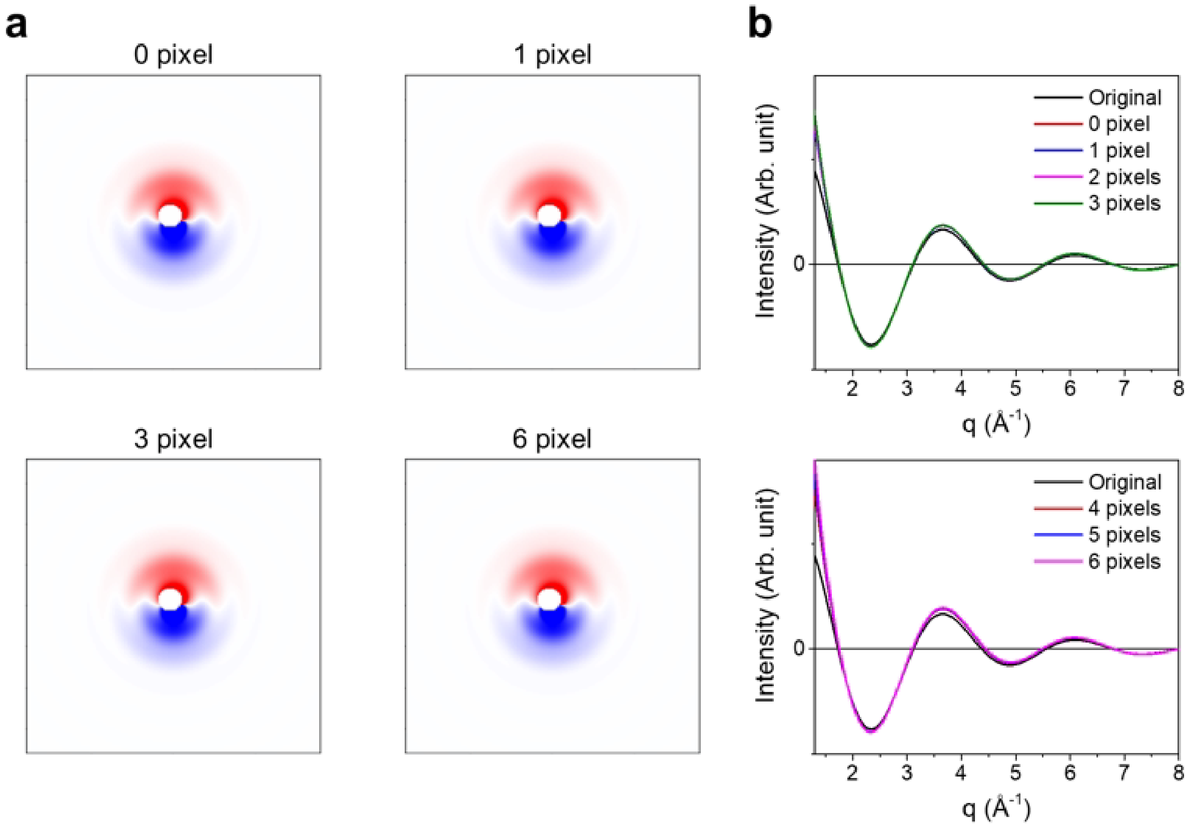

Supplementary Fig. 5. Mock data analysis to assess the validity of Legendre decomposition in the case of beam broadening. (a) Mock difference scattering patterns corresponding to various beam broadening (ΔFWHM = 0, 1, 3, and 6 pixels) are presented. The beam shift is fixed at 3 pixels, which is the maximum observed in our UED data. These patterns are generated by subtracting broadened excited-state signals from the unbroadened ground-state scattering signal. In the mock data, we replicated the presence of a blocked signal region caused by the beam block. To mimic the experimental conditions, we intentionally shifted the blocked region by about 20 pixels away from the center of the scattering pattern. **(b)** The ΔI_0 curves obtained by applying Legendre decomposition to the mock data shown in (a). The ΔI_0 curves are shown for different extent of beam broadening, and compared with the theoretical curve corresponding to the pure structural change.

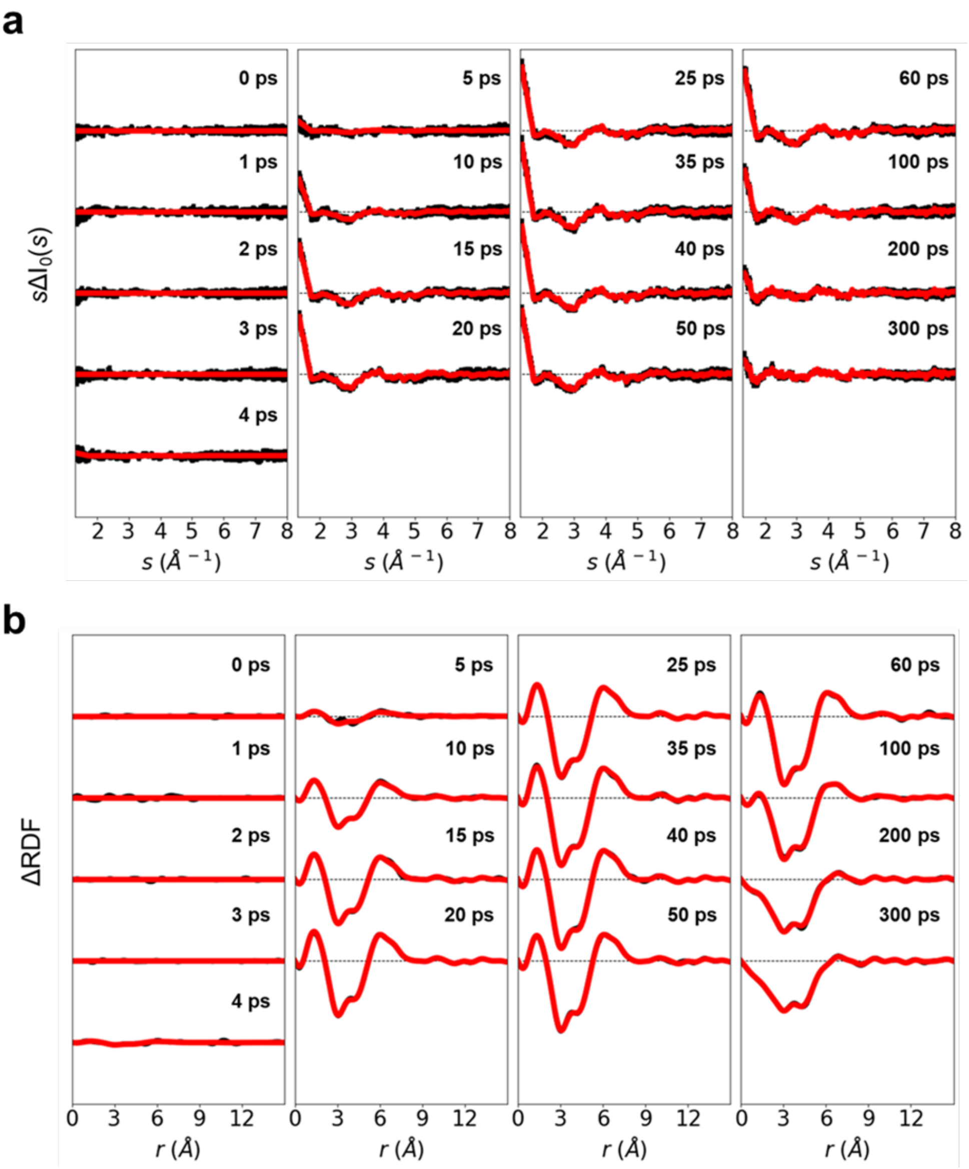

Supplementary Fig. 6. Comparison on experimental and simulated data obtained based on the best-fit kinetic model determined from the kinetics-constrained analysis (KCA). (a) Simulated results presented in *s*-space ($s\Delta I_0(s)$). Experimental curves, along with their corresponding standard errors (vertical bars), are depicted in black, while theoretical curves are shown in red lines. **(b)** simulated results shown in real space (ΔRDF). The data, as presented in (a), have been transformed into real space data by applying. Here, experimental ΔRDFs are depicted in black, and theoretical ΔRDF curves are represented by red lines.

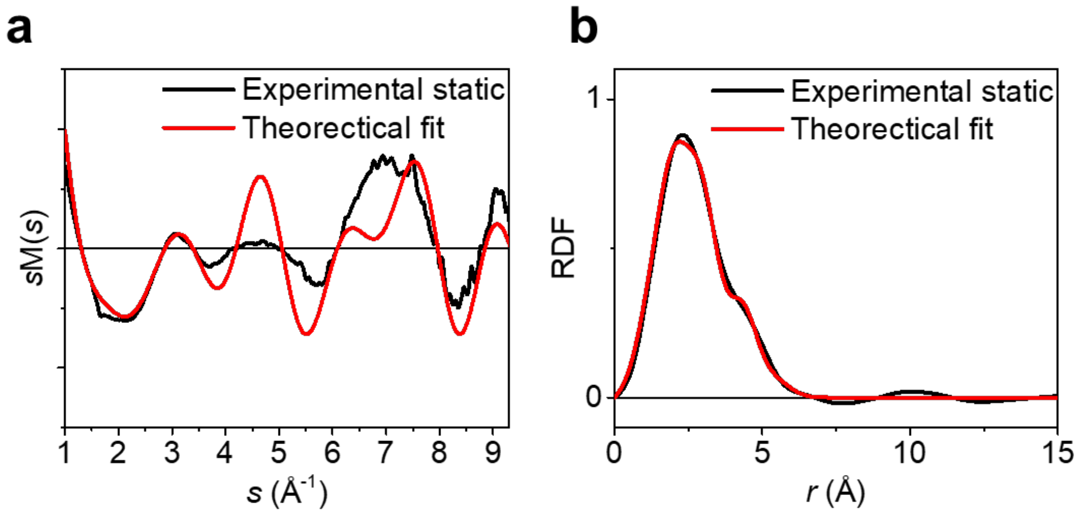

Supplementary Fig. 7. Analysis of the static scattering curve. **(a)** The modified scattering curve (*s*M(*s*)) of the ground state of neutral DBP obtained from the experiment (black) and the theoretical fit (red). To analyze the fractions of ground state conformers, *s*M(*s*) was fitted with a sum of the theoretically calculated curves of GG, AG, and AA conformers. **(b)** RDFs of *s*M(*s*). The ratios of conformers were determined as GG:AG:AA = 66%:20%:14%.

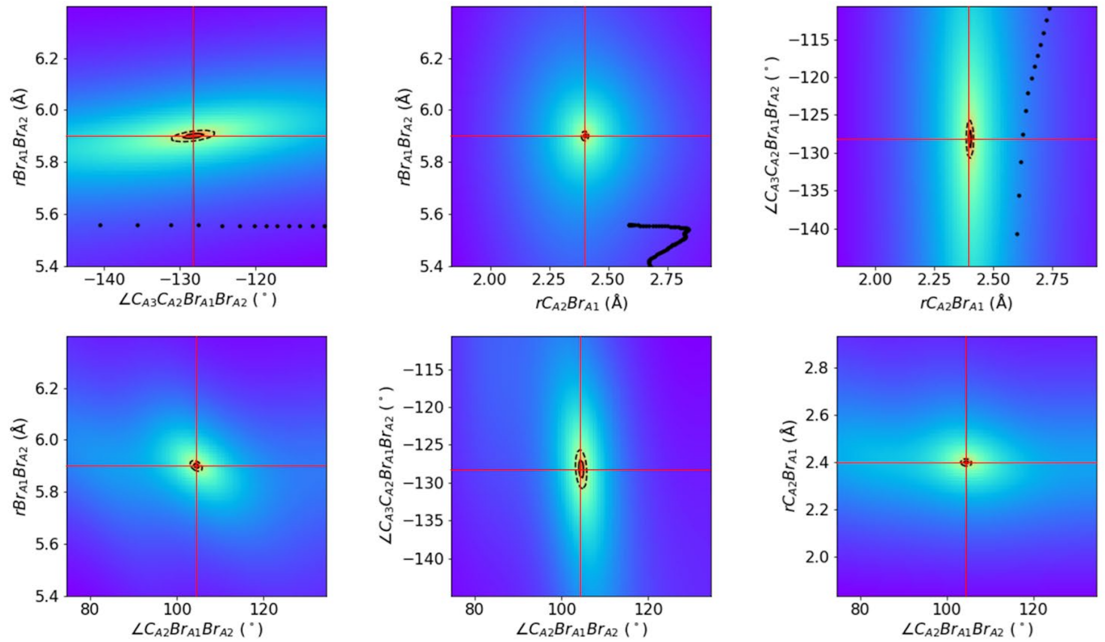

Supplementary Fig. 8. Color maps illustrating the variation of the adjusted χ^2, represented as $\Delta\chi^2$ (= χ^2 - χ^2_{min}), with respect to the structural parameters of species A^+ (*iso*-DBP$^+$). The color maps are shown for the six pairs of structural parameters generated from the four selected parameters (rBr$_{A1}$Br$_{A2}$, rC$_{A2}$Br$_{A1}$, ∠C$_{A2}$Br$_{A1}$Br$_{A2}$, and ∠C$_{A3}$C$_{A2}$Br$_{A1}$Br$_{A2}$) among the total of seven parameters (see Supplementary Table 1 for the complete list) used in the structural refinement of species A^+. The color spectrum ranges from red to blue, with red denoting small $\Delta\chi^2$ values, indicating excellent agreement between experimental and theoretical curves. Conversely, blue represents large $\Delta\chi^2$ values, indicating poor agreement between the theoretical and experimental data. Intermediate regions are depicted in green.The black solid and dashed lines indicate contours where the χ^2 value increases by 1 and 4 from its minimum value ($\Delta\chi^2$ = 1 and 4), corresponding to 1σ and 2σ error ranges. The vertical and horizontal red lines indicate the structural parameters optimized through the structural refinement process. Black dots mark structural parameters corresponding to structures extracted from AIMD snapshots. In some cases, the structures from AIMD simulations lie beyond the covered structural ranges in these plots, rendering them not visible in the figure. In the most extreme cases (the three lower panels), none of the snapshots obtained from AIMD simulations fall within the range covered by the respective panel, resulting in the absence of black dots in those panels.

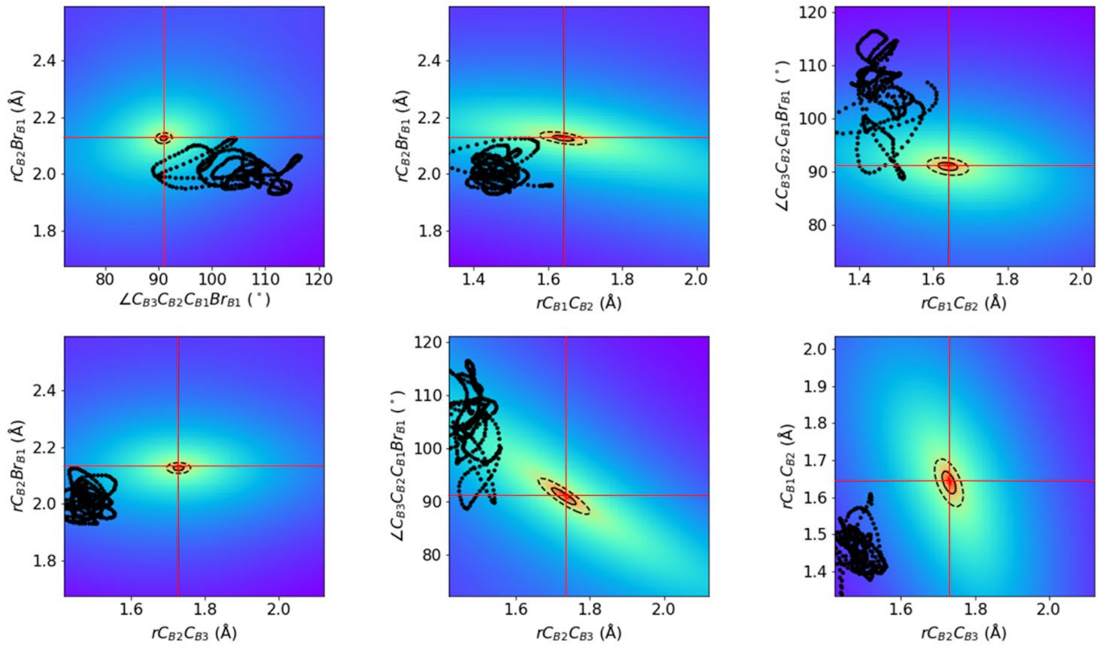

Supplementary Fig. 9. **Color maps showing the variation of the adjusted χ^2 (i.e. $\Delta\chi^2 = \chi^2 - \chi^2_{min}$) with respect to a pair of correlated parameters among the four parameters (rCB2BrB1, ∠CB3CB2CB1BrB1, rCB1CB2, rCB2CB3) used in the structural refinement of species B$^+$ (bromonium MBP$^+$).** The color spectrum ranges from red to blue, with red denoting small $\Delta\chi^2$ values, indicating excellent agreement between experimental and theoretical curves. Conversely, blue represents large $\Delta\chi^2$ values, indicating poor agreement between the theoretical and experimental data. Intermediate regions are depicted in green.The black solid and dashed lines indicate contours where the χ^2 value increases by 1 and 4 from its minimum value ($\Delta\chi^2$ = 1 and 4), corresponding to 1σ and 2σ error ranges. The vertical and horizontal red lines indicate the structural parameters optimized through the structural refinement process. Black dots mark structural parameters corresponding to structures extracted from AIMD snapshots.

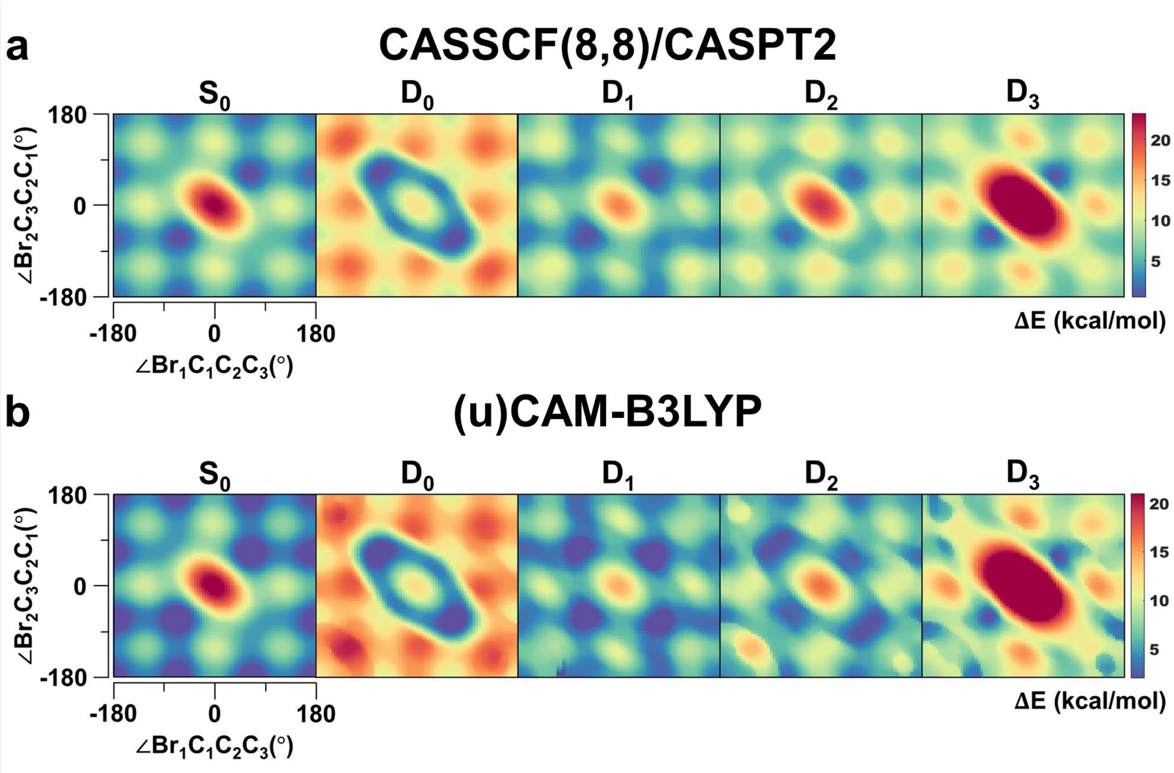

Supplementary Fig. 10. Comparison of the PESs using two different calculational methods. (a) PESs from a (SA-)CASSCF(8,8)/(XMS-)CASPT2 calculations. **(b)** PESs from the DFT calculation with (u)CAM-B3LYP functional using def2-TZVPP basis set. The PESs of S_0, (the ground state of neutral DBP), D_0, D_1, D_2 and D_3 (the first four states of DBP^+) were explored. The overall features of the PESs in (a) and (b) are similar, indicating that the DFT calculation level used in (b) is suitable for exploring the PESs of DBP. This also supports the choice of the functional and basis sets used in the surface hopping simulation, in which the equivalent DFT calculation level as (b) was employed.

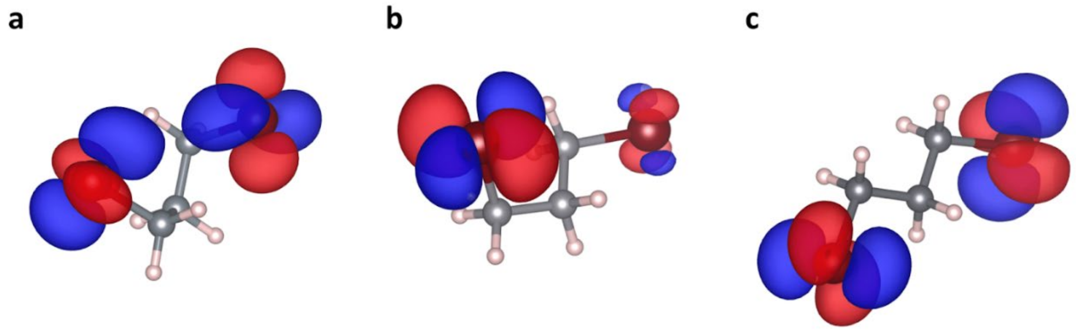

Supplementary Fig. 11. Dyson orbitals. (a–c) Dyson orbitals of DBP computed between the D_2 state of DBP^+ and the first Rydberg state (Ryd in Extended Data Table 1) of DBP. The Dyson orbitals are presented for the (a) GG, (b) AG, and (c) AA conformer of DBP. The orbitals are plotted using an isovalue of 0.03. The Dyson orbitals pertaining to other D states have highly similar shapes to those shown here, except that they have slightly different iso densities.

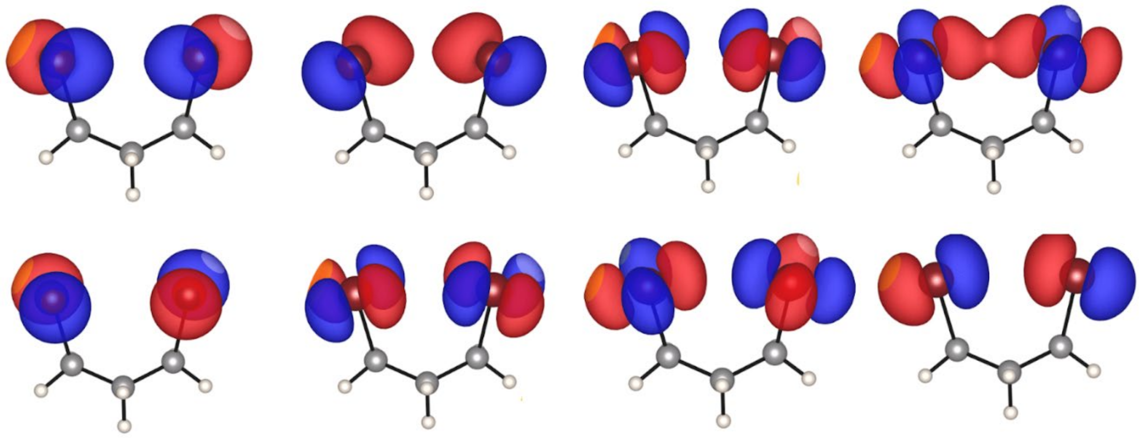

Supplementary Fig. 12. CAS orbitals. SA(4)-CASSCF orbitals employed in the XMS-CASPT2 calculations for DBP^{+}. An analogous active space has been used for the calculation on the ground state CASSCF calculations for DBP.

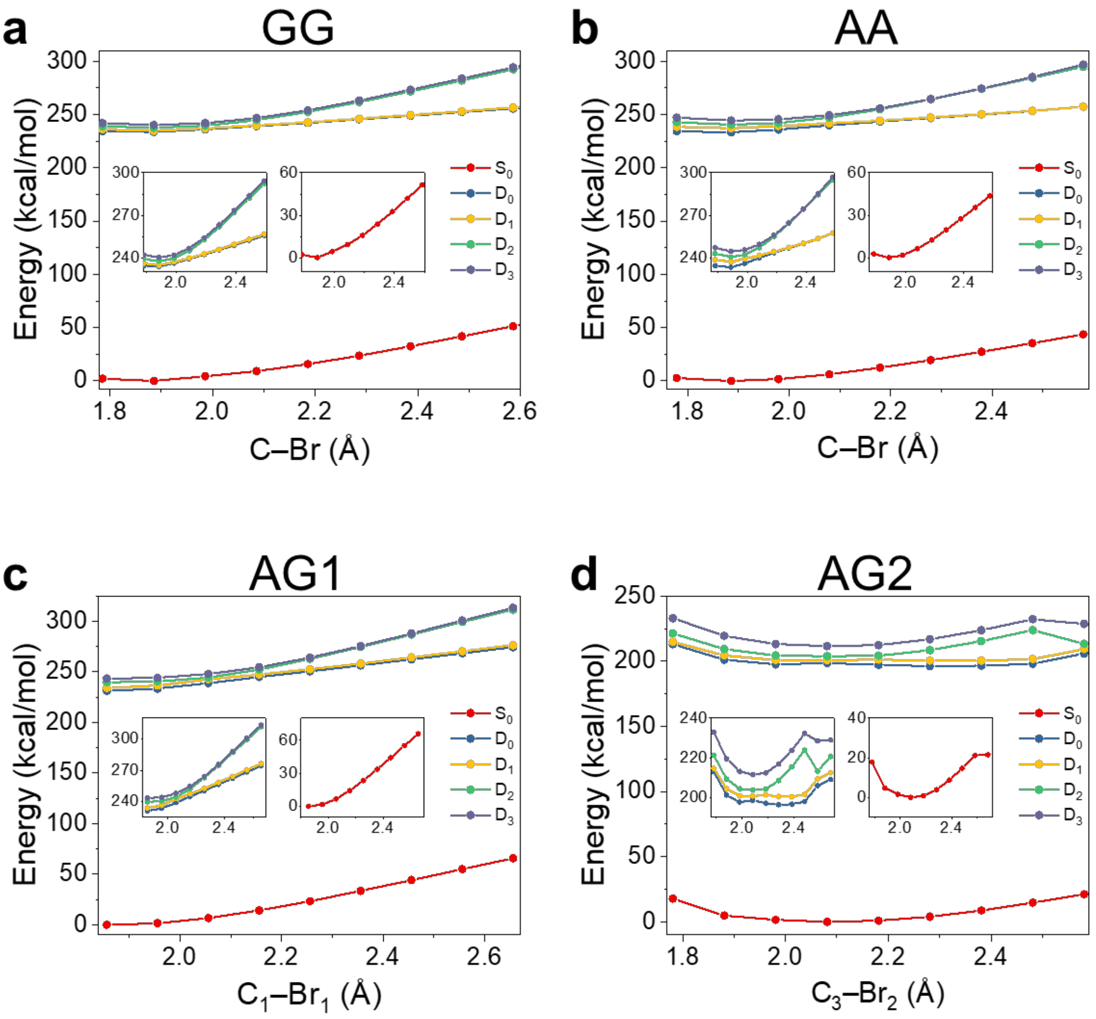

Supplementary Fig. 13. Potential energy surfaces (PES) of S_0 (neutral ground state), D_0 (first doublet state), D_1, D_2 and D_3 state of DBP toward the C–Br distance. (a-d) 1D PESs regarding the three conformers of DBP and DBP^+, (a) GG, (b) AA and (c, d) two C–Br pairs of AG. The energies here are calculated using (XMS-)CASPT2 corrections of the (SA-)CASSCF(8,8) energies and represented on a relative scale with respect to the global minima of the S_0 state.

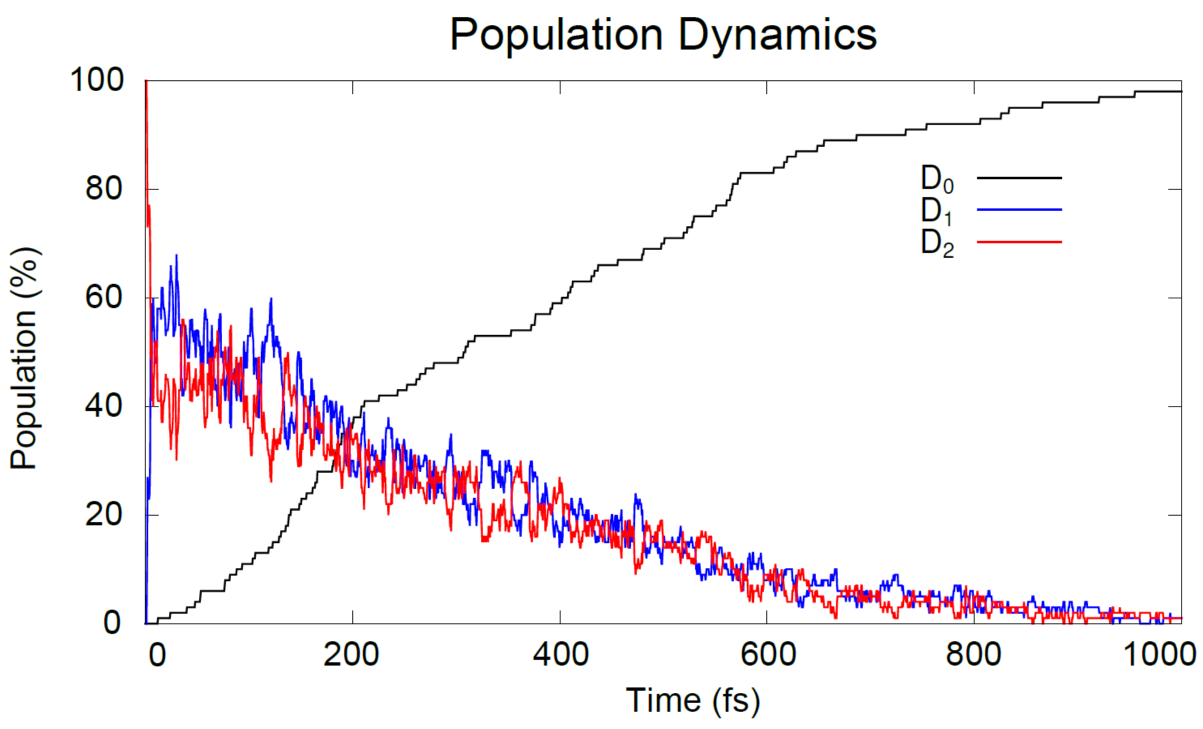

Supplementary Fig. 14. Population changes of the excited states of DBP^+ during the trajectory surface hopping simulation. Average state populations of D_0, D_1, and D_2, derived from 100 NAMD trajectories, are represented by black, blue, and red colors, respectively. These trajectories were computed using 100 initial conditions sampled via the Wigner distribution of the ground state (S_0) of the GG isomer of DBP.

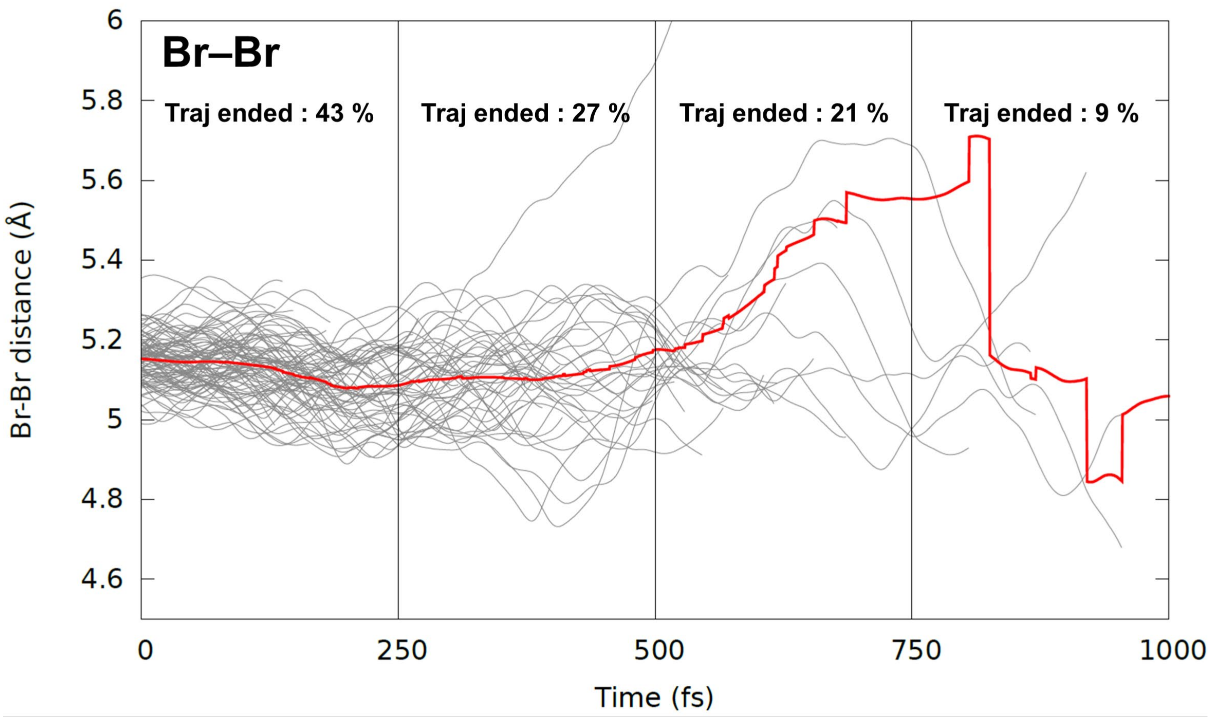

Supplementary Fig. 15. The distance trajectory map of Br–Br of the excited states of DBP^+ during the trajectory surface hopping simulation. Each of the gray lines represents an individual trajectory, and the average of the trajectories is shown as a red solid line. Since the simulation ends when a trajectory reaches the D_0 state, only the trajectories that have not ended are included in the red line.

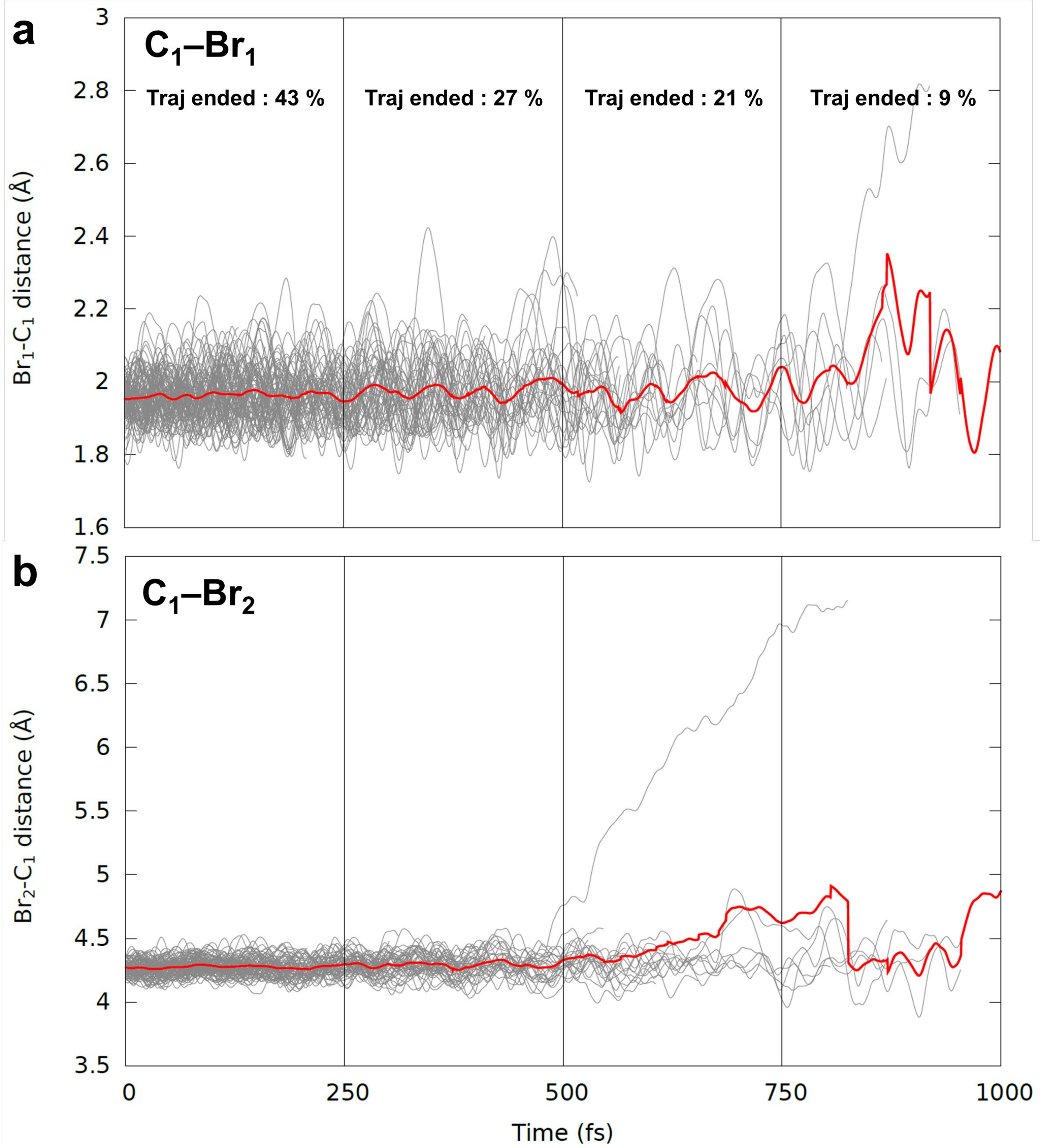

Supplementary Fig. 16. The distance trajectory maps of C_1–Br_1 and C_1–Br_2 of the excited states of DBP^+ during the trajectory surface hopping simulation. (a, b) Distance maps of the two different C_1–Br pairs. Each of the gray lines represents an individual trajectory, and the average of the trajectories is shown as a red solid line. Since the simulation ends when a trajectory reaches the D_0 state, only the trajectories that have not ended are included in the red line.

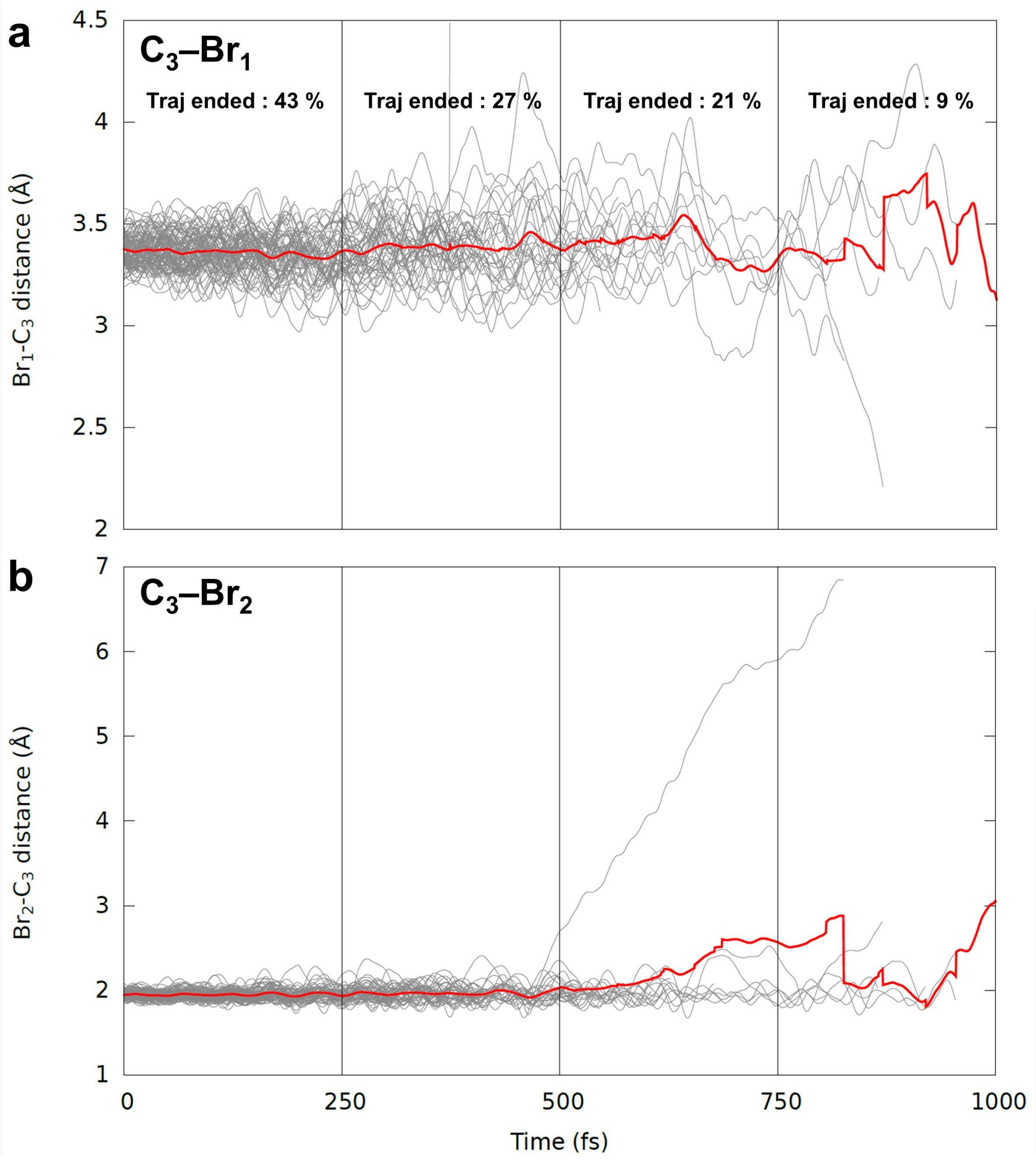

Supplementary Fig. 17. The distance trajectory maps of C_3–Br_1 and C_3–Br_2 of the excited states of DBP^+ during the trajectory surface hopping simulation. (a, b) Distance map of the two different C_3–Br pairs. Each of the gray lines represents an individual trajectory, and the average of the trajectories is shown as a red solid line. Since the simulation ends when a trajectory reaches the D_0 state, only the trajectories that have not ended are included in the red line.

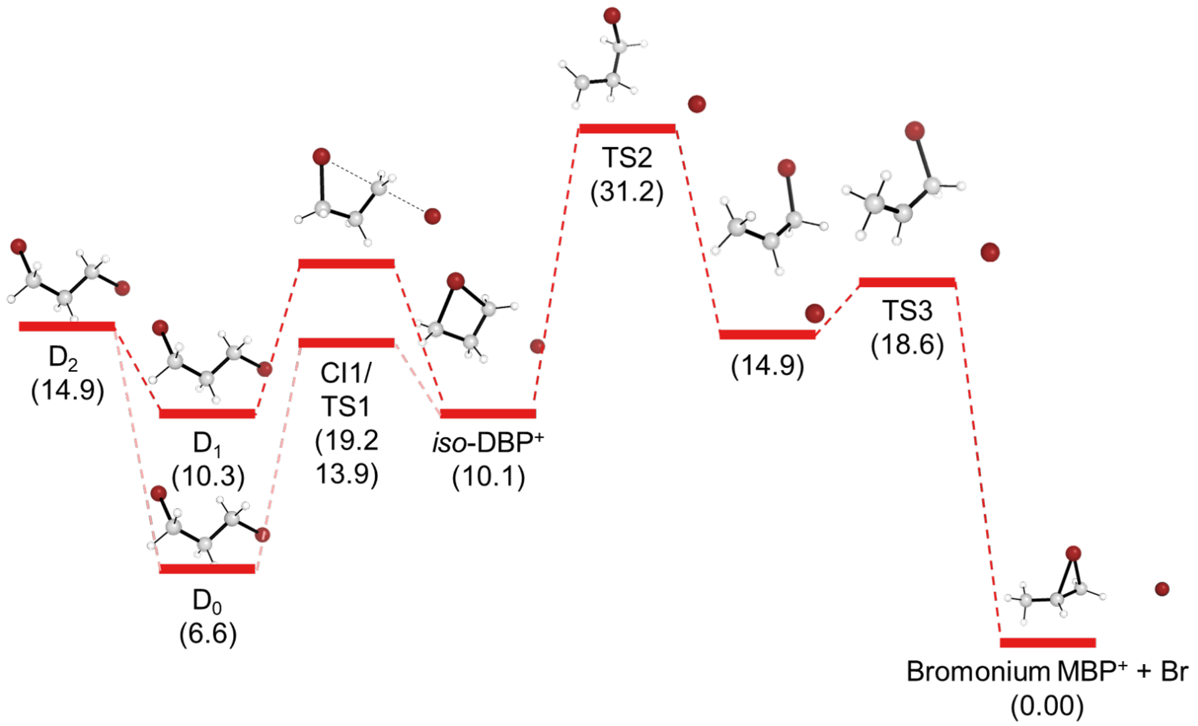

Supplementary Fig. 18. Key stationary points on the doublet surface for DBP$^+$ relevant to the formation of *iso*-DBP$^+$ and bromonium MBP$^+$. The units of relative free energies here are kcal/mol. For each of the transition states, an IRC calculation was conducted to confirm their significance to the reaction pathway. For D_0, D_1 and D_2, the energies for the most stable conformer for the neutral ground state (S_0), namely the GG conformer, are shown.

Supplementary Tables

Supplementary Table 1. Structural parameters of *iso*-DBP$^+$ and bromonium MBP$^+$ optimized from the structural fitting analysis. Independent parameters are shown in black and dependent ones are shown in blue.

Intermediate	Parameters	Value
Ground state DBP	GG fraction (%)	63 ± 3*
	AG fraction (%)	20 ± 4*
	AA fraction (%)	17 ± 5*
iso-DBP$^+$ (Species A$^+$)	$rBr_{A1}Br_{A2}$ (Å)	5.90 ± 0.02
	$rC_{A2}Br_{A1}$ (Å)	2.40 ± 0.04
	$\angle C_{A1}C_{A2}C_{A3}Br_{A1}$ (°)	9.5 ± 3.4
	$\angle C_{A1}C_{A2}C_{A3}$ (°)	91.3 ± 2.3
	$rC_{A1}C_{A2}$ ($rC_{A2}C_{A3}$) (Å)	1.28 ± 0.03
	$\angle C_{A2}Br_{A1}Br_{A2}$ (°)	104.5 ± 1.3
	$\angle C_{A3}C_{A2}Br_{A1}Br_{A2}$ (°)	-128.19 ± 2.5
	$rC_{A1}Br_{A1}$ (Å)	1.76 ± 0.01
	$rC_{A1}C_{A3}$ (Å)	1.77 ± 0.06
Bromonium MBP$^+$ (Species B$^+$)	$rC_{B2}Br_{B1}$ (Å)	2.12 ± 0.04
	$\angle C_{B3}C_{B2}C_{B1}Br_{B1}$ (°)	91.0 ± 2.8
	$rC_{B1}C_{B2}$ (Å)	1.64 ± 0.06
	$rC_{B2}C_{B3}$ (Å)	1.73 ± 0.08
	$\angle C_{B2}C_{B1}Br_{B1}$ (°)	67.2 ± 1.3

*These fractions were obtained from the simultaneous fitting of the SADSs of the two species (*iso*-DBP$^+$ and bromonium MBP$^+$). These fractions are highly similar to those obtained from the fitting of the static curve of DBP (66 ± 2.3%: 20 ± 1.5%: 14 ± 2.7% for GG, AG, and AA).

Supplementary Table 2. Influence of beam deflection on structural parameter determination. The influence of beam deflection on the determination of structural parameters was explored. Independent parameters are shown in black, and dependent ones are shown in blue. The values corresponding to "Original" are identical to those presented in Supplementary Table 1.

Intermediate	Parameters	Original	3 pixel corrected	6 pixel corrected
Ground state DBP	GG fraction (%)	63 ± 3*	52 ± 3*	42 ± 3*
	AG fraction (%)	20 ± 4*	23 ± 3*	24 ± 3*
	AA fraction (%)	17 ± 5*	25 ± 5*	34 ± 5*
iso-DBP+ (Species A+)	$rBr_{A1}Br_{A2}$ (Å)	5.90 ± 0.02	5.88 ± 0.02	5.88 ± 0.02
	$rC_{A2}Br_{A1}$ (Å)	2.40 ± 0.04	2.39 ± 0.04	2.41 ± 0.05
	$\angle C_{A1}C_{A2}C_{A3}Br_{A1}$ (°)	9.5 ± 3.4	7.1 ± 3.6	4.8 ± 4.4
	$\angle C_{A1}C_{A2}C_{A3}$ (°)	91.3 ± 2.3	94.8 ± 2.6	98.2 ± 3.2
	$rC_{A1}C_{A2}$ ($rC_{A2}C_{A3}$) (Å)	1.28 ± 0.03	1.34 ± 0.03	1.31 ± 0.03
	$\angle C_{A2}Br_{A1}Br_{A2}$ (°)	104.5 ± 1.3	106.4 ± 1.3	106.21 ± 1.4
	$\angle C_{A3}C_{A2}Br_{A1}Br_{A2}$ (°)	-128.19 ± 2.5	-130.6 ± 2.2	-135.4 ± 2.2
	$rC_{A1}Br_{A1}$ (Å)	1.77 ± 0.01	1.78 ± 0.01	1.84 ± 0.01
	$rC_{A1}C_{A3}$ (Å)	1.83 ± 0.06	1.97 ± 0.06	1.98 ± 0.06
Bromonium MBP+ (Species B+)	$rC_{B2}Br_{B1}$ (Å)	2.12 ± 0.04	2.10 ± 0.04	2.09 ± 0.04
	$\angle C_{B3}C_{B2}C_{B1}Br_{B1}$ (°)	91.0 ± 2.8	91.2 ± 3.3	91.8 ± 4.2
	$rC_{B1}C_{B2}$ (Å)	1.64 ± 0.06	1.63 ± 0.07	1.62 ± 0.08
	$rC_{B2}C_{B3}$ (Å)	1.73 ± 0.08	1.72 ± 0.10	1.68 ± 0.10
	$\angle C_{B2}C_{B1}Br_{B1}$ (°)	67.2 ± 1.3	67.1 ± 1.0	67.1 ± 1.3

*These fractions were obtained from the simultaneous fitting of the SADSs of the two species (*iso*-DBP$^+$ and bromonium MBP$^+$). These fractions are highly similar to those obtained from the fitting of the static curve of DBP (66 ± 2.3%: 20 ± 1.5%: 14 ± 2.7% for GG, AG, and AA).

Supplementary Table 3. Calculated geometric parameters for the ground State and ionic States of DBP. Geometric parameters of DBP and its ionic states (doublet) optimized at the CASPT2 and XMS-CASPT2 level, respectively, using a CAS(8,8), aug-cc-pvtz basis set, and second-order Douglas-Kroll-Hess Hamiltonian. Supplementary Fig. 12 presents the active space utilized for (SA-)CASSCF/(XMS-)CASPT2 calculations, whereas Fig. 4c highlights the geometric parameters analyzed in the DBP structures. The geometries and energies are for the global minima of each state, whereas for S_0, those for all three conformers (AA, AG, and GG) are listed. For S_0, the energies are shown for all three conformers, and for D_0–D_3, the energies for only global minima are shown. Note that for D_1 and D_2, the global minimum has a similar structure to the AG and GG conformers of S_0, respectively, whereas the global minima for D_0 and D_3 are at conformations completely different from the three conformers (AA, AG, and GG) of the neutral ground state.

Electronic State		C_1-C_2 (Å)	C_2-C_3 (Å)	C_1-Br_1 (Å)	C_3-Br_2 (Å)	Br_1-Br_2 (Å)	C_1-C_2-C_3 (°)	Br_1-C_1-C_3-Br_2 (°)	Br_1-C_1-C_2-C_3 (°)	Br_2-C_3-C_2-C_1 (°)	Energy (kcal/mol)
S_0	AA	1.506	1.506	1.879	1.879	5.566	108.5	0.004	180.0	180.0	3.597
	AG	1.506	1.503	1.880	1.874	4.798	111.3	110.4	63.33	172.2	2.749
	GG	1.504	1.504	1.886	1.886	4.046	111.7	110.6	61.05	61.05	0
D_0		1.529	1.506	1.893	1.893	2.717	112.3	0.002	65.83	65.83	206.8
D_1		1.529	1.530	1.949	1.953	3.800	113.5	94.05	51.19	52.22	232.7
D_2		1.505	1.507	1.895	1.857	4.877	112.9	143.0	49.61	174.8	240.6
D_3		1.503	1.525	1.903	1.866	4.080	111.9	57.11	66.70	135.0	244.8

Supplementary Table 4. Natural Bond Orbital (NBO) analysis. The charges are obtained from DFT calculations. For the DFT calculation, a CAM-B3LYP functional was used with a def2-TZVPP basis set.

	DBP			DBP^+			*iso*-DBP^+ (Species A^+)	Bromonium MBP^+ (Species B^+)
	GG	AG	AA	GG	AG	AA		
C_1	-0.409	-0.412	-0.408	-0.216	-0.211	-0.438	-0.284	-0.677
H_1	0.222	0.212	0.214	0.128	0.133	0.257	0.246	0.287
H_2	0.228	0.227	0.214	0.118	0.128	0.257	0.255	0.250
C_2	-0.452	-0.450	-0.447	-0.192	-0.222	-0.461	-0.490	0.011
H_3	0.224	0.223	0.216	0.128	0.128	0.234	0.267	0.270
H_4	0.224	0.218	0.216	0.128	0.118	0.234	0.247	0.262
C_3	-0.409	-0.404	-0.408	-0.216	-0.223	-0.438	-0.268	-0.354
H_5	0.228	0.225	0.214	0.128	0.126	0.257	0.246	0.266
H_6	0.222	0.213	0.214	0.118	0.143	0.257	0.257	0.274
Br_1	-0.038	-0.033	-0.012	0.438	0.330	0.421	0.502	0.410
Br_2	-0.038	-0.020	-0.012	0.438	0.549	0.421	0.021	-

Supplementary Table 5. XYZ coordinates of the transition states of DBP^+ upon the IRC coordinates. The structures listed in the table are utilized for the TS calculation in Supplementary Fig. 18 and the IRC calculations depicted in Extended Data Fig. 8.

	XYZ coordinates											
	TS1			CI1			TS2			TS3		
C_1	1.0928	0.9715	0.9855	0.9879	0.9348	0.5968	0.8572	1.2303	1.0876	1.8735	-0.1326	0.2623
H_1	1.8510	1.7057	1.2264	1.6501	1.7561	0.3500	1.8391	0.7781	0.9897	2.8557	0.2795	0.5281
H_2	0.8631	0.3527	1.8446	1.1604	0.5732	1.6038	0.6060	1.6617	2.0538	1.9037	-0.8884	-0.5151
C_2	-0.1390	1.5429	0.3002	-0.4839	1.2384	0.2921	-0.0321	1.3494	-0.0139	0.9500	0.9362	0.0572
H_3	0.1052	2.4391	-0.2639	-0.6214	2.2584	-0.0531	0.0049	2.4399	-0.2579	1.5663	-0.6005	1.2190
H_4	-0.9021	1.7875	1.0392	-1.0932	1.1184	1.1837	-1.0764	1.2596	0.3349	0.9691	1.7605	0.7743
C_3	-0.5921	0.4551	-0.6311	-0.9416	0.2705	-0.7789	0.2811	0.3936	-1.1407	-0.0083	1.0453	-1.0209
H_5	-0.4805	0.5431	-1.6990	-0.7098	0.4374	-1.8338	1.2047	0.6200	-1.6629	0.5663	1.1733	-1.9499
H_6	-0.9350	-0.4901	-0.2372	-1.1087	-0.7728	-0.5804	-0.5334	0.2976	-1.8480	-0.7006	1.8707	-0.9013
Br_1	1.8229	-0.2526	-0.3760	1.4331	-0.5725	-0.6733	0.5238	-1.3259	-0.2448	-0.9877	-0.6197	-1.2154
Br_2	-3.0017	1.1583	-0.9620	-3.3215	0.7204	-1.1556	-3.6751	0.6411	0.7025	-0.1019	4.2274	1.6347

Supplementary Table 6. Calculated emission lifetimes. The lifetimes were computed from the ground-state (GS) geometry (GG isomer) using Eq. S17, employing either the energies and oscillator strengths calculated at the CASSCF level or the XMS-CASPT2 level. The radiative lifetimes are expressed in microseconds (μs).

Lifetime (μs)	CASSCF	CASPT2
D3 → D0	0.409	0.238
D2 → D0	183.364	120.823
D1 → D0	7692.303	4584.812

Supplementary Table 7. Vibrational frequencies for optimized DFT structures of the intermediates shown in Supplementary Fig. 18. The modes associated with the displacement of loosely bound Br are highlighted in red. Same functional and basis set were used during the DFT.

iso-DBP⁺		Intermediate b/t TS2 and TS3		Bromonium MBP⁺	
Mode #	Frequency (cm^{-1})	Mode #	Frequency (cm^{-1})	Mode #	Frequency (cm^{-1})
**1**	**35**	**1**	**12**	1	207
**2**	**42**	2	32	2	233
**3**	**45**	**3**	**57**	3	379
4	139	4	131	4	416
**5**	**365**	5	170	5	516
**6**	**451**	6	220	6	869
7	648	7	481	7	936
8	810	8	551	8	955
9	827	9	585	9	987
10	959	10	694	10	1065
11	988	11	946	11	1148
12	1024	12	1001	12	1224
13	1036	13	1097	13	1241
14	1201	14	1119	14	1407
15	1208	15	1250	15	1424
16	1263	16	1306	16	1459
17	1272	17	1314	17	1500
18	1362	18	1321	18	1520
19	1477	19	1360	19	3032
20	1492	20	1458	20	3125
21	1510	21	1532	21	3162
22	3111	22	2870	22	3176
23	3151	23	2940	23	3199
24	3160	24	3060	24	3267
25	3166	25	3088		
26	3239	26	3096		
27	3253	27	3184		

Supplementary Table 8. Vibrational frequencies for optimized DFT structures of the transition states shown in Supplementary Fig. 18. The modes associated with the displacement of loosely bound Br are highlighted in red. Each structure exhibits a single imaginary frequency, confirming that each structure corresponds to a transition state.

TS1		TS2		TS3	
Mode #	Frequency (cm^{-1})	Mode #	Frequency (cm^{-1})	Mode #	Frequency (cm^{-1})
**1**	**-258**	1	-217	1	-261
**2**	**98**	**2**	**8**	**2**	**10**
**3**	**112**	**3**	**31**	3	24
**4**	**126**	**4**	**76**	**4**	**66**
5	175	5	223	5	146
**6**	**428**	6	305	6	199
7	623	7	466	7	424
8	806	8	549	8	582
9	845	9	670	9	737
10	866	10	878	10	794
11	970	11	933	11	903
12	1051	12	972	12	959
13	1062	13	1057	13	1083
14	1083	14	1132	14	1171
15	1213	15	1222	15	1249
16	1249	16	1271	16	1266
17	1303	17	1294	17	1324
18	1365	18	1308	18	1389
19	1471	19	1423	19	1422
20	1489	20	1501	20	1443
21	1507	21	1540	21	1539
22	3084	22	2853	22	2943
23	3143	23	2925	23	3008
24	3152	24	3137	24	3031
25	3195	25	3138	25	3109
26	3221	26	3208	26	3181
27	3307	27	3247	27	3189
